# Contextualizing the scope, scale, and speed of energy pathways toward sustainable development in Africa

**DOI:** 10.1016/j.isci.2022.104965

**Published:** 2022-09-06

**Authors:** Ayobami S. Oyewo, Dmitrii Bogdanov, Arman Aghahosseini, Theophilus N.O. Mensah, Christian Breyer

**Affiliations:** 1 LUT University, Yliopistonkatu 34, 53850 Lappeenranta, Finland

**Keywords:** Energy resources, Energy policy, Energy management, Energy Modeling

## Abstract

Faced with interrelated challenges of climate change and energy crises, Africa’s future energy system orientation could be steered toward sustainable development. In this study, we contextualized diverging fossil fuels-dominated and renewable energy-based pathways toward sustainable development in Africa. A novel and sophisticated techno-economic energy modeling tool is used to describe the scope of the pathways in high geo-spatial and full hourly resolution for Africa covering the entire energy system. This study demonstrates that a renewable energy pathway is not only climate-compatible, but also delivers a lower cost system structure than alternative pathways. Our results show that Africa can leapfrog carbonization by using its low-cost renewable electricity and green hydrogen. Furthermore, Africa can become a self-sufficient green economy and an exporter of green fuels. Notably, solar photovoltaic-battery hybrid systems and electrolyzers are instrumental in achieving carbon-neutrality in Africa. This research presents a “true-zero emission” pathway for Africa.

## Highlights

• A true-zero emission pathway for Africa
• PV-battery hybrid systems drive deep decarbonization
• A cost-efficient and climate-compatible transition based on renewables
• Africa can become a self-sufficient green economy and an exporter of green fuels

## Graphical abstract

**Figure:**
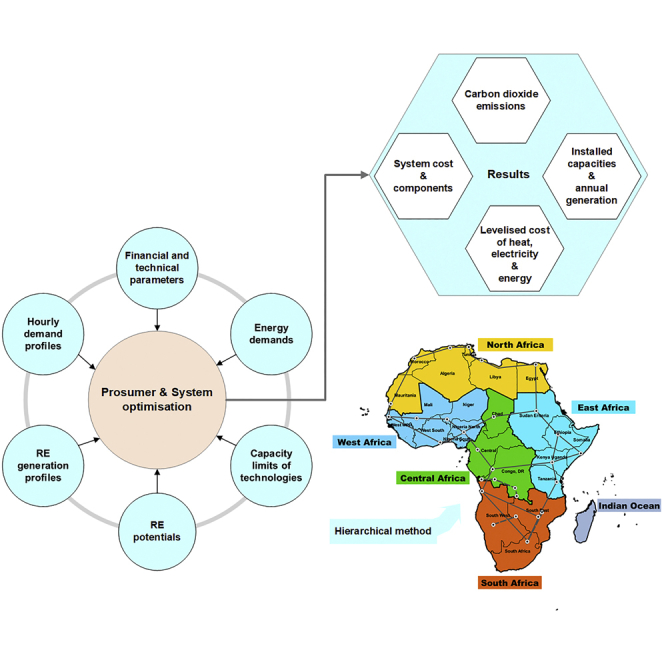

## Introduction

By choosing renewable energy (RE) sources over fossil fuels, Africa can create new industrial opportunities, new jobs, and experience greater socio-economic development while avoiding high-carbon pathways and the risk of being locked into a carbon-intensive development trajectory (Alova et al., 2021; Ram et al., 2020; Steckel et al., 2020). Notably, RE strategies are, to a large extent, linked to lower levels of energy-related emissions (Davis et al., 2018; Jin and Kim, 2018; Sovacool et al., 2020). The renewables climate mitigation hypothesis has been recently confirmed in the literature, with scenarios exemplifying the core competency of RE-based pathways in delivering a carbon-neutral power or energy system (Bogdanov et al., 2021a; Jacobson et al., 2019). Hansen et al. (2019) and Oyewo et al. (2021) review the state-of-the-art highly renewable energy system analyses in highlighting the lack of respective analyses in Africa. This, in turn, has contributed to misguiding the choice of energy investments, as Africa seeks to meet its fast-growing energy demand, scale-up industrialization, and achieve sustainable economic growth for the world’s fastest-growing population (Alova et al., 2021; Ireri and Shirley, 2021; Steckel et al., 2020). Investments in new fossil-fueled infrastructure, particularly coal, could transform many countries in Africa into major emitters or lock these countries into a dependency on fossil fuel energy (Ayompe et al., 2020; Guan et al., 2021; Steckel et al., 2020). One of the outcomes of the 26th Conference of the Parties (COP26) was to phase down coal by mid-century (United Nations Framework Convention on Climate, 2020). Africa could avoid the high-carbon choices that other developing countries, such as China, Indonesia, and India have pursued in the past (Guan et al., 2021). Whereas economic growth in these countries has managed to lift millions of people out of poverty, coal-based energy supply has evidently increased (Steckel et al., 2020). With the current unprecedented pace of technological improvements and continuous cost reduction of RE technologies (Bogdanov et al., 2021a; Jacobson et al., 2019), Africa can leapfrog to clean RE sources, thereby seizing opportunities before carbon lock-in emerges (Alova et al., 2021). In addition, the growing population in many African countries coupled with economic development will lead to a massive increase in energy demand and possibly CO_2_ emissions (Alova et al., 2021; Ayompe et al., 2020; Guan et al., 2021; Steckel et al., 2020). Historically, economic growth and fossil fuel use are closely linked (Steckel et al., 2020); however, such a trend could be avoided in Africa as energy services for all purposes could be provided in an economic and environmentally friendly way (Alova et al., 2021; Bogdanov et al., 2021a; Jacobson et al., 2019; IEA, 2019).

Recognizing the link between energy and socio-economic development, many African countries aim to increase investments in RE sources to curb the energy crisis and susceptibility to climate change (Oyewo et al., 2021; Guan et al., 2021; IEA, 2019). Despite access to abundant energy resources, Africa is still unable to utilize these enormous resources for optimal economic development (IEA, 2019). Consequently, the rate of access to electricity is still low at 56% leaving nearly 580 million people unelectrified (IEA, 2019, 2020). Furthermore, around 900 million people lack access to clean cooking mainly in sub-Saharan Africa (SSA) (IEA, 2019, 2020). The current electricity structure is dominated by fossil methane 39%, coal 30%, and hydropower 17% (IEA, 2021a). The total primary energy supply (TPES), though, is dominated by bioenergy at 45%, which is mainly used as an unsustainable fuel for cooking (IEA, 2021a). Crucially, substantial investments in renewables are required, both to decarbonize and provide modern energy services in Africa (Ireri and Shirley, 2021; IEA, 2019). During the last decade, RE investments in Africa were estimated at around 34 bUSD (Africa Land and Business, 2021); however, a higher level of investments is required over the coming decades to satisfy the growing demand (IEA, 2019). Given the current state of energy access in Africa, strong political will and stable economic development are essential to push access rates to 99% (IRENA, 2021a, 2021b). These factors are, to an evidently notable degree, associated with the rapid pace of electrification observed in China, India, and Bangladesh (IRENA, 2021a, 2021b), which is an important fact for African countries.

Endowed with substantial RE sources, African countries are excellent “greenfields” for large-scale RE deployment (Sterl, 2021a). Equally salient is the continuous decline in RE costs and their higher rates of positive learning (Junginger and Louwen, 2020; Vartiainen et al., 2020; Jaxa-Rozen and Trutnevyte, 2021; Schmidt et al., 2017). This, in turn, presents opportunities for low-cost energy supply in Africa (IEA, 2019; Barasa et al., 2018). Studies on possible future energy system options confirm the renewables climate mitigation strategy, and such an energy system can be delivered at a low cost with utmost societal welfare (Jin and Kim, 2018; Barasa et al., 2018; Bogdanov et al., 2021a; Jacobson et al., 2019; Oyewo et al., 2021). So far, Africa is one of the major regions in the world with limited research contextualizing energy pathways toward sustainable development through scenarios analyzing the contingencies and complexities of carbon-neutral and carbon-intensive pathway options. Furthermore, studies on RE transitions have mainly focused on the Global North, whereas comparative studies on fully RE transition and supportive RE policies for the Global South, especially for Africa, are rare (Hansen et al., 2019; Müller et al., 2020; Oyewo et al., 2021). This limited research creates less support for policymakers when designing future RE policy frameworks. Most RE policies in Africa have a predominant focus on the power sector, whereas comprehensive energy transition strategies for other energy-related sectors are either lacking or do not exist to enable comparable progress (Hansen et al., 2019; Oyewo et al., 2021; REN21, 2020). For instance, energy transition policies are grossly lacking in the transport sector (REN21, 2020). Aside from the potential carbonization that is anticipated to be driven by new investments in coal-fired capacity (Steckel et al., 2020), oil consumption in the transport sector has already been an important determinant of increasing carbonization in Africa (Steckel et al., 2020).

**Figure 1:**
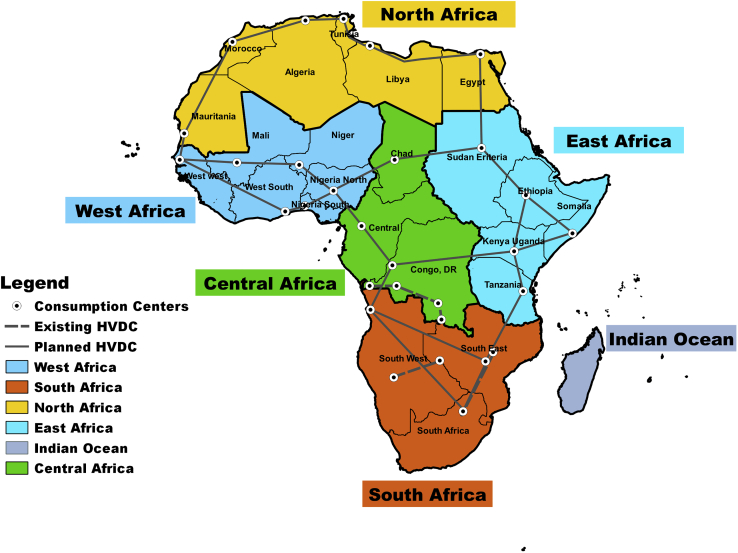
Overview of Africa structured in 6 macro regions and 24 regions In Figure 1, we show the spatial detail applied in this study. Owing to complexity in modeling, we applied the hierarchical method; thus, the optimization is carried out first at the macro-region level and secondly at the 24-region resolution, related to the STAR Methods and Table S1.

To bridge these research gaps, this study provides an in-depth analysis, contextualizing diverging fossil-fuel-dominated and renewables-based pathways toward sustainable development in Africa. This study, being the first of its kind, uses a novel technology-rich, multi-nodal, multi-sectoral, and multi-scenario techno-economic energy modeling tool to describe the scope of the pathways in high geo-spatial and full hourly resolution for Africa. For a comprehensive analysis, optimizations are carried-out in two hierarchical resolutions. In the first step, Africa was structured into 6 macro-regions and 24 regions in the second step, as shown in Figure 1, with a detailed structure presented in Table S1. In defining the transition pathways, four scenarios were examined under certain policy constraints (Table 1), namely, Best Policy Scenario (BPS), Best Policy scenario no carbon costs (BPSnoCC), Current Policy Scenario (CPS), and Current Policy scenario no carbon costs (CPSnoCC). It is worth pointing out that this study applies a different cost of capital (CoC) through the transition, based on the argument of Egli et al. (2019), as opposed the application of uniform CoC in leading reports (IEA, 2016; IRENA, 2018) and journal publication (Bogdanov et al., 2019, 2021a; Jacobson et al., 2019; Löffler et al., 2017; Teske et al., 2021).

**Table 1:** Scenario assumptions

Technology	BPS	BPSnoCC	CPS	CPSnoCC
Fischer–Tropsch liquids (FTL)	Yes	Yes	No	No
New fossil fuel and nuclear power plant	No new coal, oil, and nuclear; existing plants are phased out based on lifetime. Gas turbines allowed owing to possibilities to switch to sustainable fuels.	No new coal, oil, and nuclear; existing plants are phased out based on lifetime. Gas turbines allowed owing to possibilities to switch to sustainable fuels.	New coal, oil, gas, and nuclear plants can be built.	New coal, oil, gas, and nuclear plants can be built.
Hydropower and pumped hydro energy storage (PHES)	Across scenarios hydropower plants and pump hydro energy storage are refurbished every 35 years and never decommissioned.	Across scenarios hydropower plants and pump hydro energy storage are refurbished every 35 years and never decommissioned.	Across scenarios hydropower plants and pump hydro energy storage are refurbished every 35 years and never decommissioned.	Across scenarios hydropower plants and pump hydro energy storage are refurbished every 35 years and never decommissioned.
Policy	BPS	BPSnoCC	CPS	CPSnoCC
Carbon price	Yes	No	Yes	No
Cross-border trading	Yes	Yes	Yes	Yes

Showing some important constraints considered to better analyze the transition pathway options.

## Results

### Evolution of Africa’s power sector

**Figure 2:**
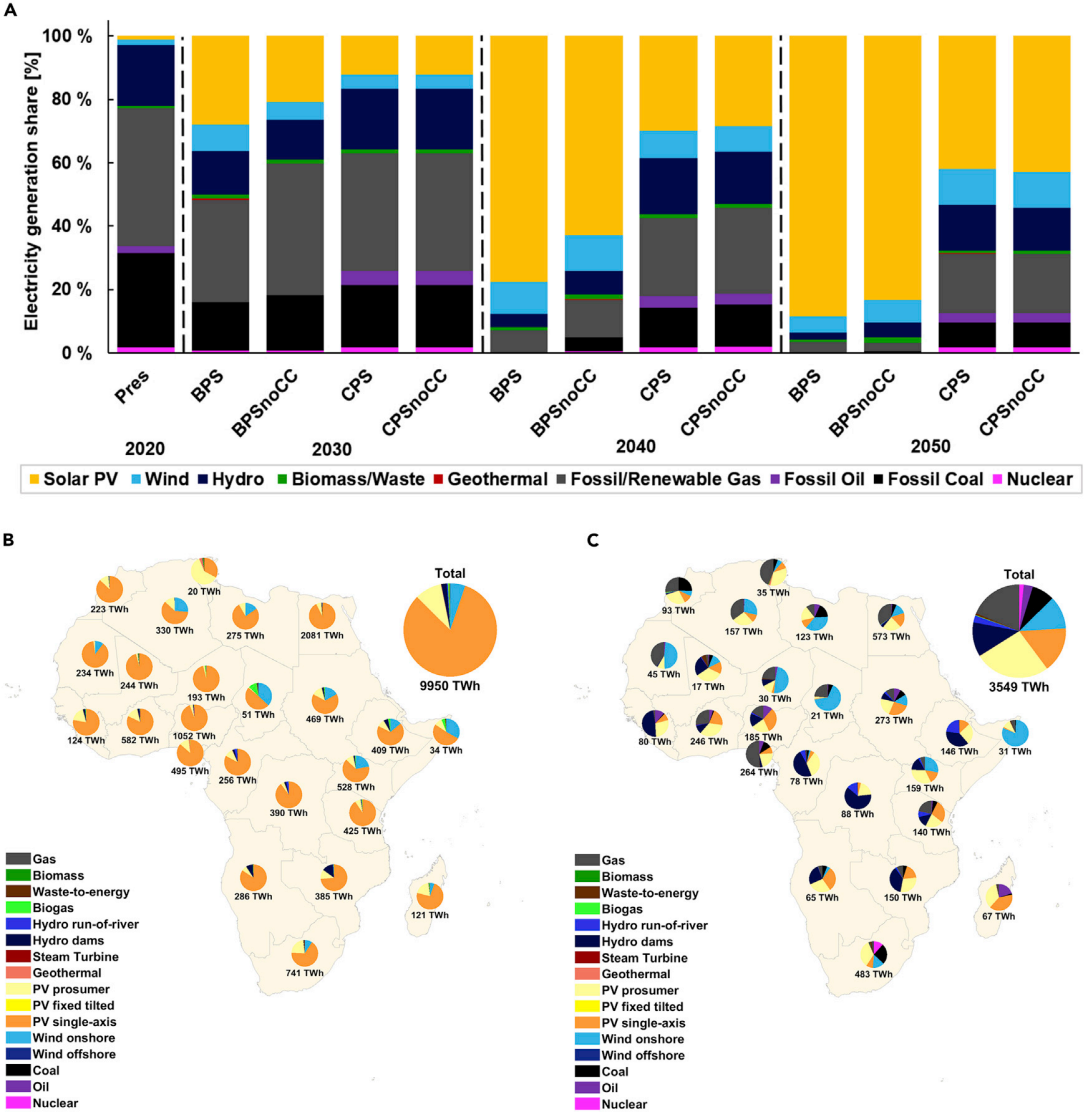
Electricity generation mix through the transition and regional electricity generation by 2050 (A) The electricity generation mix through the transition in 10-year intervals from 2020 to 2050 for all scenarios. (B and C) The regional electricity generation by 2050. In (A), we depict electricity in each node for the BPS by 2050 and the overall total for Africa. Similarly, (C) shows the electricity generation in each node for the CPS by 2050 and the overall capacity for Africa. Electricity generation in the Best Policy Scenarios is significantly higher than in the CPS pronounced, and sector integration leads to comprehensive electrification of the entire energy system demanding substantially more low-cost electricity. Related to Figures S6 and S7.

In order to prevent path-dependency and lock-in, the speed or temporal dynamics of the transition becomes a critical element of consideration. The need for rapid decarbonization of Africa’s power sector becomes apparent, as the current electricity supply structure is dominated by fossil fuels. These fuels, including oil, coal, and fossil methane account for about 80% (over 600 TWh) of the current generation mix, and 47–61% in 2030 (Figure 2A). The generation mix until 2030 shows the intention of many African countries to increase investments in fossil fuel plants, with the support of Chinese companies (Lema et al., 2021). Notably, the power sector in Africa is at a crossroads, if deep decarbonization of power generation does not occur from 2030 onwards then it could be too late to avoid carbon lock-in. As observed in the CPSs, these scenarios continue to favor an existing carbon-intensive generation mix through the transition, representing around 30% (1,000 TWh) of electricity supply in 2050. Conversely, beyond 2030, the BPSs illustrate a deep decarbonization of the power sector based on renewables.

The generation mix across scenarios is dominated by solar photovoltaics (PV) by 2050, representing 42–88% of the total electricity supply. The regional electricity generation outlook is depicted in Figures 2B and 2C, showing the relevance of solar PV across the continent, owing to excellent solar conditions and continuous cost reduction of PV technologies (Vartiainen et al., 2020; Victoria et al., 2021). Higher shares of solar generation in sunbelt countries are confirmed in literature (Bogdanov et al., 2021a; Jacobson et al., 2019; Breyer et a., 2018). Equally salient is the wind energy and hydropower contribution through the transition; however, these resources are not evenly distributed across the continent. Wind generation is around 340–520 TWh in 2050, supplied mainly by countries around the Sahara Desert, Horn of Africa, and some parts of Southern Africa. In 2050, hydropower generation is around 230–510 TWh, mainly supplied by countries in Central and East Africa. Other RE sources and gas turbines (running on e-fuels and biomethane and low full load hours), complement the solar-wind-hydropower generation in the BPSs. Conversely, coal, fossil methane, oil, and nuclear contribution remains relevant in the CPSs. Additional graphical results are provided in Figures S6 and S7.

### Circumventing contingencies and building operational flexibility

Beyond greening the grid with clean and cheap renewable electricity, consistency of supply is critical owing to high shares of variable RE in the system. Despite the continental coverage of this analysis, spanning a large geographic area, reliability of supply is crucial. Notably, the African generation mix depicts a tendency toward higher flexibility and complementarity while ensuring power sector resilience. More importantly, this analysis is performed on an hourly sequential temporal resolution (See Figures S8–S11), which allows an optimized use of resources and guarantees a sufficient level of detail in the model dispatch schedule. So far, most analyses for Africa are limited to time slices and annual energy balances; however, such a temporal resolution does not permit high levels of detail in an energy system (Hansen et al., 2019; Oyewo et al., 2021). Hereafter, we posit possible technical solutions including energy storage, electricity trade, sector integration, flexible generation, and demand response to enhance system flexibility.

#### Energy storage

**Figure 3:**
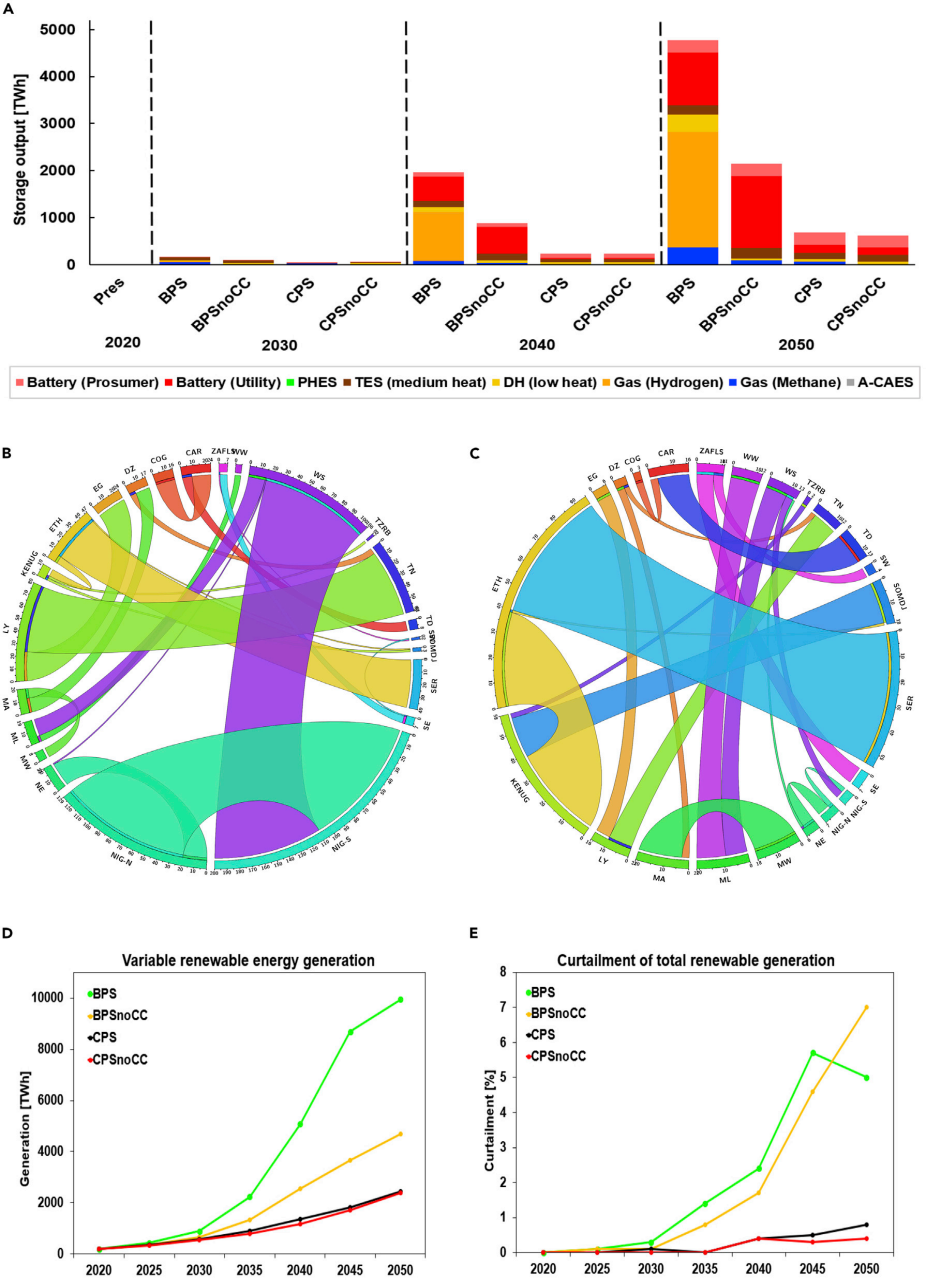
Overview of the storage output, variable renewable energy generation, curtailment, and electricity exchange across Africa (A) The storage output in 10-year interval from 2020 to 2050 by scenarios. (B) depicts the variable renewable energy generation. (C) shows the curtailment of total variable renewable energy generation. (D and E) The electricity exchange across the African regions – BPS (D) and CPS (E). Abbreviations of regions can be found in Table S1. Abbreviations: district heating (DH), pump hydro energy storage (PHES), thermal energy storage (TES), and adiabatic air compressed energy storage (A-CAES), related to Figures S12–S15.

Reliability of supply can be achieved using energy storage technologies, such as electrochemical, pumped hydro energy, heat, and e-fuel storage technologies that are applied in this analysis. As the share of RE grows in the system, the functionality of storage flexibility becomes expedient in providing a quick response to effectively manage variability in demand and supply (Figure 3A). In 2050, utility-scale and prosumer batteries supply the entire electricity storage output, ranging from 415–1800 TWh across scenarios. Battery storage technologies are anticipated to catalyze the power sector transition by enabling the penetration of low-cost VRE (BNEF, 2019; Kittner et al., 2017). Technological progress has driven the costs of the battery down by 90% in the 2010s, making their deployment increasingly cost-effective (Breyer et al., 2018; Peters et al., 2021). Critically, batteries can contribute to synthetic inertia (Oyewo et al., 2018), provide the back-up power, and support the PV prosumer sector and off-grid electrification (IRENA, 2019). Similarly, heat and e-fuels storage play a critical role by supplying 8–31% of heat sector demand in 2050. Thermal energy storage (TES) contributes 17–89% and power-to-gas (PtG) 11–83% of heat storage output across scenarios. PtG is mainly used in the carbon-neutral BPS scenario, whereas TES dominates in other scenarios. Additional results on storage are provided in Figures S12 and S13.

#### Transmission interconnection

Grid infrastructure provides further flexibility and reinforcement to the power system. Power transmission interconnectors make energy shifting possible, optimal use of RE resources widely dispersed across the continent and smoothens day-to-day variability. More importantly, grid interconnectors expand the geographic footprint of power systems. Few studies have analyzed the possibility and profitability of regional grid interconnection in Africa (Sridharan et al., 2019; Taliotis et al., 2014; Wu et al., 2017). Wu et al. (2017) conclude that with grid interconnection, low-cost renewable electricity can be traded within the East Africa power pool. The study further highlights the heterogeneity of RE resource across East Africa and that inter-country resource sharing can be facilitated with a power transmission grid Wu et al. (2017). Breyer et al. (2020) in their global analysis show that grid requirements in a fully RE system are lower in SSA compared with other regions owing to low seasonal variations and excellent solar resources across the region. In this study, volumes of electricity traded range from 123–563 TWh in 2050, representing 4–9% of the electricity demand. The necessary cross-border grid interconnection across the continent implies further costs as the capex in five-year intervals ranges from 36 to 230 b€. In the BPS, Ethiopia, west-south region, and Nigeria-North emerge as the major exporters, with 74, 85, and 96 TWh, respectively, in 2050. Most of the electricity from the west-south region and Nigeria-North is exported to Nigeria-South, thereby emerging as the highest importing region with 191 TWh (8% of the regional demand). In the CPS, Sudan-Eritrea, Ethiopia, and Mauritania dominate the exports with 65, 25, and 24 TWh, respectively, in 2050, and Kenya-Uganda, with 51 TWh, emerges as the main importing region. Substantial reductions in transmission and distribution losses are expected across the continent. Electricity trade across the regions is depicted in Figures 3B and 3C, additional results are provided in Figure S14.

#### Energy curtailment

Curtailed generation is another valuable operational strategy to improve the system flexibility. As depicted in Figures 3D and 3E, curtailment is often expected to increase with increases in VRE penetration (Carbajales-Dale et al., 2014; Cebulla et al., 2018; Heide et al., 2011; Jacobsen and Schröder, 2012; Solomon et al., 2019). VRE curtailment becomes a relevant and low-cost source of flexibility from 2030 onwards, predominantly in the BPSs. In the BPSs, curtailed generation is around 5–7% in 2050 as part of a least-cost solution and is about 0.4–0.8% in the CPSs. Curtailed generation is lower in the CPSs owing to substantial contributions from thermal power plants and hydropower in the system. Curtailment of renewable electricity is often debated as an unacceptable loss of green energy (Carbajales-Dale et al., 2014). This perspective could lead to overinvestment in grid interconnection and storage solutions (Jacobsen and Schröder, 2012). Crucially, other studies have shown that an optimally managed curtailment provides techno-economic benefits to the system (Bogdanov et al., 2021a; Solomon et al., 2019; Heide et al., 2011; Jacobsen and Schröder, 2012). Contingencies in BPSs can be managed with curtailments of VRE, similar findings exist for Europe (Cebulla et al., 2017) and wider geographical regions (Cebulla et al., 2018), showing curtailment as a part of least-cost optimization solution. Additional results on curtailment are provided in Figure S15.

#### Sector integration

Electricity as the new and dominating primary energy carrier provides opportunities for sector integration, thereby improving the overall system efficiency and flexibility (Bogdanov et al., 2021b; Brown et al., 2018; Eyre, 2021). Africa’s low-cost renewable electricity is a key driver for integrating previously separated end-use sectors, particularly in the carbon-neutral BPS scenario. Power-to-X (PtX) processes are further analyzed.

#### Power-to-heat

**Figure 4:**
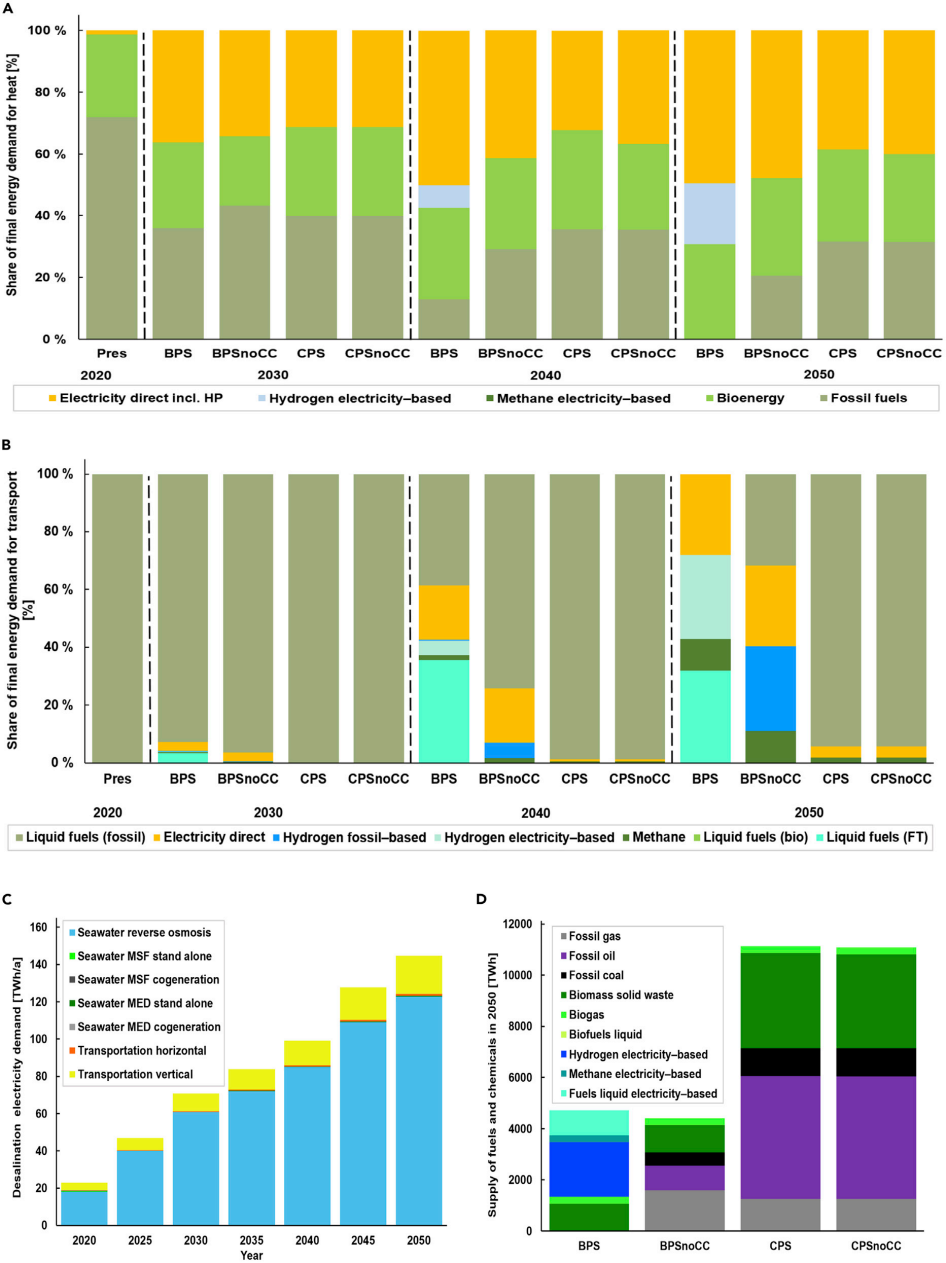
Structure of the final energy demand, desalination electricity demand, and supply of fuels (A and B) The share of energy demand in the heat (A) and transport (B) sectors. (C) shows the desalination electricity demand and (D) supply of fuels in 2050 (D). Abbreviations: multi-effect distillation (MED) and multi-stage flash (MSF), related to Figures S16–S19.

In 2050, heat demand accounts for 28–40% of the total final energy demand (TFED). Heat is mainly used for domestic hot water, industrial processes, and space heating, especially in the Republic of South Africa and North African countries. In 2050, direct electrification accounts for 39–50% of heat demand, bioenergy for 29–32%, fossil fuels for 0–32%, and e-fuels for 0–20% across scenarios (Figure 4A). In the carbon-neutral BPS scenario, direct electric heating including heat pumps (50%), followed by sustainable bioenergy (30%), and RE-based e-fuels (20%) cover the heat demand by 2050. The total installed heat generation capacity increases from nearly 300 GW in 2020 to around 440–495 GW in 2050. Similarly, heat generation across scenarios increases from about 988 TWh in 2020 to around 1,840–2,224 TWh in 2050.

#### Power-to-mobility

In the transport sector, TFED increases from around 1,870 TWh in 2020 to about 3,050 TWh in the BPSs and 4,695 TWh in the CPSs by 2050. Fuel shares in the transport sector across scenarios are depicted in Figure 4B. Direct electrification of the transportation demand ranges from 4 to 28%. Fuel shares are dominated by fossil fuels in the CPSs through the transition. In the BPSnoCC, transportation fuel is dominated by fossil fuels (32%), followed by gray hydrogen (29%), electricity (28%), and methane (11%) in 2050. Similar fuel shares are observed in the carbon-neutral BPS scenario, except that gray hydrogen is replaced by green hydrogen and fossil fuels are substituted by FTL fuels. The plausible reason for lower transportation demand in the BPSs compared with the CPSs is owing to the high rate of electrification of the transport modes, through direct and indirect means. The direct electrification of the transport sector is achieved mainly via road vehicles such as battery-electric and plug-in hybrids for light-, medium-, and heavy-duty vehicles, buses, 2,3 wheelers, and electrified railways (Khalili et al., 2019). Conversely, indirect transportation electrification is achieved via e-fuels such as hydrogen and FTL fuels (jet, gasoline, and diesel fuel).

#### Power-to-water

Seawater desalination demand is also coupled with low-cost renewable electricity. Africa’s desalination demand increases from around 14 mil m^3^ in 2020 to around 115 mil m^3^ in 2050, and, at the same time, the electricity demand increases from around 23 TWh/a in 2020 to 145 TWh/a in 2050, which is less than 2% of TFED across scenarios. The desalination electricity demand is shown in Figure 4C.

#### Power-to-fuel

Crucially, e-fuels are required for deep defossilization of the most difficult-to-abate energy services including long-distance aviation and marine transportation, and production of carbon-intensive materials such as cement and steel. Africa’s low-cost renewable electricity is used to produce e-fuels (Ueckerdt et al., 2021). Figure 4D depicts the supply of fuels in 2050. Pursuing a carbon-neutral pathway is less energy-intensive compared with a carbon-intensive pathway as observed in this study. Both direct and indirect electrification strategies account for lower energy demand in the carbon-neutral BPS scenario. Many African countries have shown excellent prerequisites for e-fuel production, and similar findings are established in the literature (Fasihi et al., 2017, 2021). Africa has a huge potential for green hydrogen production by utilizing its rich solar resources (Vartiainen et al., 2021). In recent years, African countries have been signing partnership agreements for local green hydrogen production (Bhagwat and Olczak, 2020; Dii, 2019; Matalucci, 2021). South Africa could repurpose Sasol to produce clean fuels, chemicals, and fertilizers, by transforming it from a coal- and gas-to-liquids to a power-to-liquids platform (Bischof-Niemz and Creamer, 2018). It is worth mentioning that e-fuel demand across Africa would require higher production capacity, which goes beyond Sasol’s production capacity. The installed capacities for fuel conversion technologies increase from 40 GW in 2030 to around 1640 GW in 2050. The massive demand for e-fuels is observed from 2040 onwards in the carbon-neutral pathway. Electrolyzers used for green hydrogen production dominate the shares of installed fuel conversion technologies beyond 2030s, accounting for 75% of total capacity followed by FTL synthesis plants (12%) in 20,500.

The heat required for e-fuel production in the CO_2_ direct air capture (DAC) phase is recovered from processes within the energy system, such as excess heat from FTL synthesis units. Heat utilized is around 587 TWhth in 2050, whereas the installed CO_2_ DAC and CO_2_ storage increases to nearly 400 MtCO_2_/a. Installed gas storage capacity for e-fuels increases to about 91 TWh.

Flexible generation and demand response. The modeling outcome illustrates that a fully decarbonized power system relying on low-cost solar and wind together with hydropower is achievable with a minor contribution from bioenergy and gas turbines powered by e-fuels. It is noteworthy that Africa’s supply side flexibility is also linked to the flexibility of dispatchable renewables, especially dammed hydropower (Sterl, 2021a). A sector-coupled energy system as observed in this study is closely linked to low-cost RE and valuable flexibility options in difficult-to-abate segments, mainly enabled by flexible electrolyzers for hydrogen production. Electrolyzers are an essential component of the e-fuel production chain and provide critical flexibility to the energy system in decoupling variable RE generation as synthesis units are operated near baseload via hydrogen storage. The total installed electrolyzer capacity is around 1,469 GW_el_ in 2050. Additional graphical results on this sector integration section are provided in Figures S16–S19 and S23–S25.

### Contextualizing diverging fossil-fuel dominated and renewable pathways

The divergence in pathways is contextualized in terms of costs and CO_2_ emissions.

#### Cost

**Figure 5:**
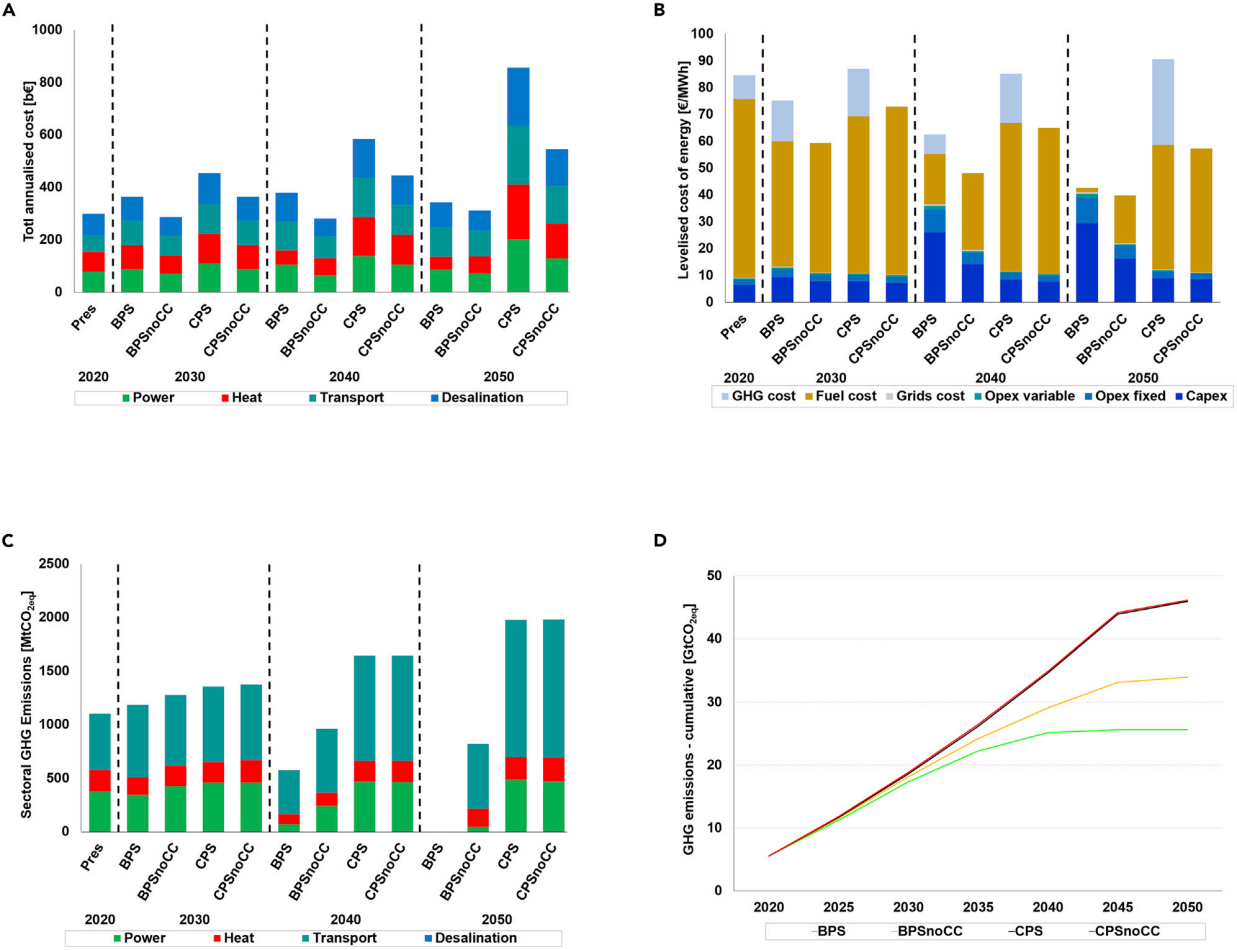
Total annualized cost, levelized cost of energy, CO_2_ emissions, and cumulative CO_2_ emissions for all scenarios (A) The total annualized cost of system by sectors including the power, heat, transport, and desalination. (B) depicts the levelized cost of energy through the transition. (C) illustrates the emissions trajectory by sector. (D) shows the cumulative emissions in five-year intervals from 2020 to 2050, related to Figures S20–S22.

Transitioning to a carbon-neutral or continuing with the carbon-intensive energy system as of today comes at a cost as depicted in Figure 5A. A carbon-neutral pathway is not only climate-compatible, but also highly cost-competitive with a carbon-intensive pathway. The cumulative costs of achieving a carbon-neutral energy system by 2050 in the BPS scenario are 50% lower than the cost of the carbon-intensive CPS scenario. Equally salient is that, excluding GHG emissions cost, the BPS scenarios are about 41% lower in cost than the CPS, indicating the pure market economics of renewables. The total annual cost in the carbon-neutral BPS scenario increased only by 14% in 2050 compared with the 2020 base level, whereas the TFED increased by 18%. On the contrary, costs increased by a factor of three in the carbon-intensive CPS scenario. The cost of energy is a crucial determining factor for evaluating the viability of the pathway options (Figure 5B). The levelized cost of energy obtained in the most ambitious carbon-neutral BPS scenario is around 43 €/MWh in 2050, about 26–53% lower than the CPSs. The cost structure is increasingly dominated by capital expenditures in the BPSs, and by fuel costs in the CPSs. In the years 2015–2020, spending on fuels accounts for two-thirds of Africa’s energy expenditure (IEA, 2021b). Technologically, the continuous cost-competitiveness of renewables, especially the availability of low-cost solar PV across Africa, makes the BPS scenarios more attractive. On the other hand, high costs in the CPS scenarios associate with the monumental and mounting cost of thermal plants and their operating costs. Crucially, thermal and nuclear power plants especially are prone to cost overruns and schedule spills (Sovacool et al., 2014a, 2014b) and could soon become stranded assets, more so after the COP26 phase-down agreement on coal (United Nations Framework Convention on Climate, 2020). In determining a cost-optimal energy system transition pathway, technical and financial assumptions of various technologies are adopted based on market development and insights from scientific literature, as presented in Table 2, Table 3, Table 4, Table 5, Table 6, Table 7, Table 8, Table 9 (Related to the Star Methods).

**Table 2:** Technical and financial assumptions of power technologies used in the energy transition from 2020 to 2050

PV optimally tilted	Capex	€/kW_el_	1,000	475	370	306	237	207	184	166	Vartiainen et al. (2020); ETIP-PV (2019); Mann et al. (2014)
	Opex fix	€/(kW_el_ a)	15	7.8	6.5	5.7	5	4.5	4.04	3.7	
	Opex var	€/(kWh_el_)	0	0	0	0	0	0	0	0	
	Lifetime	Years	30	30	35	35	35	40	40	40	
PV rooftop – residential	Capex	€/kW_el_	1,360	1,150	926	787	622	551	496	453	ETIP-PV (2019); Mann et al. (2014)
	Opex fix	€/(kW_el_ a)	20.4	9.1	7.7	6.7	5.9	5.3	4.8	4.4	
	Opex var	€/(kWh_el_)	0	0	0	0	0	0	0	0	
	Lifetime	years	30	30	35	35	35	40	40	40	
PV rooftop – commercial	Capex	€/kW_el_	1,360	758	598	502	393	345	308	280	ETIP-PV (2019); Mann et al. (2014)
	Opex fix	€/(kW_el_ a)	20.4	9.1	7.7	6.7	5.9	5.3	4.8	4.4	
	Opex var	€/(kWh_el_)	0	0	0	0	0	0	0	0	
	Lifetime	Years	30	30	35	35	35	40	40	40	
PV rooftop – industrial	Capex	€/kW_el_	1,360	563	437	362	281	245	217	197	ETIP-PV (2019); Mann et al. (2014)
	Opex fix	€/(kW_el_ a)	20.4	9.1	7.7	6.7	5.9	5.3	4.8	4.4	
	Opex var	€/(kWh_el_)	0	0	0	0	0	0	0	0	
	Lifetime	Years	30	30	35	35	35	40	40	40	
PV single-axis tracking	Capex	€/kW_el_	1,150	523	407	337	261	228	202	183	ETIP-PV (2019); Mann et al. (2014); Bolinger and Seel (2016)
	Opex fix	€/(kW_el_ a)	17.3	8.5	7.2	6.2	5.5	4.9	4.4	4.1	
	Opex var	€/(kWh_el_)	0	0	0	0	0	0	0	0	
	Lifetime	Years	30	30	35	35	35	40	40	40	
Wind onshore	Capex	€/kW_el_	1,250	1,150	1,060	1,000	965	940	915	900	Wiser et al. (2017); ETRI (2014); Lazard (2016)
	Opex fix	€/(kW_el_ a)	25	23	21.2	20	19.3	18.8	18.3	18	
	Opex var	€/(kWh_el_)	0	0	0	0	0	0	0	0	
	Lifetime	Years	25	25	25	25	25	25	25	25	
Hydro Reservoir/Dam	Capex	€/kW_el_	1,650	1,650	1,650	1,650	1,650	1,650	1,650	1,650	ETRI (2014)
	Opex fix	€/(kW_el_ a)	49.5	49.5	49.5	49.5	49.5	49.5	49.5	49.5	
	Opex var	€/(kWh_el_)	0.003	0.003	0.003	0.003	0.003	0.003	0.003	0.003	
	Lifetime	years	50	50	50	50	50	50	50	50	
Hydro Run-of-River	Capex	€/kW_el_	2,560	2,560	2,560	2,560	2,560	2,560	2,560	2,560	ETRI (2014)
	Opex fix	€/(kW_el_ a)	76.8	76.8	76.8	76.8	76.8	76.8	76.8	76.8	
	Opex var	€/(kWh_el_)	0.005	0.005	0.005	0.005	0.005	0.005	0.005	0.005	
	Lifetime	years	50	50	50	50	50	50	50	50	
Geothermal power	Capex	€/kW_el_	5,250	4,970	4,720	4,470	4,245	4,020	3,815	3,610	ETRI (2014); Sigfusson and Uihelein (2015)
	Opex fix	€/(kW_el_ a)	80	80	80	80	80	80	80	80	
	Opex var	€/(kWh_el_)	0	0	0	0	0	0	0	0	
	Lifetime	years	40	40	40	40	40	40	40	40	
Coal PP	Capex	€/(kW_el_)	1,600	1,600	1,600	1,600	1,600	1,600	1,600	1,600	ETRI (2014)
	Opex fix	€/(kW_el_ a)	20	20	20	20	20	20	20	20	
	Opex var	€/(kWh)	0.001	0.001	0.001	0.001	0.001	0.001	0.001	0.001	
	Lifetime	years	40	40	40	40	40	40	40	40	
Nuclear PP	Capex	4571	4,672	4,773	4,874	4,974	5,075	5,175	4,571	4,672	ETRI (2014)
	Opex fix	86,1	88	83	84,8	79,3	80,9	78,8	86,1	88	
	Opex var	00,025	0.0025	0.0025	0.0025	0.0025	0.0025	0.0025	0.0025	0.0025	
	Lifetime	40	40	40	40	40	40	40	40	40	
CCGT	Capex	€/(kW_el_)	775	775	775	775	775	775	775	775	IEA (2016)
	Opex fix	€/(kW_el_ a)	19.4	19.4	19.4	19.4	19.4	19.4	19.4	19.4	
	Opex var	€/(kWh_el_)	0.002	0.002	0.002	0.002	0.002	0.002	0.002	0.002	
	Lifetime	years	35	35	35	35	35	35	35	35	
OCGT	Capex	€/(kW_el_)	475	475	475	475	475	475	475	475	ETRI (2014)
	Opex fix	€/(kW_el_ a)	14.25	14.25	14.25	14.25	14.25	14.25	14.25	14.25	
	Opex var	€/(kWh_el_)	0.01	0.01	0.01	0.01	0.01	0.01	0.01	0.01	
	Lifetime	years	35	35	35	35	35	35	35	35	
Internal Combustion Generator	Capex	€/(kW_el_)	385	385	385	385	385	385	385	385	Lazard (2016)
	Opex fix	€/(kW_el_ a)	11.5	11.5	11.5	11.5	11.5	11.5	11.5	11.5	
	Opex var	€/(kWh_el_)	0.005	0.005	0.005	0.005	0.005	0.005	0.005	0.005	
	Lifetime	years	20	20	20	20	20	20	20	20	
Biomass PP	Capex	€/(kW_el_)	2,755	2,620	2,475	2,330	2,195	2,060	1,945	1,830	ETRI (2014)
	Opex fix	€/(kW_el_ a)	55.4	47.2	44.6	41.9	39.5	37.1	35	32.9	
	Opex var	€/(kWh_el_)	0.0037	0.0038	0.0038	0.0038	0.0038	0.0038	0.0038	0.0038	
	Lifetime	years	25	25	25	25	25	25	25	25	
Steam turbine (CSP)	Capex	€/(kWel)	1,000	968	946	923	902	880	860	840	Breyer et al. (2017)
	Opex fix	€/(kWel a)	20	19.4	18.9	18.5	18	17.6	17.2	16.8	
	Opex var	€/(kWhel)	0	0	0	0	0	0	0	0	
	Lifetime	years	25	25	25	25	30	30	30	30

**Table 3:** Technical and financial assumptions of heat technologies used in the energy transition from 2020 to 2050

CHP NG Heating	Capex	€/kW_el_	880	880	880	880	880	880	880	880	ETRI (2014)
	Opex fix	€/(kW_el_ a)	74.8	74.8	74.8	74.8	74.8	74.8	74.8	74.8	
	Opex var	€/(kWh_el_)	0.002	0.002	0.002	0.002	0.002	0.002	0.002	0.002	
	Lifetime	years	30	30	30	30	30	30	30	30	
CHP Oil Heating	Capex	€/kW_el_	880	880	880	880	880	880	880	880	ETRI (2014)
	Opex fix	€/(kW_el_ a)	74.8	74.8	74.8	74.8	74.8	74.8	74.8	74.8	
	Opex var	€/(kWh_el_)	0.002	0.002	0.002	0.002	0.002	0.002	0.002	0.002	
	Lifetime	years	30	30	30	30	30	30	30	30	
CHP Coal Heating	Capex	€/kW_el_	2030	2030	2030	2030	2030	2030	2030	2030	ETRI (2014)
	Opex fix	€/(kW_el_ a)	46.7	46.7	46.7	46.7	46.7	46.7	46.7	46.7	
	Opex var	€/(kWh_el_)	0.005	0.005	0.005	0.005	0.005	0.005	0.005	0.005	
	Lifetime	years	40	40	40	40	40	40	40	40	
CHP Biomass Heating	Capex	€/kW_el_	3500	3400	3300	3200	3125	3050	2975	2900	Danish Energy Agency and Energinet (2016)
	Opex fix	€/(kW_el_ a)	100.5	97.6	94.95	92.3	90.8	89.3	87.8	86.3	
	Opex var	€/(kWh_el_)	0.004	0.004	0.004	0.004	0.004	0.004	0.004	0.004	
	Lifetime	years	25	25	25	25	25	25	25	25	
CHP Biogas	Capex	€/kW_el_	503	429	400	370	340	326	311	296	Ram et al. (2019)
	Opex fix	€/(kW_el_ a)	20.1	17.2	16	14.8	13.6	13	12.4	11.8	
	Opex var	€/(kWh_el_)	0.001	0.001	0.001	0.001	0.001	0.001	0.001	0.001	
	Lifetime	years	30	30	30	30	30	30	30	30	
Waste incinerator	Capex	€/kW_el_	5,940	5,630	5,440	5,240	5,030	4,870	4,690	4,540	ETRI (2014)
	Opex fix	€/(kW_el_ a)	267.3	253.4	244.8	235.8	226.4	219.2	211.1	204.3	
	Opex var	€/(kWh_el_)	0.007	0.007	0.007	0.007	0.007	0.007	0.007	0.007	
	Lifetime	years	30	30	30	30	30	30	30	30	
Biogas digester	Capex	€/kW_th_	771	731	706	680	653	632	609	589	Urban et al. (2009)
	Opex fix	€/(kW_th_ a)	30.8	29.2	28.2	27.2	26.1	25.3	24.3	23.6	
	Opex var	€/(kWh_th_)	0	0	0	0	0	0	0	0	
	Lifetime	years	20	20	20	20	25	25	25	25	
Biogas upgrade	Capex	€/kW_th_	340	290	270	250	230	220	210	200	Urban et al. (2009)
	Opex fix	€/(kW_th_ a)	27.2	23.2	21.6	20	18.4	17.6	16.8	16	
	Opex var	€/(kWh_th_)	0	0	0	0	0	0	0	0	
	Lifetime	years	20	20	20	20	25	25	25	25	
CSP (solar field, parabolic trough)	Capex	€/kW_th_	438.3	344.5	303.6	274.7	251.1	230.2	211.9	196	Agora (2014); Svensson et al. (2004); Haysom et al. (2014)
	Opex fix	€/(kW_th_ a)	10.1	7.9	7	6.3	5.8	5.3	4.9	4.5	
	Opex var	€/(kWh_th_)	0	0	0	0	0	0	0	0	
	Lifetime	years	25	25	25	25	25	25	25	25	
Residential Solar Heat Collectors – space heating	Capex	€/kW_th_	1,286	1,214	1,179	1,143	1,071	1,000	929	857	Ram et al. (2019)
	Opex fix	€/(kW_th_ a)	14.8	14.8	14.8	14.8	14.8	14.8	14.8	14.8	
	Opex var	€/(kWh_th_)	0	0	0	0	0	0	0	0	
	Lifetime	years	20	25	25	30	30	30	30	30	
Residential Solar Heat Collectors – hot water	Capex	€/kW_th_	485	485	485	485	485	485	485	485	Ram et al. (2019)
	Opex fix	€/(kW_th_ a)	4.85	4.85	4.85	4.85	4.85	4.85	4.85	4.85	
	Opex var	€/(kWh_th_)	0	0	0	0	0	0	0	0	
	Lifetime	years	15	15	15	15	15	15	15	15	
DH Rod Heating	Capex	€/kW_th_	100	100	100	75	75	75	75	75	Ram et al. (2019)
	Opex fix	€/(kW_th_ a)	1.47	1.47	1.47	1.47	1.47	1.47	1.47	1.47	
	Opex var	€/(kWh_th_)	0.001	0.001	0.001	0.001	0.001	0.001	0.001	0.001	
	Lifetime	years	35	35	35	35	35	35	35	35	
DH Heat Pump	Capex	€/kW_th_	700	660	618	590	568	554	540	530	Ram et al. (2019)
	Opex fix	€/(kW_th_ a)	2	2	2	2	2	2	2	2	
	Opex var	€/(kWh_th_)	0.002	0.002	0.002	0.002	0.002	0.002	0.002	0.002	
	Lifetime	years	25	25	25	25	25	25	25	25	
DH Natural gas Heating	Capex	€/kW_th_	75	75	75	100	100	100	100	100	Ram et al. (2019)
	Opex fix	€/(kW_th_ a)	2.775	2.775	2.775	3.7	3.7	3.7	3.7	3.7	
	Opex var	€/(kWh_th_)	0.0002	0.0002	0.0002	0.0002	0.0002	0.0002	0.0002	0.0002	
	Lifetime	years	35	35	35	35	35	35	35	35	
DH Oil Heating	Capex	€/kW_th_	75	75	75	100	100	100	100	100	Ram et al. (2019)
	Opex fix	€/(kW_th_ a)	2.775	2.775	2.775	3.7	3.7	3.7	3.7	3.7	
	Opex var	€/(kWh_th_)	0.0002	0.0002	0.0002	0.0002	0.0002	0.0002	0.0002	0.0002	
	Lifetime	years	35	35	35	35	35	35	35	35	
DH Coal Heating	Capex	€/kW_th_	75	75	75	100	100	100	100	100	Ram et al. (2019)
	Opex fix	€/(kW_th_ a)	2.775	2.775	2.775	3.7	3.7	3.7	3.7	3.7	
	Opex var	€/(kWh_th_)	0.0002	0.0002	0.0002	0.0002	0.0002	0.0002	0.0002	0.0002	
	Lifetime	years	35	35	35	35	35	35	35	35	
DH Biomass Heating	Capex	€/kW_th_	75	75	75	100	100	100	100	100	Ram et al. (2019)
	Opex fix	€/(kW_th_ a)	2.8	2.8	2.8	3.7	3.7	3.7	3.7	3.7	
	Opex var	€/(kWh_th_)	0.0002	0.0002	0.0002	0.0002	0.0002	0.0002	0.0002	0.0002	
	Lifetime	years	35	35	35	35	35	35	35	35	
DH Geothermal heat	Capex	€/kW_th_	3,936	3,642	3,384	3,200	3,180	3,160	3,150	3,146	
	Opex fix	€/(kW_th_ a)	144	133	124	117	116	115	115	115	
	Opex var	€/(kWh_th_)	0	0	0	0	0	0	0	0	
	Lifetime	years	22	22	22	22	22	22	22	22	
Local Rod Heating	Capex	€/kW_th_	100	100	100	100	100	100	100	100	Ram et al. (2019)
	Opex fix	€/(kW_th_ a)	2	2	2	2	2	2	2	2	
	Opex var	€/(kWh_th_)	0.001	0.001	0.001	0.001	0.001	0.001	0.001	0.001	
	Lifetime	years	30	30	30	30	30	30	30	30	
Local Heat Pump	Capex	€/kW_th_	800	780	750	730	706	690	666	650	ETRI (2014)
	Opex fix	€/(kW_th_ a)	16	15.6	15	7.3	7.1	6.9	6.7	6.5	
	Opex var	€/(kWh_th_)	0	0	0	0	0	0	0	0	
	Lifetime	years	20	20	20	20	20	20	20	20	
Local Natural gas heating	Capex	€/kW_th_	800	800	800	800	800	800	800	800	Ram et al. (2019)
	Opex fix	€/(kW_th_ a)	27	27	27	27	27	27	27	27	
	Opex var	€/(kWh_th_)	0	0	0	0	0	0	0	0	
	Lifetime	years	22	22	22	22	22	22	22	22	
Local Oil Heating	Capex	€/kW_th_	440	440	440	440	440	440	440	440	ETRI (2014)
	Opex fix	€/(kW_th_ a)	18	18	18	18	18	18	18	18	
	Opex var	€/(kWh_th_)	0	0	0	0	0	0	0	0	
	Lifetime	years	20	20	20	20	20	20	20	20	
Local Coal Heating	Capex	€/kW_th_	500	500	500	500	500	500	500	500	Ram et al. (2019)
	Opex fix	€/(kW_th_ a)	10	10	10	10	10	10	10	10	
	Opex var	€/(kWh_th_)	0	0	0	0	0	0	0	0	
	Lifetime	years	15	15	15	15	15	15	15	15	
Local Biomass Heating	Capex	€/kW_th_	675	675	675	750	750	750	750	750	Ram et al. (2019)
	Opex fix	€/(kW_th_ a)	2	2	2	3	3	3	3	3	
	Opex var	€/(kWh_th_)	0	0	0	0	0	0	0	0	
	Lifetime	years	20	20	20	20	20	20	20	20	
Local Biogas Heating	Capex	€/kW_th_	800	800	800	800	800	800	800	800	Breyer et al. (2015)
	Opex fix	€/(kW_th_ a)	27	27	27	27	27	27	27	27	
	Opex var	€/(kWh_th_)	0	0	0	0	0	0	0	0	
	Lifetime	years	22	22	22	22	22	22	22	22

**Table 4:** Technical and financial assumptions of fuel conversion technologies used in the energy transition from 2020 to 2050

Technology		Units	2015	2020	2025	2030	2035	2040	2045	2050	Reference
Water electrolysis	Capex	€/kW_H2_	800	685	500	363	325	296	267	248	Breyer et al. (2015)
	Opex fix	€/(kW_H2_ a)	32	27	20	12.7	11.4	10.4	9.4	8.7	
	Opex var	€/(kWh_H2_)	0.001	0.001	0.001	0.001	0.001	0.001	0.001	0.001	
	Lifetime	years	30	30	30	30	30	30	30	30	
Methanation	Capex	€/kW_CH4_	547	502	368	278	247	226	204	190	Breyer et al. (2015)
	Opex fix	€/(kW_CH4_ a)	25.16	23.09	16.93	12.79	11.36	10.4	9.38	8.74	
	Opex var	€/(kWh_CH4_)	0.002	0.002	0.002	0.002	0.002	0.002	0.002	0.002	
	Lifetime	years	30	30	30	30	30	30	30	30	
CO_2_ direct air capture	Capex	€/t_CO2_ a	1,000	730	481	338	281	237	217	199	Fasihi et al. (2017, 2019)
	Opex fix	€/t_CO2_ a	40	29.2	19.2	13.5	11.2	9.5	8.7	8	
	Opex var	€/t_CO2_	0	0	0	0	0	0	0	0	
	Lifetime	years	20	20	30	25	30	30	30	30	
Fischer–Tropsch unit	Capex	€/kW,FT_Liq, output_	947	947	947	947	947	852.3	852.3	852.3	Fasihi et al. (2017)
	Opex fix	€/kW,FT_Liq, output_	28.41	28.41	28.41	28.41	28.41	25.57	25.57	25.57	
	Opex var	€/kW,FT_Liq, output_	0	0	0	0	0	0	0	0	
	Lifetime	years	30	30	30	30	30	30	30	30

**Table 5:** Technical and financial assumptions of storage technologies used in the energy transition from 2020 to 2050

Technology		Units	2015	2020	2025	2030	2035	2040	2045	2050	Reference
Battery storage utility-scale	Capex	€/kWh_el_	400	234	153	110	89	76	68	61	Vartiainen et al. (2020); ETIP-PV (2019); Giuliano et al. (2016)
	Opex fix	€/(kWh_el_ a)	24	3.3	2.6	2.2	2.1	1.9	1.8	1.7	
	Opex var	€/(kWh_el_)	0	0	0	0	0	0	0	0	
	Efficiency	%	91	91	92	93	94	95	95	95	
	Lifetime	years	15	20	20	20	20	20	20	20	
Battery interface utility-scale	Capex	€/kW_el_	200	117	76	55	44	37	33	30	Vartiainen et al. (2020); ETIP-PV (2019); Giuliano et al. (2016)
	Opex fix	€/(kW_el_ a)	0	1.5	1.3	1.1	1.01	0.9	0.9	0.8	
	Opex var	€/(kWh_el_)	0	0	0	0	0	0	0	0	
	Lifetime	years	15	20	20	20	20	20	20	20	
Battery PV prosumer – residential storage	Capex	€/kWh_el_	603	462	308	224	182	156	140	127	Vartiainen et al. (2020); ETIP-PV (2019);
	Opex fix	€/(kWh_el_ a)	36.2	5.08	4	3.4	3.09	2.8	2.8	2.5	
	Opex var	€/(kWh_el_)	0	0	0	0	0	0	0	0	
	Lifetime	years	15	20	20	20	20	20	20	20	
Battery PV prosumer – residential interface	Capex	€/kW_el_	302	231	153	112	90	76	68	62	Vartiainen et al. (2020); ETIP-PV (2019);
	Opex fix	€/(kW_el_ a)	0	2.5	2	1.7	1.5	1.4	1.4	1.2	
	Opex var	€/(kWh_el_)	0	0	0	0	0	0	0	0	
	Lifetime	years	15	20	20	20	20	20	20	20	
Battery PV prosumer – commercial storage	Capex	€/kWh_el_	513	366	240	175	141	121	108	98	Vartiainen et al. (2020); ETIP-PV (2019);
	Opex fix	€/(kWh_el_ a)	30.8	4.4	3.6	3	2.7	2.5	2.4	2.3	
	Opex var	€/(kWh_el_)	0	0	0	0	0	0	0	0	
	Efficiency	%	91	91	92	93	94	95	95	95	
	Lifetime	years	15	20	20	20	20	20	20	20	
Battery PV prosumer – commercial interface	Capex	€/kW_el_	256	183	119	88	70	59	53	48	Vartiainen et al. (2020); ETIP-PV (2019);
	Opex fix	€/(kW_el_ a)	0	2.2	1.8	1.5	1.3	1.2	1.2	1.1	
	Opex var	€/(kWh_el_)	0	0	0	0	0	0	0	0	
	Lifetime	years	15	20	20	20	20	20	20	20	
Battery PV prosumer – industrial storage	Capex	€/kWh_el_	435	278	181	131	105	90	80	72	Vartiainen et al. (2020); ETIP-PV (2019);
	Opex fix	€/(kWh_el_ a)	26.1	3.9	3.1	2.6	2.4	2.3	2.1	1.9	
	Opex var	€/(kWh_el_)	0	0	0	0	0	0	0	0	
	Efficiency	%	91	91	92	93	94	95	95	95	
	Lifetime	years	15	20	20	20	20	20	20	20	
Battery PV prosumer – industrial interface	Capex	€/kW_el_	218	139	90	66	52	44	39	35	Vartiainen et al. (2020); ETIP-PV (2019);
	Opex fix	€/(kW_el_ a)	0	2	1.5	1.3	1.2	1.1	1.01	1	
	Opex var	€/(kWh_el_)	0	0	0	0	0	0	0	0	
	Lifetime	years	15	20	20	20	20	20	20	20	
PHES	Capex	€/kWh_el_	7.7	7.7	7.7	7.7	7.7	7.7	7.7	7.7	ETRI (2014)
	Opex fix	€/(kWh_el_ a)	1.335	1.335	1.335	1.335	1.335	1.335	1.335	1.335	
	Opex var	€/(kWh_el_)	0	0	0	0	0	0	0	0	
	Efficiency	%	85	85	85	85	85	85	85	85	
	Lifetime	years	50	50	50	50	50	50	50	50	
PHES interface	Capex	€/kW_el_	650	650	650	650	650	650	650	650	ETRI (2014)
	Opex fix	€/(kW_el_ a)	0	0	0	0	0	0	0	0	
	Opex var	€/(kWh_el_)	0	0	0	0	0	0	0	0	
	Lifetime	years	50	50	50	50	50	50	50	50	
A-CAES	Capex	€/kWh_el_	75	75	65	58	54	51	47	44	ETRI (2014)
	Opex fix	€/(kWh_el_ a)	1.3	1.2	1	0.9	0.8	0.8	0.7	0.7	
	Opex var	€/(kWh_el_)	0	0	0	0	0	0	0	0	
	Efficiency	%	59	59	65	70	70	70	70	70	
	Lifetime	years	40	55	55	55	55	55	55	55	
A-CAES interface	Capex	€/kW_el_	540	540	540	540	540	540	540	540	ETRI (2014)
	Opex fix	€/(kW_el_ a)	17.5	17.5	17.5	17.5	17.5	17.5	17.5	17.5	
	Opex var	€/(kWh_el_)	0	0	0	0	0	0	0	0	
	Lifetime	years	40	55	55	55	55	55	55	55	
Gas Storage	Capex	€/kWh_el_	0.05	0.05	0.05	0.05	0.05	0.05	0.05	0.05	Michalski et al. (2017)
	Opex fix	€/(kWh_el_ a)	0.001	0.001	0.001	0.001	0.001	0.001	0.001	0.001	
	Opex var	€/(kWh_el_)	0	0	0	0	0	0	0	0	
	Efficiency	%	100	100	100	100	100	100	100	100	
	Lifetime	years	50	50	50	50	50	50	50	50	
Gas Storage interface	Capex	€/kW_th_	100	100	100	100	100	100	100	100	Vartiainen et al. (2020);
	Opex fix	€/(kW_th_ a)	4	4	4	4	4	4	4	4	
	Opex var	€/(kWh_th_)	0	0	0	0	0	0	0	0	
	Lifetime	years	15	15	15	15	15	15	15	15	
Hot Heat Storage	Capex	€/kWh_th_	50.8	41.8	32.7	26.8	23.3	21	19.3	17.5	Ram et al. (2019)
	Opex fix	€/(kWh_th_ a)	0.76	0.63	0.49	0.4	0.35	0.32	0.29	0.26	
	Opex var	€/(kWh_th_)	0	0	0	0	0	0	0	0	
	Efficiency	%	90	90	90	90	90	90	90	90	
	Lifetime	years	25	25	25	25	30	30	30	30	
Hot Heat Storage Interface	Capex	€/kWh_th_	0	0	0	0	0	0	0	0	Ram et al. (2019)
	Opex fix	€/(kWh_th_ a)	0	0	0	0	0	0	0	0	
	Opex var	€/(kWh_th_)	0	0	0	0	0	0	0	0	
	Lifetime	years	25	25	25	25	30	30	30	30	
District Heat Storage	Capex	€/kWh_th_	50	40	30	30	25	20	20	20	ETRI (2014)
	Opex fix	€/(kWh_th_ a)	0.8	0.6	0.5	0.5	0.4	0.3	0.3	0.3	
	Opex var	€/(kWh_th_)	0	0	0	0	0	0	0	0	
	Efficiency	%	90	90	90	90	90	90	90	90	
	Lifetime	years	25	25	25	25	30	30	30	30	
District Heat Storage Interface	Capex	€/kWh_th_	0	0	0	0	0	0	0	0	Ram et al. (2019)
	Opex fix	€/(kWh_th_ a)	0	0	0	0	0	0	0	0	
	Opex var	€/(kWh_th_)	0	0	0	0	0	0	0	0	
	Lifetime	years	25	25	25	25	30	30	30	30	
Hydrogen Storage	Capex	€/kWh_th_	0.24	0.24	0.24	0.24	0.24	0.24	0.24	0.24	Michalski et al. (2017)
	Opex fix	€/(kWh_th_ a)	0.01	0.01	0.01	0.01	0.01	0.01	0.01	0.01	
	Opex var	€/(kWh_th_)	0	0	0	0	0	0	0	0	
	Efficiency	%	100	100	100	100	100	100	100	100	
	Lifetime	years	15	15	15	15	15	15	15	15	
Hydrogen Storage interface	Capex	€/kW_th_	100	100	100	100	100	100	100	100	Michalski et al. (2017)
	Opex fix	€/(kW_th_ a)	4	4	4	4	4	4	4	4	
	Opex var	€/(kWh_th_)	0	0	0	0	0	0	0	0	
	Lifetime	years	15	15	15	15	15	15	15	15	
CO_2_ Storage	Capex	€/ton	142	142	142	142	142	142	142	142	Svensson et al. (2004)
	Opex fix	€/(ton a)	9.94	9.94	9.94	9.94	9.94	9.94	9.94	9.94	
	Opex var	€/ton	0	0	0	0	0	0	0	0	
	Efficiency	%	100	100	100	100	100	100	100	100	
	Lifetime	years	30	30	30	30	30	30	30	30	
CO_2_ Storage Interface	Capex	€/ton	0	0	0	0	0	0	0	0	Svensson et al. (2004)
	Opex fix	€/(ton a)	0	0	0	0	0	0	0	0	
	Opex var	€/ton	0	0	0	0	0	0	0	0

**Table 6:** Technical and financial assumptions of desalination and transmission technologies used in the energy transition from 2020 to 2050

Technology		Units	2015	2020	2025	2030	2035	2040	2045	2050	Reference
Reverse Osmosis Seawater Desalination	Capex	€/(m^3^/day)	1,150	960	835	725	630	550	480	415	Caldera et al. (2018)
	Opex fix	€/(m^3^/day a)	46	38.4	33.4	29	25.2	22	19.2	16.6	
	Consumption	kWh_th_/m^3^	0	0	0	0	0	0	0	0	
	Lifetime	years	25	25	30	30	30	30	30	30	
	Consumption	kWh_el_/m^3^	4.1	3.6	3.35	3.15	3	2.85	2.7	2.6	
Multi Stage Flash Standalone	Capex	€/(m^3^/day)	2,000	2,000	2,000	2,000	2,000	2,000	2,000	2,000	Caldera et al. (2018)
	Opex fix	€/(m^3^/day a)	100	100	100	100	100	100	100	100	
	Consumption	kWh_th_/m^3^	85	85	85	85	85	85	85	85	
	Lifetime	years	25	25	25	25	25	25	25	25	
	Consumption	kWh_el_/m^3^	2.5	2.5	2.5	2.5	2.5	2.5	2.5	2.5	
Multi Stage Flash Cogeneration	Capex	€/(m^3^/day)	3,069	3,069	3,069	3,069	3,069	3,069	3,069	3,069	
	Opex fix	€/(m^3^/day a)	121.4	121.4	121.4	121.4	121.4	121.4	121.4	121.4	
	Consumption	kWh_th_/m^3^	202.5	202.5	202.5	202.5	202.5	202.5	202.5	202.5	
	Lifetime	years	25	25	25	25	25	25	25	25	
	Consumption	kWh_el_/m^3^	2.5	2.5	2.5	2.5	2.5	2.5	2.5	2.5	
Multi Effect Distillation Standalone	Capex	€/(m^3^/day)	1438	1,200	1,044	906.3	787.5	687.5	600	518.8	Caldera et al. (2018)
	Opex fix	€/(m^3^/day a)	47.44	39.60	34.44	29.91	25.99	22.69	19.80	17.12	
	Consumption	kWh_th_/m^3^	68	51	44	38	32	28	28	28	
	Lifetime	years	25	25	25	25	25	25	25	25	
	Consumption	kWh_el_/m^3^	1.5	1.5	1.5	1.5	1.5	1.5	1.5	1.5	
Multi Effect Distillation Cogeneration Multi Effect Distillation Cogeneration	Capex	€/(m^3^/day)	2,150	2,150	2,150	2,150	2,150	2,150	2,150	2,150	
	Opex fix	€/(m^3^/day a)	61.69	61.69	61.69	61.69	61.69	61.69	61.69	68.81	
	Consumption	kWh_th_/m^3^	168	168	168	168	168	168	168	168	
	Lifetime	years	25	25	25	25	25	25	25	25	
	Consumption	kWh_el_/m^3^	1.5	1.5	1.5	1.5	1.5	1.5	1.5	1.5	
	Capex	€/(m^3^/day)	2,150	2,150	2,150	2,150	2,150	2,150	2,150	2,150	
Water Storage	Capex	€/m^3^	64.59	64.59	64.59	64.59	64.59	64.59	64.59	64.59	Caldera et al. (2018)
	Opex fix	€/(m^3^ a)	1.3	1.3	1.3	1.3	1.3	1.3	1.3	1.3	
	Opex var	€/m^3^	0	0	0	0	0	0	0	0	
	Lifetime	years	50	50	50	50	50	50	50	50	
High voltage alternating current transmission line (HVAC)	Capex	€/(kW km)	0.458	0.458	0.458	0.458	0.458	0.458	0.458	0.458	Bogdanov and Breyer (2016); Zickfeld et al. (2012)
	Opex fix	€/(kW km)	0.003	0.003	0.003	0.003	0.003	0.003	0.003	0.003	
	Opex var	€/(kWh km)	0	0	0	0	0	0	0	0	
	Lifetime	years	50	50	50	50	50	50	50	50	
	Capex	€/(kW km)	0.458	0.458	0.458	0.458	0.458	0.458	0.458	0.458	
High voltage direct current transmission line (HVDC)	Capex	€/(kW km)	1.044	1.044	1.044	1.044	1.044	1.044	1.044	1.044	Bogdanov and Breyer (2016); Zickfeld et al. (2012)
	Opex fix	€/(kW km)	0.003	0.003	0.003	0.003	0.003	0.003	0.003	0.003	
	Opex var	€/(kWh km)	0	0	0	0	0	0	0	0	
	Lifetime	years	50	50	50	50	50	50	50	50	
HVDC Converter pair	Capex	€/(kW)	180	180	180	180	180	180	180	180	Bogdanov and Breyer (2016); Zickfeld et al. (2012)
	Opex fix	€/(kW a)	1.8	1.8	1.8	1.8	1.8	1.8	1.8	1.8	
	Opex var	€/(kW km)	0	0	0	0	0	0	0	0	
	Lifetime	years	50	50	50	50	50	50	50	50

**Table 7:** Efficiency assumptions for HVDC and HVAC transmission lines (Zickfeld et al. 2012)

Component	Power losses
HVDC line	1.6%/1,000 km
HVDC converter pair	1.4%
HVAC	9.4%/1,000 km

**Table 8:** Financial assumptions for the fossil-nuclear fuel prices and GHG emission cost

Name of component	Unit	2020	2025	2030	2035	2040	2045	2050	References
Coal	[€/MWh_th_]	7.7	8.4	9.2	10.2	11.1	11.1	11.1	BNEF, 2015
Fuel oil	[€/MWh_th_]	35.2	39.8	44.4	43.9	43.5	43.5	43.5	BNEF, 2015
Fossil gas	[€/MWh_th_]	22.2	30	32.7	36.1	40.2	40.2	40.2	BNEF, 2015
Uranium	[€/MWh_th_]	2.6	2.6	2.6	2.6	2.6	2.6	2.6	IEA, 2015
GHG emissions	[€/t_CO2eq_]	28	52	61	68	75	100	150	BNEF, 2015

**GHG emissions by fuel type [t**_**CO**_**_2_**_**eq**_**/MWh**_**th**_**]**									

**Coal** (Edwards et al., 1996)			**Fuel oil** (Edwards et al., 1996)				**Fossil gas** (EPA, 2013)		
0.389			0.387				0.283		

The referenced values are all till 2040 and are kept stable for later periods (fuels) or are assumed to further increase for matching the Paris Agreement (GHG emissions).

**Table 9:** Energy self-discharge rates of storage technologies

Technology	Self-discharge [%/h]	References
Battery	0	Hoffmann (2014); IRENA (2017)
PHES	0	ETRI (2014)
A-CAES	0.1	ETRI (2014)
TES	0.2	Pleßmann et al. (2014)
Gas storage	0	Pleßmann et al. (2014)

#### Carbon dioxide emissions

Renewables do not only tend to display higher positive learning and low cost where deployed (Lazard, 2021; IRENA, 2021a, 2021b), but also associate significantly with lower levels of carbon emissions. Renewable electricity generation is not only cost-competitive, but also varies negatively with CO_2_ emissions compared with the fossil-dominated mix (Figure 5C). The CPS scenarios confirm the economic growth and carbonation nexus hypothesis, whereas the ambitious BPS scenario counteracts such pathways. Africa’s low-cost renewable electricity creates the opportunity to fully eliminate emissions related to the difficult-to-abate energy services. The carbon-intensive CPS pathways indicate that Africa would be burdened with about 46 GtCO_2_eq over the next three decades and with 34 GtCO_2_eq in the BPSnoCC. The ambitious BPS scenario heading toward carbon-neutrality results in remaining cumulative GHG emissions of only 26 GtCO_2_eq and down to zero over the next three decades (Figure 5D). Additional graphical results on cost and CO_2_ emissions are provided in Figures S20–S22.

## Discussion

This research investigates energy pathways toward renewable energy development for Africa, by contextualizing diverging carbon-neutral and fossil-dominated pathways. Results clearly demonstrate that a carbon-neutral strategy is not only climate-compatible, but also cost-competitive for Africa’s future energy system options. Such a pathway complies with the central aim of the Paris Agreement in comparison with carbon-intensive CPS scenarios. Hereafter, some key findings and co-benefits of the pathways are discussed.

### Africa shows excellent resource conditions for a renewable transition

Our analysis shows an evolving dominant solar PV share, and comparable findings are confirmed for sunbelt regions (Breyer et al., 2020; Bogdanov et al., 2021a; Jacobson et al., 2019; Mensah et al., 2021; Oyewo et al., 2019, 2021). Across the scenarios, the solar PV share increases as the most cost-efficient technology. Importantly, climate change is expected to affect the reliability on the energy supply and resilience of energy systems (Yalew et al., 2020). Gernaat et al. (2021) analyzed the impacts of climate change on renewable energy supply and conclude that impacts on solar power are minimal whereas impacts on hydropower and wind energy are uncertain across the world. Emodi et al. (2019) conclude that solar PV systems are more resilient to climate change when compared with other renewables. Equally important is the socio-political realm of electricity infrastructure as this aspect is often overlooked by planners in Africa. Recently, the Grand Ethiopian Renaissance Dam (GERD) has attracted political tension between Ethiopia, Sudan, and Egypt (Sterl et al., 2021b). Political stability has a vital implication for the cross-border trade of hydroelectricity in Africa (Trotter et al., 2018). Beyond the political tensions related to hydropower development in Africa, they are susceptible to climate change (IEA, 2019) and are often over time or budget (IEA, 2021b; Sovacool et al., 2014a, 2014b). At the same time, large hydropower projects are falling out of favor with international donors. Hydropower is at a crossroads where capacity is yet to be built (Peters et al., 2021). Therefore, a solar-driven energy system is not only cost-competitive, but also climate-compatible and decreases network dependence on politically unstable countries. The BPS energy systems are solar driven but also complemented substantially by wind and hydropower with minor contributions from other RE sources including sustainable bioenergy and geothermal energy. From a resource perspective, Africa has both the energy and land resources to pursue clean and cost-competitive energy pathways. However, technologies harnessing RE sources are characterized by lower power density than fossil fuels (van de Ven et al., 2021). Consequently, only 0.026–0.11% of Africa’s land is required for solar PV and 0.037–0.063% for wind, based on an assumed specified capacity density, which is 100 and 8.4 MW/km^2^ and for optimally tilted PV and onshore wind respectively (Bogdanov and Breyer, 2016; Vartiainen et al., 2020). The required land can be used for Agriculture and PV and wind farms (Schindele et al., 2020), or in co-location with hydropower reservoirs (Farfan and Breyer, 2018; Sanchez et al., 2021). Moreover, non-arable and non-used, barren, or waste lands with excellent solar and wind resources that are close to the ocean can be used for green fuel production.

### Stability and flexibility in power systems is achievable

There are no technical showstoppers to achieving a power system with high shares of renewables in Africa. Importantly, Africa is the world region with the least power grid requirements owing to stable solar conditions all through the year. This, in turn, allows for an accelerated transition and faster decentralized VRE ramping, mainly through hybrid PV-battery systems. The possibility of PV-storage hybrid systems dominating the future energy system is also reported in recent literature (Creutzig et al., 2017; Haegel et al., 2019; Kittner et al., 2017; Lu et al., 2021). The PV-battery configuration will be driven by innovation and continuous cost reduction of batteries and PV technologies (Junginger and Louwen, 2020; Jaxa-Rozen and Trutnevyte, 2021; Kittner et al., 2017; Schmidt et al., 2017; Vartiainen et al., 2020; Victoria et al., 2021). Crucially, Africa’s generation mix illustrates an inclination toward higher flexibility and complementarity. This synergetic operation implies that hydropower and wind energy can balance the wet season (monsoon or rainy periods) when solar power, the main source of electricity, is limited. Additional flexibility in the energy system is provided by energy storage, electric grid networks, curtailed generation, and sector coupling. It is worth mentioning that large-scale build-out of transmission is required across Africa. Crucially, regional cooperation in Africa will expand the geographic footprint of power systems, unlock flexibility, and can be helpful for countries with very small loads, where economies of scale are hard to obtain. Evidently, the flexibility need will continue to increase in systems dominated by VRE sources, as observed in the BPS scenarios, and decrease in the CPSs owing to high shares of thermal plants, hydropower, and minor contribution from nuclear power systems.

### Comparison of key parameters across scenarios

The pathways are compared through the lens of the key pillars of a decarbonization strategy as presented in Table 10. Under this framework, this study demonstrates that renewables positively associate with low-cost and lower levels of CO_2_ emissions for Africa by 2050.

**Table 10:** Differences in key parameter across scenarios in 2050

Parameter	Units	BPS	BPSnoCC	CPS	CPSnoCC
RE share in PE	[%]	100	64	45	44
Electricity demand	[PWh_el_]	8.6	4.4	3.5	3.4
Electricity generation	[PWh_el_]	10.3	4.8	3.6	3.5
Installed power capacity	[TW]	5.1	2.4	1.2	1.2
Primary energy demand	[PWh]	10.7	8.5	13.2	13.1
Primary energy demand per capita	[MWh/person]	4.2	3.3	5.3	5.2
Hydrogen	[type]	Green	Gray		
e-fuel	[PWh_th_]	3.4			
Bioenergy	[PWh_th_]	1.3	1.3	4.0	4.0
Efficiency gains of total final energy demand	[%]	43	55	30	30

Showing the comparison among scenarios through the lens of key parameters of a decarbonization strategy. Related to Figures S23–S25.

This study consolidates insights on a significant reduction in primary energy demand (PED) that arises by progressing from today’s inefficient combustion-driven system that is powered by fossil fuels to a renewables-based, sector-coupled, and electricity-based energy system (Bogdanov et al., 2021a, 2021b). The carbon-neutral BPS pathway delivers an energy system with noticeable increases in efficiency through direct and indirect electrification. Moreover, this pathway envisages behavioral change, as PED per capita declines with the adoption of electrified devices. Sustainable biomass is mainly used in the heat sector and the remaining demand of difficult-to-abate segments is supplied by e-fuels. It is worth mentioning that fossil carbon capture utilization and storage technologies are not included in this analysis. Renewable electricity-based CO_2_ DAC carbon capture and utilization, however, is a core element. Most importantly, Africa can achieve a carbon-neutral energy system over the next three decades at a low cost without violating the principles of sustainability.

### Investments and new industrial opportunities

Transitioning to renewables or continuing with the fossil-fuel-dominated system in Africa by 2050 hinges on a substantial expansion in investments. The BPSs are lower in total costs than the CPSs. Developing economies including Africa are set to contribute to the bulk of emissions growth in the coming decades if the right choices in energy investments and strong regional and international actions are not taken (Bischof-Niemz and Creamer, 2018). Africa’s rapid population and economic growth could spur increasing levels of CO_2_ emissions in the continent (Ayompe et al., 2020). Investments in renewables and sector-coupling technologies as depicted in this study are required to drive Africa’s sustainable development in the coming decades. However, access to the required capital and higher CoC poses significant challenges for Africa. Thus, efforts to reduce risks and an improved ecosystem for investing in RE projects will attract capital. Mobilizing capital on a large scale in Africa will require the involvement of Development Financial Institutions (DFIs), private developers, and large climate finance commitments from advanced economies. More importantly, deep defossilization of the energy system could create new industrial opportunities in producing e-fuels and hydrogen-rich chemicals in Africa. This, in turn, will stimulate job creation on different value chain levels and avoid or reduce energy imports substantially. Therefore, Africa could become a self-sufficient green hydrogen economy and an exporter of green fuels.

### Improved energy service could be achieved

Transitioning to renewables for all purposes could create access to modern energy services and improve the livelihood of hundreds of millions of Africans. Access to clean cooking and heating remains a challenge in Africa, about 70% of the population depends on polluting fuels and technologies, especially biomass, which results in health and environmental problems (IEA, 2019, 2020). Tackling climate change could imply shifting away from unsustainable bioenergy for cooking and fossil fuels for lighting to zero-emission innovations. This study highlights the significant role of solar PV in Africa’s future energy system. Evidently, solar PV systems possess excellent features suitable for both on-grid and off-grid electrification. More importantly for Africa is access to clean cooking. This study anticipates access to improved energy services for domestic purposes such as cooking and heating. Such access would eliminate indoor air pollution exposure from solid fuels and other inefficient stoves in poorly ventilated homes, especially for poor households owing to their vulnerability to energy poverty (Goldemberg et al., 2018; WHO, 2014). In sum, tackling energy injustice means providing energy access based on sound principles of sustainability, affordability, respect, and equity. Because Africa is a key driver of global energy demand growth, achieving global Sustainable Development Goals requires Africa’s success.

### Conclusion and policy *implications*

Given the future energy system options presented in this study, efficient and competent institutions are required to drive Africa’s RE transition. Stable and strong institutional support will be needed soon to promote both energy access and defossilization of Africa’s energy system. Critically, to facilitate this level of transition in Africa, strong regional cooperation is needed to unlock economies of scale and develop a well-defined renewable energy roadmap over the coming decades. For instance, the agreement of the Africa Continental Trade Area could facilitate the creation of a pan-African and global green economy. More importantly, social institutions are required to harness the young workforce in different value chains of the transition including manufacturing, construction, and installation, operation and maintenance, decommissioning, and green fuel production. Several institutional actors are expected to drive the implementation of renewable energy strategies in Africa. The future energy system based on renewables will require strengthening systemic innovation in Africa. Innovative approaches will be required to fully maximize the potential of the future energy system including sector coupling, strengthening and modernizing the grid, green hydrogen, and related PtX options, and combining two or more technologies such as hybrid PV-hydropower reservoir systems.

Transitioning to a carbon-neutral energy system requires progressive policy and political will at all levels of governance in Africa. Political initiatives are required both to drive and deliver a sustainable energy transition. As may be obvious, African governments are not yet essentially prioritizing climate change measures owing to continued investments in fossil fuel plants. Therefore, breaking the trend toward carbon-intensive investments requires global mitigation efforts. Evidently, Africa is not a major emitter; however, it is vulnerable to the threats of climate change. In this perspective, the wealthy and historical primary polluters need to provide substantial support to developing regions including Africa to circumvent the hurdles of climate change already manifesting in the continent. International negotiation at its peak is missing proper representation, and Africa must be fully involved in global discussions. Contrasting the political intention for large-scale hydropower development in Africa, an optimal mix of renewable energy sources will increase reliance and improve system flexibility. Such a mix could cater to several disagreements related to water for hydropower development among neighboring countries, as recently observed between Egypt, Sudan, and Ethiopia. Crucially, energy policy in Africa could have solar PV at its core, grid interconnection, storage, and e-fuel production such as hydrogen to integrate higher shares of RE in the energy system. Beyond renewable energy expansion in Africa, policies are required to disincentivize further investments in fossil-fuel-related projects at all value chain steps. Such policy measures could introduce carbon costs as applied in this study. Notably, our findings show that Africa can leapfrog carbonization by using local low-cost renewable electricity and green hydrogen. Africa could therefore become a self-sufficient renewable energy and green hydrogen economy and an exporter of green fuels, thereby creating new industrial opportunities and jobs. Crucially, local capacities for employment in the transition need to be built, which is currently lacking in the continent. Finally, our analysis shows that a “true-zero emission” pathway is achievable in Africa, as it could be possible for all developing economies.

### Limitations of the study

The energy system is large and designed in a complex structure with a technology-rich portfolio. The optimization is performed in high temporal and spatial resolution to capture the variability of RE technologies and improve system reliability. This results in millions of variables which increases the computational time. In this regard, optimizations are carried out in two hierarchical resolutions to reduce the computational time. It is worth mentioning that the applied cost assumptions are based on market development and scientific literature; however, they may be too optimistic in the African context, in particular for the near future. We have applied varying CoC in each time step but uniformly across Africa. Furthermore, one might need to modify the applied CoC to a country-specific situation and cost assumptions to the African context. This study has added insights into how green technologies can deliver, in combination, deep and cross-sectoral decarbonization and defossilization, and into the flexible role of grid interconnection and storage technologies. However, policies and finance to drive such transition are largely lacking in Africa.

## STAR★Methods

### Key resources table

**Table:** 

RESOURCES	SOURCE	IDENTIFIER
**Input data**		

Technical and financial assumptions, electricity price for different segments, energy demand across the sectors, and renewable energy resource potential.	Table 2, Table 3, Table 4, Table 5, Table 6, Table 7, Table 8, Table 9, Table 10	

**Model**		

Main equations and model description	Method details	
LUT Energy System Transition Model	(Bogdanov et al., 2021a)	https://doi.org/10.1016/j.energy.2021.120467

**Software and algorithms**		

MOSEK version 7	Mosek ApS	https://www.mosek.com/downloads/
MATLAB R2016a	The MathWorks, Inc.	http://allpcworld.com/matlab-free-download/

### Resource availability

#### Lead contact

Further information and requests for resources should be directed to and will be fulfilled by the Lead Contact, Ayobami Solomon Oyewo (solomon.oyewo@lut.fi).

#### Material availability

This study did not generate new materials.

### Method details

#### Model overview

The African energy system is analyzed based on sound techno-economic principles and relies firmly on engineering logic for designing a low-cost energy system. An in-depth techno-economic analysis of Africa’s possible future energy system orientation as illustrated in this study presents the implication of alternative policy choices. The techno-economic modeling tool applied in this study, the LUT Energy System Transition Model (LUT-ESTM), is a linear optimisation tool with technology-rich, multi-nodal, multi-sectoral, multi-scenario and energy transition features, and operates on an hourly sequential temporal resolution. A detailed overview of the model equations and descriptions is described below based on Bogdanov et al. (2021a;2021b) and improved and validated for the case of Ethiopia in Oyewo et al. (2021).

The energy system configuration covers the energy conversion, residential, commercial, industry and transport sectors. These sectors are classified into power, heat, transport, and desalination sectors. The system configuration is optimised by minimizing the annual system cost, in 5-year time steps, from 2020 until 2050, in hourly sequential time resolution to ensure demand and supply balance at each hour. The modeling is carried out in two steps. In the first step, Africa was structure into 6 macro region and 24 regions. In the second step (Figure 1), this hierarchical modeling approach description is provided in Bogdanov et al. (2022). In addition, the energy system analysis accounts for prosumer PV and battery storage capacities and the transition of individual heating systems for space and water heating. Detailed description and model equations of the used LUT Energy System Transition Model are provided in Bogdanov et al. (2021a;2021b).

#### Applied technologies

In the power sector, electricity can be generated by solar PV, onshore wind, hydropower, geothermal, bioenergy, open cycle gas turbines (OCGT), combined cycle gas turbines (CCGT), oil, coal, and nuclear power plants and combined heat and power (CHP) units including oil, biomass, coal, and gas CHPs. In the heat sector, heat can be supplied by concentrated solar thermal power (CSP) parabolic fields, geothermal district heaters, individual solar thermal water heaters, bioenergy (solid biomass, biogas district heat, and individual boilers), district and individual boilers using oil and gas, and coal-based district heating. Electricity can be stored in batteries and pumped hydro energy storage (PHES), adiabatic compressed air energy storage (A-CAES) and gas storage for hydrogen and methane. Heat can be stored in thermal energy storage (TES) technologies. Sector coupling technologies includes power-to-gas (H2, CH4), H2-to-X (CH4, FTL fuels), CO2 DAC, seawater desalination (reverse osmosis, multi-effect distillation), steam turbines, electrical heaters, district and individual scale heat pumps, and direct electrical heaters. These technologies enable energy conversion from one sector into valuable products for another sector thereby increasing the entire system flexibility, efficiency, and decrease total costs of the system. The model schematic is shown in Figure S2.

The transportation demand is derived for the modes: road, rail, maritime transport, and aviation for passenger and freight transport. The road segment is subdivided into passenger LDV, passenger 2W/3W, passenger bus, freight MDV, and freight HDV. The other transportation modes are comprised of demand for freight and passengers. The demand is estimated in passenger kilometers (p-km) for passenger transport and in tonne kilometers (t-km) for freight transport.

The transportation demand is converted into energy demand by assuming an energy transition from current fuels to fully sustainable fuels by 2050, whereas the following principal fuel types are considered and visualized in Figure S3:

• Road: electricity, hydrogen, liquid fuels.
• Rail: electricity, liquid fuels
• Maritime: electricity, hydrogen, methane, liquid fuels
• Aviation: electricity, hydrogen, liquid fuels

The energy system can take fossil fuels, as long as it is allowed or affordable, and it can convert biomass to biofuels and produce renewable electricity based synthetic fuels to use all fuels in the sectors Power, Heat or Transport.

Currently hydrogen, methane and liquid hydrocarbons production units are integrated in the model.

Methane can be produced from biogas after its purification/upgrading, then this biomethane can be used in the gas system, the share of biogas, which can be upgraded is limited by the urbanization level of the region but cannot exceed 70% even if the urbanization level is higher. Second option is synthetic natural gas (SNG) – methane produced with methanation reactors from hydrogen and carbon dioxide. The whole Power-to-Gas (PtG) system includes water electrolysis reactors (assumptions are based on alkaline technology), producing hydrogen from water, CO2 direct air capturing (DAC) units, collecting CO2 and water from ambient air, and methanation units. Water electrolysers and DAC units consume power from the system in order to produce H2 and CO2, methanation units converts it to synthetic CH4.

Liquid hydrocarbons can be produced from biomass by biorefineries or can be synthesized from H2 and CO2 using the Fischer-Tropsch process. PtG with gas storage and gas turbines can be part of storage for the Power sector.

Fossil fuel refineries are not included in the model and existing capacities of refineries are assumed sufficient to satisfy local consumption of fossil fuels.

### The fuel conversion process adopted to produce sustainable fuels (see Figure S4)

The fuel shares of the transportation modes in the road segment are based directly or indirectly on levelised cost of mobility (LCOM) considerations for newly sold vehicles that change the stock of vehicles according to the lifetime composition of the existing stock. Vehicle stock and overall demand data are then linked to specific energy demand values to calculate demand of fuels and electricity for the transport sector. A more detailed description of the methodology is provided in Breyer et al. (2019).

The desalination demand is estimated for regions with water stress greater than 40% and is a function of the water stress and total water demand for a specific year. The water stress we refer to is explained in more detail in Caldera et al. (2016). The total water demand is the sum of the projected demand from the municipal, industrial and agricultural sectors. Irrigated agriculture accounts for 70% of the global water withdrawals. However, the average global irrigation efficiency is estimated to be as low as 33% and experience a maximum relative growth rate of 0.3% per annum. In Caldera and Breyer (2019), a scenario is presented where the irrigation efficiencies are increased using a maximum relative growth rate of 1% per annum. The irrigation efficiency growth rate per annum varies with water stress, based on a logistic expression. It is assumed that irrigation sites with water stress higher than 80% have a maximum growth rate of 1% per annum. The improved irrigation efficiency results in reduction in water demand, water stress and consequently desalination demand for a given year. This methodology, the data and assumptions used, to project the desalination demand from 2020 to 2050, are discussed in Caldera et al. (2021) and Caldera and Breyer. (2020). Therefore, the desalination demand presented in the report addresses the demands of the municipal, industrial and agricultural sector with improved irrigation efficiency.

#### Main equations

##### Model – Target function

The target of the system optimisation is the minimization of the total annual cost of the integrated system (or a sector if only a sector is optimized), calculated as the sum of the annual costs of installed capacities of the different technologies, costs of energy and products generation and production ramping. This target function includes annual costs of the Power, Heat, Transportation, Industrial (Industrial fuels production, Desalination and CO_2_ removal) sectors. The target function of the applied energy model for minimizing annual costs is presented in (Equation 1) and comprises all hours of a year using the abbreviations: sub-regions (*r, reg*), generation, storage and transmission technologies (*t, tech*), capital expenditures for technology *t* (*CAPEX*_*t*_), capital recovery factor for technology *t* (*crf*_*t*_), fixed operational expenditures for technology *t* (*OPEXfix*_*t*_), variable operational expenditures technology *t* (*OPEXvar*_*t*_), installed capacity in the region *r* of technology *t* (*instCap*_*t,r*_), annual generation by technology *t* in region *r* (*E*_*gen,t,r*_), cost of ramping of technology *t* (*rampCost*_*t*_) and sum of power ramping values during the year for the technology *t* in the region *r* (*totRamp*_*t,r*_).

(Equation 1) $$\min\left(\sum_{r=1}^{reg}\sum_{t=1}^{tech}\left(CAPEX_{t}\cdot crf_{t}+OPEXfix_{t}\right)\cdot instCap_{t,r}+OPEXvar_{t}\cdot E_{gen,t,r}+rampCost_{t}\cdot totRamp_{t,r}\right)$$

The power prosumers and individual heating users’ system is realized in an independent sub-model with a slightly different target function. The prosumers system is optimized for each sub-region independently, even if the sub-region is connected to neighbors inside the area. The target function includes annual costs of the prosumers power generation and storage, and heating equipment, the cost of electricity required from the distribution grid and the cost of fuels required for boilers, income of electricity feed-in to the distribution grid is deducted from the total annual cost.

The target function of the applied energy model for minimizing annual costs is presented in (Equation 2) and comprises all hours of a year using the abbreviations: generation and storage technologies (*t, tech*), capital expenditures for technology *t* (*CAPEX*_*t*_), capital recovery factor for technology *t* (*crf*_*t*_), fixed operational expenditures for technology *t* (*OPEXfix*_*t*_), variable operational expenditures technology *t* (*OPEXvar*_*t*_), installed capacity of technology *t* (*instCap*_*t*_), annual generation by technology *t* (*E*_*gen,t*_), retail price of electricity (*elCost*), feed-in price of electricity (*elFeedIn*), annual amount of electricity required from the grid ($E_{grid}$), annual amount of electricity fed-in to the grid ($E_{curt}$).

(Equation 2) $$\min\left(\sum_{t=1}^{tech}\left(CAPEX_{t}\cdot crf_{t}+OPEXfix_{t}\right)\cdot instCap_{t}+OPEXvar_{t}\cdot E_{gen,t}+elCost\cdot E_{grid}+elFeedIn\cdot E_{curt}\right)$$

##### Energy balance constraints

The main constraint for the Power sector optimisation is the matching of the power generation and demand for every hour of the applied year as shown in (Equation 3), for every hour of the year the total generation within a sub-region and electricity import covers the local electricity demand.

(Equation 3) $$\forall \text{h}\in \left[1,8760\right]\sum_{t}^{tech}E_{gen,t}+\sum_{r}^{reg}E_{imp,r}+\sum_{t}^{stor}E_{stor,disch}=E_{demand}+\sum_{r}^{reg}E_{exp,r}+\sum_{t}^{stor}E_{stor,ch}+E_{curt}+E_{other}$$

(Equation 3) describes constraints for the energy flows of a sub-region. Abbreviations: hours (*h*), technology (*t*), all modeled power generation technologies (*tech*), sub-region (*r*), all sub-regions (*reg*), electricity generation (*E*_*gen*_), electricity import (*E*_*imp*_), storage technologies (*stor*), electricity from discharging storage (*E*_*stor, disch*_), electricity demand (*E*_*demand*_), electricity exported (*E*_*exp*_), electricity for charging storage (*E*_*stor,ch*_), electricity consumed by other sectors (Heat, Transport, Desalination, Industrial fuels production, CO_2_ removal) (*E*_*other*_), curtailed excess energy (*E*_*curt*_). The energy loss in the high voltage direct current (HVDC) and alternating current (HVAC) transmission grids and energy storage technologies are considered in storage discharge and grid import value calculations.

The Heat sector energy balance is defined by 3 equations: for industrial high temperature heat demand, for industrial high and medium temperature heat demand, all centralised heat demand: all industrial demand (high, medium, and low temperature heat) and centralized space and water heating demand. High temperature heat can be only generated by fuel-based boilers Equation (Equation 4). Medium temperature heat can be also generated by electrical heating and can be stored in high temperature heat storage and used to produce electricity with steam turbines (Equation 5). Low temperature heat can be also provided by heat pumps, electric heating rods and waste heat from other technologies (Equation 6).

(Equation 4) $$\forall \text{h}\in \left[1,8760\right]\;\sum_{t}^{techHH}E_{gen,t}=E_{demandHH}$$

(Equation 5) $$\forall \text{h}\in \left[1,8760\right]\;\sum_{t}^{techHH}E_{gen,t}+\sum_{t}^{techMH}E_{gen,t}+E_{stor,disch}+=E_{demandHH}+E_{demandMH}+E_{stor,ch}+E_{other}$$

(Equation 6) $$\forall \text{h}\in \left[1,8760\right]\;\sum_{t}^{tech}E_{gen,t}+\sum_{t}^{stor}E_{stor,disch}=E_{demand}+\sum_{t}^{stor}E_{stor,ch}+E_{curt}+E_{other}$$

Abbreviations: hours (h), technology (t), high temperature heat generation technologies (techHH), medium temperature heat generation technologies (techMH), all heat generation technologies (tech), industrial high temperature heat demand ($E_{demandHH}$), industrial medium temperature heat demand ($E_{demandMH})$, total centralized heat demand, including industrial, and space heating and water heating demand ($E_{demand}$).

Power and Heat sector constraints for prosumers have some minor differences. Prosumers can buy electricity from the electricity distribution companies (Equation 7). Heating of prosumers based on individual heaters includes fuel, RE and electricity-based heaters, but there is no individual heat storage option (Equation 8).

(Equation 7) $$\forall \text{h}\in \left[1,8760\right]\;\sum_{t}^{tech}E_{gen,t}+\sum_{t}^{stor}E_{stor,disch}=E_{demand}-E_{grid}+\sum_{t}^{stor}E_{stor,ch}+E_{curt}+E_{other}$$

(Equation 8) $$\forall \text{h}\in \left[1,8760\right]\;\overset{tech}{\underset{t}{\sum }}E_{gen,t}=E_{demand}+E_{curt}$$

Abbreviations: hours (h), technology (t), all modeled power generation technologies (tech), energy generated (Egen), storage technologies (stor), energy from discharging storage (Estor, disch), energy demand based generation technology (EgenRE), capacity factor of the technology (CFgenRE), installed capacity in the region of the technology (instCapgenRE).

The fuel-based power and heat generation defined by the optimal installed capacity for this technology (Equation 10), availability factor for this technology (Equation 11), this technology used fuel available (Equation 12), and efficiency of the technology (Equation 13).

(Equation 10) $$\forall \text{h}\in \left[1,8760\right]\;E_{genFU,h}<=instCap_{genFU}$$

(Equation 11) $$\sum_{h}^{8760}E_{genFU,h}\leq \;8760\cdot AF_{genFU,h}\cdot instCap_{genFU}$$

(Equation 12) $$\;\sum_{h}^{8760}FU_{genFU,h}\leq \;totalFU_{genFU}$$

(Equation 13) $$\forall \text{h}\in \left[1,8760\right]\;E_{genFU,h}=\;FU_{genFU,h}\cdot eff_{genFU}$$

Abbreviations: hour (h), fuel-based generation technology (genFU), energy generated by fuel based generation technology (EgenFU), installed capacity in the region of the technology (instCapgenFU), availability factor of the technology (AFgenFU), fuel consumption for the hour h (FUgenFU,h), annual fuel consumption for the hour h (totalFUgenFU,h), energy conversion efficiency for technology (effgenFU).

For all technologies, capacity is calculated in output units, for cogeneration the capacity is given in electrical units.

For some types of fuel: Municipal wastes, Industrial biomass wastes, Biogas – all available fuel must be consumed for sustainability reasons. Biogas inflow in the system is constant and biogas can be stored only for 48 h.

##### Capacity definition through transition periods and system cost calculation

The active capacity existing in the system is defined at each of the steps for each of the regions based on the data of the capacity installed at previous steps and the lifetime for the given technology at given commissioning year as presented in (Equation 14)

(Equation 14) $$\forall \text{t}\in \left[tech\right]\;existingCap_{t,year}=\sum_{y=1960}^{year-5}newCap_{t,y}\cdot \left(\left(y+N_{t,y}\right)>year\right)$$

Abbreviations: year (y, year), generation and storage technologies (t, tech), existing active capacity for technology t at the modeled year (existingCapt, year), new built capacity for technology t at the previous year y (newCapt,y), lifetime of the capacity of technology t built at year y (Nt,y).

Then, the model optimisation results in the optimal regional capacity of the technologies in the given year, which defines the new built capacity needed by the system as defined in (Equation 15).

(Equation 15) $$\forall \text{t}\in \left[tech\right]\;newCap_{t,year}=instCap_{t,year}-existingCap_{t,year}$$

Abbreviations: modeling year (year), generation and storage technologies (t, tech), existing active capacity for technology t at the modeled year (existingCapt, year), new built capacity for technology t at the previous year y (newCapt,y), total capacity for technology t at given year year as defined by the model optimisation (instCap t,year).

The energy costs calculations in the post processing phase are based on a historical approach, where the annualised cost of the system considers the financial assumptions in the periods when these capacities were built, unlike the approach used in the optimisation and described in the (Equation 1). For the variable opex calculations, the energy output of technologies is split accordingly to the capacity age structure as defined in (Equation 16).

(Equation 16) $$\forall \text{t}\in \left[tech\right],\forall \text{y}\in \left[1960\ldots year\right]\;E_{genSplit,t,y}=E_{gen,t,year}\cdot \left(newCap_{t,y}\cdot \left(\left(y+N_{t,y}\right)>year\right)\right)/instCap_{t,year}$$

Abbreviations: modeling year (year), all years from 1960 (y), generation and storage technologies (t, tech), annual generation by technology t by capacity built at year y (EgenSplit,t,y), new built capacity for technology t built at year y (newCapt,y), annual generation by technology t defined by the model for the modeling year year (Egen,t,year), total capacity for technology t at given year year as defined by the model optimisation (instCap t), lifetime of the capacity of technology t built at year y (Nt,y).

Then the annualised cost of the system at the given year is calculated accordingly to the (Equation 17).

(Equation 17) $$annualCost_{year}=\;\sum_{r=1}^{reg}\sum_{t=1}^{tech}\sum_{y=1960}^{year}\left(CAPEX_{r,t,y}\cdot crf_{t,y}+OPEXfix_{r,t,y}\right)\cdot \left((newCap_{r,t,y}\cdot \left(\left(y+N_{t,y}\right)>year\right)\right)+OPEXvar_{r,t,y}\cdot E_{genSplit,r,t,y}+rampCost_{t}\cdot totRamp_{r,t}$$

This historical cost calculation approach is used for other cost calculations including LCOE and split of LCOE in sub-categories.

Abbreviations: modeling year (year), all years from 1960 (y), generation and storage technologies (t, tech), capital expenditures for technology t in region r and year y (CAPEXr,t,y), capital recovery factor for technology t (crft), fixed operational expenditures for technology t in region r and year y (OPEXfixr,t,y), variable operational expenditures technology t in region r and year y (OPEXvarr,t,y), new built capacity for technology t built in region r at year y (newCapr,t,y), lifetime of the capacity of technology t built at year y (Nt,y), annual generation by technology t in region r at year year by capacity built at year y (EgenSplit,r,t,y), cost of ramping of technology t (rampCostt), sum of power ramping values during the year for the technology t in the region r (totRampr,t).

##### Power and heat storage

Storage technologies are described with energy storage capacity and storage interface capacity. Energy storage capacity limits the maximum state of charge (SoC) of the storage, the amount of energy stored (Equation 18), while the storage interface capacity limits the maximum power of charge and discharge Equations 19 and 20. Energy balance constraint for storage technologies is given in (Equation 21).

(Equation 18) $$\forall \text{h}\in \left[1,8760\right]\;SoC_{stor,h}<=instCapEn_{stor}$$

(Equation 19) $$\forall \text{h}\in \left[1,8760\right]\;E_{stor,ch,h}<=instCapInt_{stor}$$

(Equation 20) $$\forall \text{h}\in \left[1,8760\right]\;E_{stor,disch,h}<=instCapInt_{stor}$$

(Equation 21) $$\forall \text{h}\in \left[1,8760\right]\;SoC_{stor,h}=\;SoC_{stor,h-1\;}\cdot selfDisch_{stor\;}+E_{stor,ch,h}\cdot eff_{stor,ch}-E_{stor,disch,h}/eff_{stor,disch}$$

Abbreviations: hour (h), storage technology (stor), storage state of charge for an hour h (SoCstor,h), installed energy capacity of the storage (instCapEnstor), installed power capacity of the storage (instCapIntstor), charging energy of the storage for an hour h (Estor,ch,h), discharging energy of the storage for an hour h (Estor, disch,h), hourly Self-discharge of the storage (selfDischstor), charge efficiency (effstor,ch), discharge efficiency (effstor, disch).

##### Power transmission

Power transmission is presented by HVDC and HVAC grids. Each line of the grid is represented in the model as 2 unidirectional lines: import and export line, capacity of each line is equal to the total capacity of the line, as shown in (Equation 22). Hourly export/import energy for a sub-region is calculated as sum of all import lines multiplied by this line transmission efficiency minus sum of all export line energy flows, as shown in (Equation 23). The efficiency of energy transmission with HVDC lines depends on the distance and AC/DC converters pair efficiency, as shown in (Equation 24), efficiency of energy transmission with HVAC line depends only on distance, as shown in (Equation 25). For both HVDC and HVAC the distance related losses are calculated in a simplified way.

(Equation 22) $$\forall \text{h}\in \left[1,8760\right]\;line_{import,h}\leq instCap_{line}\;;\;line_{export,h}<=instCap_{line}$$

(Equation 23) $$\forall \text{h}\in \left[1,8760\right]\;E_{exp/imp,h}=\sum_{l}^{lines}line_{import,l,h}\cdot eff_{l}-\sum_{l}^{lines}line_{export,l,h}$$

(Equation 24) $$eff_{l}=eff_{CS}\cdot \left(1-distance\cdot EffLoss\right)$$

(Equation 25) $$eff_{l}=1-distance\cdot EffLoss$$

Abbreviations: hour (h), line (L), energy flow through the power line (line), installed capacity of the power line (instCapline), exported/imported energy for the region for an hour h (Eexp/imp,h), total energy import efficiency (effl), converter pair efficiency (effCS), charge length of the line (distance), energy loss in the line (EffLoss).

##### Desalination

In case that in the region exists desalinated water demand, the system has to provide the demanded amount of water every hour. Water storage at supply side provides flexibility to the system. Desalination units are located on the seashore, and they can optimize the work in order to decrease the total system cost. Water demand and water storage balance is described in Equations 26 and 27.

Water desalination units produce the water and store it in the water storage, desalinated water production is limited by optimal capacities of enabled desalination plants and storage technologies (Equations 28 and 29). Power, heat and gas consumption for desalination units’ operation as shown in Equations (Equation 30), (Equation 31), (Equation 32) are included in the power, heat and gas balance equations on demand side. The water pumping electricity demand according to (Equation 33) and cost is calculated based on the pumping capacity of the system, hourly water demand, weighted average length and head of the piping system.

(Equation 26) $$\forall \text{h}\in \left[1,8760\right]\;\sum_{t}^{tech}W_{des,t,h}+W_{stor,disch,h}-W_{stor,ch,h}=W_{demand,h}$$

(Equation 27) $$\forall \text{h}\in \left[1,8760\right]\;SoC_{stor,h}=\;SoC_{stor,h-1\;}+W_{stor,ch,h}-W_{stor,disch,h}$$

(Equation 28) $$\forall \text{h}\in \left[1,8760\right]\;W_{des,t,h}\leq instCapDes_{t}$$

(Equation 29) $$\forall \text{h}\in \left[1,8760\right]\;SoC_{stor,h}\leq \;instCapStor_{\;}$$

(Equation 30) $$\forall \text{h}\in \left[1,8760\right]\;E_{heat,h}=\sum_{t}^{tech}W_{des,t,h}\cdot heatCons_{t}$$

(Equation 31) $$\forall \text{h}\in \left[1,8760\right]\;E_{el,h}=\sum_{t}^{tech}W_{des,t,h}\cdot elCons_{t}-\sum_{t}^{tech}W_{des,t,h}\cdot elProd_{t}$$

(Equation 32) $$\forall \text{h}\in \left[1,8760\right]\;E_{gas,h}=\sum_{t}^{tech}W_{des,t,h}\cdot gasCons_{t}$$

(Equation 33) $$\forall \text{h}\in \left[1,8760\right]\;E_{elPump,h}=\sum_{t}^{tech}W_{des,t,h}\times \left(elCons_{vPump}\cdot alt+elCons_{hPump}\cdot dist\right)\;$$

Abbreviations: hour (h), desalination technology (t), desalinated water (Wdes), water storage discharge (Wstor, disch), water storage charge (Wstor,ch), water demand (Wdemand), installed desalination technology capacity (instCapDes), desalination heat demand (Eheat), desalination electricity demand (Eel), desalination gas demand (Egas), desalination heat consumption (heatCons), desalination electricity consumption (elCons), desalination electricity production (elProd), desalination gas consumption (gasCons), water pumping electricity demand (EelPump), horizontal water pumping electricity consumption (elConshPump), vertical water pumping electricity consumption (elConsvPump), pumping distance (dist), pumping altitude difference (alt), water storage state of charge h (SoCstor), installed capacity of the water storage (instCapStor).

##### CO_2_ removal

The energy system can capture additional amounts of CO_2_ from atmosphere for the permanent storage, this CO_2_ captured by DAC and stored in CO_2_ buffer storage. The system will balance hourly DAC and CO_2_ buffer operation in order to balance hourly CO_2_ removal demand.

##### Transport and fuel production

Transportation demand is expressed in transportation demand in (metric) ton kilometers (t-km) for freight and passenger kilometers (p-km) for passengers. Power and fuels consumption for a given mix of transportation means operation are included in the Power, Heat and gas (H_2_, CH_4_) balance equations on the demand side.

Instead of presenting equations, this section is described in a qualitative way, as to how the transport sector is modeled.

The transportation demand for freight and passengers is allocated to the transportation modes road, rail, marine and aviation. For the case of the road mode this transportation demand is further allocated to the segments for light duty vehicles, buses and 2/3-wheeler for passengers and medium and heavy-duty vehicles for freight. The thus allocated transportation demand is then allocated to powertrain types to cover the allocated demand. The powertrains are BEV, FCEV, PHEV and ICE for the road mode. The used fuel types for rail are electricity and liquid hydrocarbons, for marine are electricity, LNG, LH_2_ and liquid hydrocarbons and for aviation the fuel types are electricity, LH_2_ and liquid hydrocarbons.

All such allocated demand to the respective powertrains and fuel types is then linked to efficiency assumptions of the respective conversion technologies for the transition period from 2020 to 2050. This then leads to final energy demand for the fuels: electricity, liquid hydrocarbons, LH_2_ and LNG. These fuels are then supplied by respective fuel production which transitions from the current mix of fuels to a fully sustainable fuel mix through the transition. All the applied assumptions are summarised in Khalili et al. (2019). Furthermore, the fuel shares of the transportation modes in the road segment are based directly or indirectly on levelised cost of mobility (LCOM) considerations for newly sold vehicles, which change the stock of vehicles according to the lifetime composition of the existing stock. Vehicle stock and overall demand data are then linked to specific energy demand values to calculate demand of fuels and electricity for the transport sector.

##### Fuel production for transport

The fuel production comprises electricity and methane, which is also described for the power sector. Methane is converted by a liquefaction unit to LNG. Hydrogen as a fuel is also partly described for the power sector, however for the transport sector in addition steam methane reforming (SMR) is part of the available components. Marine and aviation also requires LH_2_, which is represented in the model by a respective liquefaction unit. Liquid hydrocarbons represent fuels such as diesel, gasoline, jet fuel, biofuels, and synthetic fuels. Biofuels are assumed to remain stable by volume as of 2020, whereas the remaining liquid hydrocarbon demand, which cannot be substituted by other fuel options, is covered by fossil fuels in the begin of the transition and indirect electrified synthetic fuels by 2050. This requires components such as fossil fuel refineries to represent the cost for converting crude oil into refined fuels, and for synthetic fuels Fischer-Tropsch units, and feeding CO_2_ DAC and water electrolysers, heat recovery and again a supplying electricity system.

##### Biogas and biomethane

The energy system can produce GHG neutral methane for needs of the sectors Power, Heat, Transport and Industry. The first option is upgrading the available biogas to biomethane. The amount of upgraded biogas cannot be more than the urbanization level of the region, but not more than 70% of all biogas. Biomethane can be stored in the gas storage. The second option is power-to-gas. Hydrogen produced with water electrolysis and CO_2_ from DAC units is used as raw material for the methanation units. Produced SNG can be also stored in the gas storage.

##### PV prosumers

The system also includes distributed generation and self-consumption of residential, commercial and industrial electricity consumers (PV prosumers) by installing respective capacities of rooftop PV systems and batteries. For these prosumers the target function is minimal cost of consumed energy calculated as sum of self-generation, annual cost and cost of electricity consumed from the grid, minus benefits from selling of excess energy.

##### Results preparation and cost calculations

All optimisation results are collected and converted from the LP form to the MATLAB structure. This structure contains all information about the system: installed capacities of all system elements, its operation modes, energy, fuel and other products flows.

Data on the structure and operations of the energy system in combination with financial and technical assumptions give the full description of the system. Based on these numbers it is possible to calculate annual costs of each component and the whole system, allocate costs to specific sectors, calculate costs of products (electricity, heat, synthetic fuels, water) and different components of this costs (primary generation, storage, transmission, curtailment components of electricity prices etc.).

The total annualised cost of the system is calculated as sum of all sectors costs (Equation 34), which includes annualized capital cost and operational costs of all system elements (Equation 35):

(Equation 34) $$totalCost_{sys}=elSysCost+elProsCost+heatSysCost+heatIndCost+transpSysCost+industrSysCost$$

(Equation 35) $$totalCost_{sys}=\sum_{t=1}^{tech}\left(CAPEX_{t}\cdot crf_{t}+OPEXfix_{t}\right)\cdot Cap_{t}+OPEXvar_{t}\cdot E_{gen,t}$$

(Equation 36) $$crf_{t}=\;\frac{WACC\cdot {\left(1+WACC\right)}^{N_{t}}}{{\left(1+WACC\right)}^{N_{t}}-1}$$

Abbreviations: total annualised cost of the system (totalCostsys), annualised cost of the centralised power sector (elSysCost), annualised cost of the electricity prosumers sector (elProsCost), annualised cost of the centralised heat sector (heatSysCost), annualised cost of the individual heat sector (heatIndCost), annualised cost of the transport sector (transpSysCost), annualised cost of the industrial sector (industrSysCost), all technologies (tech), technology (t), capital expenditures (CAPEX), capital recovery factor for technology t (crft) Eq. (36), annual fixed operational expenditures (OPEXfix), variable operational expenditures (OPEXvar), installed capacity of the technology t (Capt), annual output for the technology t (Egen,t), weighted average cost of capital (WACC), lifetime for technology t (Nt).

Total levelised cost of electricity in the system (*LCOEtotal*) is calculated as the electricity demand weighted average of the centralised power system LCOE (*LCOEsys*) and prosumers sector LCOE (*LCOEpros*), the formula is presented in (Equation 37). Centralised power system LCOE is comprised of levelised cost of consumed electricity (*LCOEprim*), levelised cost of storage (*LCOS*), levelised cost of curtailed electricity (*LCOC*), levelised cost of electricity transition (*LCOT*) and levelised cost of prosumers feed-in reimbursement (*LCOFS*), (Equation 38). For the prosumers sector total LCOE comprised of the levelized cost of consumed electricity (*LCOEprim*), levelised cost of storage (*LCOS*), and levelised cost of prosumers feed-in reimbursement (*LCOFS*), (Equation 39). Levelised cost of generated electricity is calculated as total annualised cost of the electricity generation system divided by total annual generation (Equation 40), in these calculations operational costs include costs of fuel and GHG emissions cost per unit of the generated electricity, electricity generation systems also include part of fuel production facilities, which are used for fuel production for power system generators. Levelised cost of consumed electricity is calculated based on the cost of the generated electricity (*LCOEgen*), excluding electricity lost due to curtailment, storage and transmission systems losses (Equation 41). Levelised cost of storage is calculated as annualised cost of storage system equipment and annual cost of electricity losses divided by total electricity consumption (Equation 42), storage systems also include part of fuel production facilities, which are used for fuel production for the storage system generators (e.g. for Power-to-Gas – Gas-to-Power). Levelised cost of curtailment is calculated as annual cost of curtailed electricity divided by total electricity consumption (Equation 43). Levelised cost of transmission is calculated area total annualised cost of power grid equipment and annual cost of electricity losses divided by total electricity consumption and multiplied by regional grid utilisation weights (Equation 44), where regional grid utilisation weights are average of region shares in total export and import of energy (Equation 45).

(Equation 37) $$LCOEtotal_{r}=\left(LCOEsys_{r}\cdot E{l_{consSys}}_{r}+LCOEpros_{r}\cdot E{l_{consPros}}_{r}\right)/\left(E{l_{consSys}}_{r}+E{l_{consPros}}_{r}\right)$$

(Equation 38) $$LCOEsys_{r}=LCOEprim_{r}+LCOS_{r}+LCOC_{r}+LCOT_{r}+LCOFS_{r}$$

(Equation 39) $$LCOEpros_{r}=LCOEprim_{r}+LCOS_{r}-LCOFS_{r}$$

(Equation 40) $$LCOEgen_{r}=\;\frac{\sum_{t=1}^{Gen}\left(CAPEX_{t}\cdot crf_{t}+OPEXfix_{t}\right)\cdot Cap_{t,r}+OPEXvar_{t}\cdot El_{gen,t,r}}{El_{gen,r}}$$

(Equation 41) $$LCOEprim_{r}=\;\frac{LCOEgen_{r}\;\cdot \left(El_{gen,r}-\;El_{curt,r}-El_{storLoss,r}-\;El_{transLoss,r}\right)}{El_{cons,r}}$$

(Equation 42) $$LCOS_{r}=\;\frac{\sum_{t=1}^{Stor}\left(CAPEX_{t}\cdot crf_{t}+OPEXfix_{t}\right)\cdot Cap_{t,r}+OPEXvar_{t}\cdot E_{out,t,r}+\;LCOEgen_{r}\cdot El_{storLoss,r}}{El_{cons,r}}$$

(Equation 43) $$LCOC_{r}=\;\frac{LCOEgen_{r}\cdot El_{curt,r}}{El_{cons,r}}$$

(Equation 44) $$LCOT_{r}=\;RegShare\;{\;}_{r}\cdot \frac{\sum_{r}^{Reg}\sum_{t=1}^{trans}\left(CAPEX_{t}\cdot crf_{t}+OPEXfix_{t}\right)\cdot Cap_{t,r}+OPEXvar_{t}\cdot El_{out,t,r}+\;LCOEgen_{r}\cdot El_{transLoss,r}}{El_{cons,r}}$$

(Equation 45) $$RegShare\;{\;}_{r}=0.5\cdot \frac{Import_{r}}{\sum_{r}Import_{r}}+0.5\cdot \frac{Export_{r}}{\sum_{r}Export_{r}}$$

(Equation 46) $$LCOFS_{r}=\;\frac{feedInTarif_{r}\cdot El_{prosTogrid,r}}{El_{cons,r}}$$

Abbreviations: region (r), total levelised cost of electricity in the system (LCOEtotal), centralised system levelised cost of electricity (LCOEsys), prosumers sector levelised cost of electricity (LCOEpros), centralised system electricity consumption (ElconsSys), prosumers sector electricity consumption (ElconsPros), consumed electricity LCOE (LCOEprim), levelised cost of stored electricity (LCOS), levelised cost of curtailed electricity (LCOC), levelised cost of prosumers feed-in reimbursement (LCOFS), generated electricity LCOE (LCOEgen), power generation technologies (Gen), storage technologies (Stor), power transmission technologies (*trans*), technology (t), capital expenditures (CAPEX), capital recovery factor for technology t (crft), annual fixed operational expenditures (OPEXfix), variable operational expenditures (OPEXvar), installed capacity of the technology t (Capt), annual output for the technology t (Elgen,t), annual electricity generation (Elgen), annual electricity curtailment (Elcurt), annual storage loss (ElstorLoss), annual grid loss (EltransLoss), annual electricity consumption (Elcons), annual output of storage t (Eout,t), annual export of grid technology t (Elout,t), electricity exported by region r (Export), electricity imported by region r (Import), feed-in reimbursement (feedInTarif), electricity sold by prosumers to the grid (ELprosTogrid).

The levelised cost of heat (LCOH) is calculated as the weighted average of the centralized and individual systems LCOH of heat (Equation 47). The centralized heat system LCOH (LCOHsys) and individual heat system LCOH (LCOHind) are calculated as annualised cost of heat system equipment and annual cost of electricity consumption by heating equipment divided by total heat consumption Equations 48 and 49. In both formulas, operational expenditures include cost of fuel and GHG emissions per unit of generated heat. The heat systems also include part of fuel production facilities, which are used for fuel production for heat generators. Cogeneration plants costs only included in the power system.

Levelised cost of transportation (LCOM) is calculated as sum of annualised cost of all transport fleet, cost of consumed fuel and electricity, GHG emission cost, divided by transportation demand (Equation 50).

Levelised cost of the industrial sector products (LCOP): levelised cost of Gas (LCOG), liquid fuel (LCOF), water (LCOW), of CO_2_ direct air capture (LCOD) are calculated as sum of annualised cost of the equipment and cost of annually consumed heat, electricity divided by total annual consumption of the product (Equation 51).

(Equation 47) $$LCOHtotal_{r}=\left(LCOHsys_{r}\cdot He_{consSys_{r}}+LCOHind_{r}\cdot He_{consInd_{r}}\right)/\left(He_{consSys_{r}}+He_{consInd_{r}}\;\right)$$

(Equation 48) $$LCOHsys_{r}=\;\frac{\sum_{t=1}^{heat}\left(CAPEX_{t}\cdot crf_{t}+OPEXfix_{t}\right)\cdot Cap_{t,r}+OPEXvar_{t}\cdot He_{out,t,r}+\;LCOEsys_{r}\cdot El_{demSysHeat,r}}{He_{consSys,r}}$$

(Equation 49) $$LCOHind_{r}=\;\frac{\sum_{t=1}^{heat}\left(CAPEX_{t}\cdot crf_{t}+OPEXfix_{t}\right)\cdot Cap_{t,r}+OPEXvar_{t}\cdot He_{out,t,r}+\;ElPrice_{r}\cdot El_{demIndHeat,r}}{He_{consInd,r}}$$

(Equation 50) $$LCOM_{r}=\;\frac{\sum_{t=1}^{Mob}\left(CAPEX_{t}\cdot crf_{t}+OPEXfix_{t}\right)\cdot Cap_{t,r}+\;FuPrice_{t,r}\cdot FuCons_{t,r}}{TR_{dem,r}}$$

(Equation 51) $$LCOP_{r}=\;\frac{\sum_{t=1}^{tech}\left(CAPEX_{t}\cdot crf_{t}+OPEXfix_{t}\right)\cdot Cap_{t,r}+OPEXvar_{t}\cdot Pr_{out,t,r}+\;LCOEsys_{r}\cdot El_{cons,t,r}+\;LCOHsys_{r}\cdot He_{cons,t,r}}{Pr_{cons,r}}$$

Abbreviations: region (r), total levelised cost of heat in the system (LCOHtotal), centralised system levelised cost of heat (LCOEsys), individual heat sector levelised cost of heat (LCOHind), centralised system heat consumption (HeconsSys), individual heat sector heat consumption (HeconsPros), heat generation technologies (heat), transportation technologies (Mob), industrial sector production technologies (tech), technology (t), capital expenditures (CAPEX), capital recovery factor for technology t (crft), annual fixed operational expenditures (OPEXfix), variable operational expenditures (OPEXvar), installed capacity of the technology t (Capt), annual output for the technology t (Heout,t), centralised system levelised cost of electricity (LCOEsys), retail price of electricity (ElPrice), electricity consumed by centralised heat system heaters (EldemSysHeat), electricity consumed by individual heat system heaters (EldemIndHeat), fuel price for transportation technology t (FuPricet), fuel consumption for transportation technology t (FuConst), transportation demand (TRdem), annual product production (Prout), electricity consumption for the production (Elcons), annual heat consumption for the production (Hecons), annual product consumption (Prcons).

#### Other information

##### Financial and technical assumptions

In determining a cost optimal energy system transition pathway, technical and financial assumptions of various technologies are adopted based on market development and insights from scientific literature. In all the scenarios examined, weighted average CoC is set progressively from a maximum of 12% in the initial time step to 11.7%, 11%, 9.8%, 8.5%, 7.5 and 7% in the subsequent time steps. Cost assumptions for all technologies including generation, storage, transmission, and energy conversion are available in Table 2, Table 3, Table 4, Table 5, Table 6, Table 7, Table 8, Table 9.

##### Electricity price and calculation method

The prices of electricity until 2050 for the residential, commercial, and industrial consumers were calculated based on the method described in Gerlach et al. (2014) and. Breyer and Gerlach (2013). Future electricity prices are estimated based on the approach of Gerlach et al. (2014), which assumes that grid electricity prices will increase by 5% per annum for prices less than 0.15 €/kWh, for price in a range of 0.15–0.30 €/kWh by 3% per annum and prices greater than 0.30 €/kWh by 1% per annum. Electricity prices by segments and demand are provided in Tables S2–S5.

##### Capacity limit

Existing capacities until 2020 are set as the lower limit. The lower limit for all technologies is established by data of already installed capacity. However, the upper limit for solar PV, CSP and wind power plants are estimated based on land use limitation and the capacity density. The maximum area covered by solar system (solar PV and CSP) is set to 6% of the total sub-region territory and is set to 4% for the wind power plants. The capacity densities for CSP, solar PV system and wind power plant is 225 MW/km^2^, 75 MW/km^2^ and 8.4 MW/km^2^. Maximum installable capacities are computed by applying the (Equation 52) and (53)

(Equation 52) $$\text{Cap}\;\text{wind}=\text{area}\;\text{total}\cdots \text{limit wind}\cdots \text{P}/\left({\text{d}}_{1}\cdots {\text{d}}_{2}\cdots {\text{d}}_{\text{rot}}^{2}\right)$$

(Equation 53) $$\text{Cap}\;\text{solar}\;=\text{area}\;\text{total}\cdots \text{limit solar}\cdots \left(\eta_{\text{PV}}\cdots \text{GCR}\cdots {\text{I}}_{\text{STC}}\right)\;$$

Where *d*_*rot*_ is the rotor diameter, dimensionless distance constant (*d*_*1*_, *d*_*2*_) set to *d*_*1*_ = 5 and *d*_*2*_ = 7, according to the recommendation based on practical, *η*_*PV*_ is solar PV efficiency, *GCR* is ground cover ratio, and *I*_*STC*_ is insulation at standard test conditions. The upper limits are estimated based on methodology described in Bogdanov and Breyer (2016). The sustainable and economic hydropower potential is obtained from Gernaat et al. (2017). Absolute numbers for lower and upper limits can be found in Tables S6 and S7 and absolute numbers of installed capacities across scenarios are available in Tables S8–S11.

##### Development of energy demand

The development of the energy demand across the sectors of power, heat, transport, and desalination are estimated for Africa. The growth in electricity demand of the power sector through the transition is estimated according to IEA consumption for Africa. Moreover, the hourly electricity load profile is calculated as a fraction of the total demand for each sub-region based on synthetic load data weighted by the sub-region’s population (Toktarova et al., 2019). The power demand is categorised into residential, commercial, and industrial end-users. Several parameters such as existing load data, economic data, annual electricity consumption, annual peak load, temperature, and other country-specific economic data are considered in creating the hourly resolved load for the power sector. Details of the methodology is available in population (Toktarova et al., 2019). Heat demand was divided into domestic hot water heating, biomass for cooking and industrial processes. The heat demand profiles were generated according to Keiner et al. (2021). Further, the heat demand is classified as low, medium, and high temperatures. The transport sector demand is classified according to Khalili et al. (2019) into road, rail, marine and aviation. The desalination demand is projected as a function of water stress index and total water demand is estimated for each year during the transition, according to Caldera and Breyer (2020). All demand data across sectors are provided in Table S12.

##### Renewable energy resource potential analysis

This includes hourly generation profiles of solar PV, wind, and hydropower. Generation profiles for optimally tilted PV, CSP and wind energy are calculated based on methods described in Bogdanov and Breyer (2016) and for single-axis tracking PV according to Afanasyeva et al. (2018), based on resource data of Stackhouse and Whitlock (2008, 2009), reprocessed by the German Aerospace Center (Stetter, 2012). The resource dataset obtained from NASA is in three hourly temporal resolution and spatial resolution of 1° x 1° for the year 2005, which has been reprocessed to a 1 h and 0.45° × 0.45° resolution (Stetter, 2012). The wind feed-in profile was calculated using an Enercon wind turbine (E−101) with 3050 kW rated power and 150 m hub height. The hourly data have been estimated based on the spatial aggregated method is described in Bogdanov and Breyer (2016). It is assumed that 0–10% best areas are weighted by 0.3, 10–20% best areas are weighted by 0.3, 20–30% best areas are weighted by 0.2, 30–40% best areas are weighted by 0.1 and 40–50% best areas are weighted by 0.1%. Figure S5 shows the full load hour (FLH) of solar and wind resources maps for Africa. The generation profiles for hydropower are calculated using the daily resolved water flow data for the year 2005, as a normalized sum of precipitation in the regions. This method leads to a good approximation of the annual generation of hydropower plants (Verzano, 2009).

The potentials for biomass and waste resources were calculated based on the method described in Mensah et al. (2021). Three main biomass sources are considered, namely, forestry (forest residues), agriculture (crop residue, and livestock manure), fecal sewage sludge (FSS), and municipal waste (MSW) to avoid violation of biomass sustainability criteria. Bioenergy from crop and forest residues is estimated using the residue to product ratio (RPR) parameter. The use factor applied for crop and wood residues is 35 and 80%, respectively. The lower heating values are considered for the energy potential estimation. Energy from MSW and livestock manure was estimated in an anaerobic digestion process for the biodegradable portion of the waste. While the non-biogenic fraction of MSW is assumed to be treated with an incineration process for bioenergy use.

The costs for biomass are calculated using data from the International Energy Agency (IEA, 2012) and the Intergovernmental Panel on Climate Change (IPCC, 2011). A gate fee of 50 €/ton gate fee was assumed for solid waste for the year 2020, which increased up to 100 €/ton in 2050. Geothermal energy potential is estimated according to the method described by Aghahosseini and Breyer (2018).

## Data Availability

• The model setup, input data and output data are available in the supplementary information. Any additional information will be provided by the lead contact upon request. • This paper does not report original code.

## Supplemental information

Docuement S1. Figures S1–S25 and Tables S1–S12
