# Supplementary material for: Contextualizing the scope, scale, and speed of energy pathways toward sustainable development in Africa
Source: iScience. 2022 Sep 6;25(9):104965. doi: 10.1016/j.isci.2022.104965 (PMC9486755; doi:10.1016/j.isci.2022.104965)
Supplement: Docuement S1. Figures S1–S25 and Tables S1–S12 [file mmc1.pdf]

iScience, Volume *25*

Supplemental information

Contextualizing the scope, scale, and speed of energy pathways toward sustainable development in Africa

Ayobami S. Oyewo, Dmitrii Bogdanov, Arman Aghahosseini, Theophilus N.O. Mensah, and Christian Breyer

Table S1: All regions and macro region structure for Africa, related to Figure 1.

Macro region	Region's code	Countries
North Africa (AFN)	DZ	Algeria
	EG	Egypt
	LY	Libya
	MA	Morocco
	TN	Tunisia
	MW	Mauritania, Western Sahara
West Africa (AFW)	WW	Senegal, Gambia, Cape Verde Islands, Guinea Bissau, Guinea, Sierra Leone, Liberia
	ML	Mali
	WS	Ghana, Cote D'Ivoire, Benin, Burkina Faso, Togo
	NE	Niger
	NIG-S	Nigeria South
	NIG-N	Nigeria North
East Africa (AFE)	SER	Sudan, South Sudan, Eritrea
	ETH	Ethiopia
	SOMDJ	Djibouti, Somalia
	KENUG	Kenya, Uganda
	TZRB	Rwanda, Burundi, Tanzania
Central Africa (AFC)	TD	Chad
	CAR	Central African Republic, Cameroon, Equatorial Guinea, Sao Tome and Principe, Congo, Republic of, Gabon
	COG	Congo, Democratic Republic
South Africa (AFS)	SW	Angola, Namibia, Botswana
	ZAFLS	Republic of South Africa, Lesotho
	SE	Malawi, Mozambique, Zambia, Zimbabwe, Swaziland
Indian Ocean	IOCE	Comoros Islands, Mauritius, Mayotte, Madagascar, Seychelles

Table S2: Electricity prices for residential sector, related to Star Methods.

Electricity price - Residential sector									
	**All regions**	**Unit**	**2020**	**2025**	**2030**	**2035**	**2040**	**2045**	**2050**
**Macro region**	AFN	€/MWh	80	97	114	135	156	176	196
	AFW	€/MWh	98	117	137	160	179	200	222
	AFE	€/MWh	132	156	180	207	237	262	278
	AFC	€/MWh	121	145	165	185	208	233	252
	AFS	€/MWh	66	83	101	124	149	174	198

	IOCE	€/MWh	86	110	135	153	177	205	236
All regions	DZ	€/MWh	63	80	102	130	151	175	203
	EG	€/MWh	26	33	42	53	68	86	110
	LY	€/MWh	15	20	25	32	41	52	66
	MA	€/MWh	136	158	183	213	246	269	283
	TN	€/MWh	103	132	153	177	206	238	266
	MW	€/MWh	136	156	180	203	221	235	249
	WW	€/MWh	136	156	180	203	221	235	249
	ML	€/MWh	168	194	225	261	275	289	303
	WS	€/MWh	119	143	170	194	217	241	268
	NE	€/MWh	111	136	157	183	212	245	268
	NIG-S	€/MWh	28	36	46	58	74	95	121
	NIG-N	€/MWh	28	36	46	58	74	95	121
	SER	€/MWh	120	144	167	194	225	260	273
	ETH	€/MWh	134	156	180	209	242	265	278
	SOMDJ	€/MWh	138	160	185	215	249	270	283
	KENUG	€/MWh	168	195	218	244	270	284	298
	TZRB	€/MWh	101	127	148	172	200	232	257
	TD	€/MWh	209	243	265	279	293	308	324
	CAR	€/MWh	110	135	157	183	212	246	265
	COG	€/MWh	45	57	73	94	119	144	167
	SW	€/MWh	75	93	113	137	163	191	213
	ZAFLS	€/MWh	44	56	71	90	115	137	159
	SE	€/MWh	78	99	120	144	168	193	221
	IOCE	€/MWh	86	110	135	153	177	205	236

Table S3: Electricity prices for commercial sector, related to Star Methods.

Electricity price - Commercial sector									
	**All regions**	**Unit**	**2020**	**2025**	**2030**	**2035**	**2040**	**2045**	**2050**
**Macro region**	AFN	€/MWh	82	99	118	139	161	183	204
	AFW	€/MWh	106	126	150	174	195	215	237
	AFE	€/MWh	113	138	163	191	221	250	274
	AFC	€/MWh	127	151	174	199	222	246	265
	AFS	€/MWh	58	74	92	111	134	158	184
	IOCE	€/MWh	84	107	133	150	173	201	232
**Allregions**	DZ	€/MWh	64	82	105	133	154	178	207
	EG	€/MWh	38	49	62	80	100	120	144
	LY	€/MWh	20	25	32	41	52	67	85
	MA	€/MWh	132	153	177	206	238	266	279
	TN	€/MWh	97	124	146	169	196	228	259
	MW	€/MWh	139	159	184	207	224	238	251
	WW	€/MWh	139	159	184	207	224	238	251
	ML	€/MWh	166	192	223	259	272	286	300
	WS	€/MWh	135	159	186	214	241	260	280

	NE	€/MWh	96	120	145	168	195	226	254
	NIG-S	€/MWh	50	64	81	99	119	142	170
	NIG-N	€/MWh	50	64	81	99	119	142	170
	SER	€/MWh	85	104	124	148	178	206	224
	ETH	€/MWh	100	120	144	171	199	222	243
	SOMDJ	€/MWh	105	126	151	179	207	231	253
	KENUG	€/MWh	168	204	241	275	312	347	387
	TZRB	€/MWh	109	135	156	181	210	242	264
	TD	€/MWh	187	217	243	267	281	295	310
	CAR	€/MWh	119	142	165	192	222	251	268
	COG	€/MWh	76	96	114	137	164	193	216
	SW	€/MWh	62	78	96	115	139	166	191
	ZAFLS	€/MWh	35	45	57	73	92	113	136
	SE	€/MWh	78	99	122	146	170	196	226
	IOCE	€/MWh	84	107	133	150	173	201	232

Table S4: Electricity prices for industrial sector, related to Star Methods.

**Electricity price - Industrial sector**									
	**All regions**	**Unit**	**2020**	**2025**	**2030**	**2035**	**2040**	**2045**	**2050**
**Macro region**	AFN	€/MWh	84	101	122	144	166	189	212
	AFW	€/MWh	114	136	163	189	211	231	253
	AFE	€/MWh	85	107	133	159	187	216	246
	AFC	€/MWh	133	158	183	212	237	260	278
	AFS	€/MWh	51	65	82	99	119	143	171
	IOCE	€/MWh	81	104	130	146	170	197	228
**All regions**	DZ	€/MWh	66	85	108	135	157	182	211
	EG	€/MWh	51	65	83	106	133	154	179
	LY	€/MWh	24	31	40	50	64	82	105
	MA	€/MWh	128	148	172	199	231	262	275
	TN	€/MWh	91	116	139	162	187	217	252
	MW	€/MWh	142	162	187	212	226	240	252
	WW	€/MWh	142	162	187	212	226	240	252
	ML	€/MWh	164	190	221	256	269	283	297
	WS	€/MWh	152	176	203	234	265	279	293
	NE	€/MWh	81	104	133	154	178	207	240
	NIG-S	€/MWh	72	92	117	141	163	189	219
	NIG-N	€/MWh	72	92	117	141	163	189	219
	SER	€/MWh	50	63	81	103	130	151	175
	ETH	€/MWh	66	84	107	134	155	180	209
	SOMDJ	€/MWh	72	92	117	143	165	192	222
	KENUG	€/MWh	123	157	195	227	263	305	354
	TZRB	€/MWh	117	142	165	191	220	253	271
	TD	€/MWh	164	190	221	256	269	283	297
	CAR	€/MWh	128	149	174	201	233	257	272

	COG	€/MWh	107	134	155	180	209	242	264
	SW	€/MWh	49	63	78	94	115	140	169
	ZAFLS	€/MWh	27	34	43	55	70	89	113
	SE	€/MWh	78	100	125	147	172	199	231
	IOCE	€/MWh	81	104	130	146	170	197	228

Table S5: Electricity demand in regional and macro region resolution,

**Electricity demand**									
	**All regions**	**Unit**	**2020**	**2025**	**2030**	**2035**	**2040**	**2045**	**2050**
**Macro region**	AFN	TWh	267	310	360	418	486	564	656
	AFW	TWh	73	104	148	212	303	433	619
	AFE	TWh	51	73	106	152	220	317	457
	AFC	TWh	22	29	39	51	68	89	118
	AFS	TWh	276	312	353	400	452	512	580
	IOCE	TWh	6	9	13	19	28	40	58
**All regions**	DZ	TWh	42	49	57	66	77	89	103
	EG	TWh	151	175	203	235	273	316	367
	LY	TWh	34	40	46	54	62	72	83
	MA	TWh	26	30	35	41	47	55	64
	TN	TWh	12	14	16	19	22	25	29
	MW	TWh	1	2	3	4	5	7	10
	WW	TWh	7	10	14	20	28	40	56
	ML	TWh	2	3	4	5	7	10	14
	WS	TWh	27	37	53	74	105	148	208
	NE	TWh	1	2	2	3	5	7	9
	NIG-S	TWh	23	34	49	70	102	148	214
	NIG-N	TWh	13	18	27	39	56	81	117
	SER	TWh	17	24	35	50	73	105	151
	ETH	TWh	10	15	22	31	45	65	93
	SOMDJ	TWh	1	1	2	3	4	6	8
	KENUG	TWh	14	21	30	43	62	90	129
	TZRB	TWh	8	12	18	25	36	53	76
	TD	TWh	0	0	0	1	1	1	1
	CAR	TWh	13	17	22	29	38	50	66
	COG	TWh	9	12	16	22	29	38	50
	SW	TWh	18	21	24	29	33	39	45
	ZAFLS	TWh	212	238	267	299	335	375	420
	SE	TWh	46	53	62	72	84	98	114
	IOCE	TWh	6	9	13	19	28	40	58

Table S6: Renewable energy lower and upper capacity limits for Africa, related to Star Methods.

		PV optimally tilted		PV single-axis tracking		Concentrated solar power	
**All regions**	**Unit**	**Lower limit**	**Upper limit**	**Lower limit**	**Upper limit**	**Lower limit**	**Upper limit**
DZ	GW	0.41	10719.00	0.00	10719.00	0.03	21438.00
EG	GW	0.75	4545.00	0.00	4545.00	0.02	9090.00
LY	GW	0.02	7920.00	0.00	7920.00	0.00	15840.00
MA	GW	0.21	2009.48	0.00	2009.48	0.53	4018.95
TN	GW	0.05	736.25	0.00	736.25	0.00	1472.49
MW	GW	0.09	5832.00	0.00	5832.00	0.00	11664.00
WW	GW	0.17	3045.22	0.00	3045.22	0.00	6090.44
ML	GW	0.02	5580.00	0.00	5580.00	0.00	11160.00
WS	GW	0.14	4530.33	0.00	4530.33	0.00	9060.65
NE	GW	0.02	5700.15	0.00	5700.15	0.00	11400.30
NIG-S	GW	0.00	857.05	0.00	857.05	0.00	1714.10
NIG-N	GW	0.02	3271.41	0.00	3271.41	0.00	6542.81
SER	GW	0.02	11805.04	0.00	11805.04	0.00	23610.09
ETH	GW	0.01	4563.00	0.00	4563.00	0.00	9126.00
SOMDJ	GW	0.01	2973.85	0.00	2973.85	0.00	5947.70
KENUG	GW	0.14	3696.32	0.00	3696.32	0.00	7392.64
TZRB	GW	0.06	4496.67	0.00	4496.67	0.00	8993.33
TD	GW	0.00	5778.00	0.00	5778.00	0.00	11556.00
CAR	GW	0.02	7813.56	0.00	7813.56	0.00	15627.12
COG	GW	0.01	10552.50	0.00	10552.50	0.00	21105.00
SW	GW	0.10	12027.55	0.00	12027.55	0.00	24055.10
ZAFLS	GW	2.56	9363.66	0.00	9363.66	0.40	18727.32
SE	GW	0.06	5626.60	0.00	5626.60	0.00	11253.20
IOCE	GW	0.26	2677.67	0.00	2677.67	0.00	5355.34
**All regions**	**Unit**	**Wind onshore**		**Hydropower reservoir**		**Hydropower RoR**	
DZ	GW	0.01	800.35	0.18	0.18	0.09	0.18
EG	GW	1.13	339.36	1.96	1.96	0.89	1.33
LY	GW	0.00	591.36	0.00	0.00	0.00	0.00
MA	GW	1.22	150.04	0.93	0.93	0.45	1.34
TN	GW	0.25	54.97	0.06	0.06	0.00	0.01
MW	GW	0.03	435.46	0.03	0.03	0.02	0.03
WW	GW	0.03	227.38	0.53	0.53	0.04	3.66
ML	GW	0.00	416.64	0.15	0.15	0.03	0.46
WS	GW	0.00	338.26	2.10	2.10	0.45	1.87
NE	GW	0.00	425.61	0.00	0.00	0.00	0.70
NIG-S	GW	0.00	63.99	0.00	0.00	0.00	3.07
NIG-N	GW	0.00	244.26	1.36	1.36	0.61	0.78
SER	GW	0.00	881.44	1.93	1.93	0.01	1.64
ETH	GW	0.32	340.70	3.67	3.67	0.15	7.85
SOMDJ	GW	0.00	222.05	0.00	0.00	0.00	0.00
KENUG	GW	0.34	275.99	1.02	1.02	0.29	0.66
TZRB	GW	0.00	335.75	0.34	0.34	0.37	2.05
TD	GW	0.00	431.42	0.00	0.00	0.00	0.00

CAR	GW	0.00	583.41	0.65	0.65	0.77	44.12
COG	GW	0.00	787.92	0.96	0.96	1.78	25.16
SW	GW	0.01	898.06	3.20	3.20	0.06	6.71
ZAFLS	GW	2.09	699.15	0.61	0.61	0.07	19.63
SE	GW	0.00	420.12	5.68	5.68	0.54	2.63
IOCE	GW	0.03	199.93	0.07	0.07	0.04	6.77

Table S7: Fossil fuels and nuclear power lower and upper capacity limits for Africa, related to Star Methods.

		**Coal**	**Oil**	**Natural gas**	**Nuclear**
**All regions**	**Unit**	**Lower limit**	**Lower limit**	**Lower limit**	**Lower limit**
DZ	GW	0.00	0.30	15.89	0
EG	GW	0.00	1.06	38.33	0
LY	GW	0.00	4.03	6.96	0
MA	GW	2.87	2.03	0.92	0
TN	GW	0.00	1.57	3.06	0
MW	GW	0.00	0.33	0.12	0
WW	GW	0.13	1.39	0.32	0
ML	GW	0.00	0.33	0.04	0
WS	GW	0.00	0.86	3.37	0
NE	GW	0.04	0.25	0.02	0
NIG-S	GW	0.00	0.04	4.44	0
NIG-N	GW	0.00	0.00	1.15	0
SER	GW	0.11	2.42	1.14	0
ETH	GW	0.00	0.15	0.14	0
SOMDJ	GW	0.00	0.20	0.00	0
KENUG	GW	0.00	1.17	0.19	0
TZRB	GW	0.00	0.62	0.85	0
TD	GW	0.00	0.63	0.00	0
CAR	GW	0.00	0.55	0.68	0
COG	GW	0.00	0.41	0.00	0
SW	GW	0.72	0.91	0.42	0
ZAFLS	GW	45.01	2.14	3.68	1.86
SE	GW	1.18	0.71	1.29	0
IOCE	GW	0.40	1.00	0.13	0
NL- No upper limit specified					

Table S8: Key power plant capacities required for the BPS, related to Star Methods.

Technology	unit	2020	2025	2030	2035	2040	2045	2050
PV prosumers	[GW_{el}]	0	6	27	80	203	344	536
PV utility–scale	[GW_{el}]	5	67	190	624	1697	3137	3590
Wind onshore	[GW_{el}]	6	17	40	116	166	170	158
Hydro run–of–river	[GW_{el}]	7	7	7	7	7	7	7
Hydro reservoir (dam)	[GW_{el}]	25	33	37	37	37	37	37
Biomass solid	[GW_{el}]	2	1	1	1	0	0	0
Geothermal electricity	[GW_{el}]	0	0	2	2	2	2	2
Biomass CHP	[GW_{el}]	0	0	0	0	0	0	0
Waste–to–energy CHP	[GW_{el}]	0	1	2	3	3	3	3
Biogas CHP	[GW_{el}]	0	0	1	3	8	12	15
CSP ST	[GW_{el}]	0	0	0	0	0	0	0
CCGT	[GW_{el}]	37	41	45	44	41	32	28
OCGT	[GW_{el}]	45	55	110	289	654	646	638
ICE	[GW_{el}]	22	17	12	11	5	3	0
Coal PP hard coal	[GW_{el}]	47	38	30	19	15	10	9
Nuclear PP	[GW_{el}]	2	2	2	2	2	2	0
Methane CHP	[GW_{el}]	1	23	32	32	31	31	31
Oil CHP	[GW_{el}]	0	0	0	0	0	0	0
Coal CHP	[GW_{el}]	3	3	2	1	1	0	0
Methane CHP	[GW_{th}]	1	33	45	45	44	44	44
Methane DH	[GW_{th}]	44	44	44	36	28	20	12
Methane IH	[GW_{th}]	21	13	9	6	0	0	0
Oil CHP	[GW_{th}]	0	0	0	0	0	0	0
Oil DH	[GW_{th}]	56	56	56	47	39	30	21
Oil IH	[GW_{th}]	38	29	25	15	1	0	1
Coal CHP	[GW_{th}]	3	2	2	1	1	0	0
Coal DH	[GW_{th}]	15	15	15	12	10	7	5
CSP SF	[GW_{th}]	1	1	1	1	1	0	0
Solar thermal heat	[GW_{th}]	0	0	0	0	0	0	0
Geothermal heat DH	[GW_{th}]	0	0	0	0	0	0	0
Biomass CHP	[GW_{th}]	0	0	0	0	0	0	0
Biomass DH	[GW_{th}]	42	47	47	42	35	28	20
Biomass IH	[GW_{th}]	53	53	54	61	70	83	95
Waste–to–energy CHP	[GW_{th}]	0	0	1	1	1	1	1
Biogas CHP	[GW_{th}]	0	0	1	3	7	10	12
Biogas IH	[GW_{th}]	0	3	3	3	3	3	3
Electric heating DH	[GW_{th}]	0	32	61	95	112	127	150
Electric heating IH	[GW_{th}]	27	29	24	23	27	24	23
Heat pump DH	[GW_{th}]	0	18	29	39	48	48	36
Heat pump IH	[GW_{th}]	0	7	9	12	18	19	20
Water electrolysis	[GW_{el}]	0	0	11	120	683	1331	1501
Water electrolysis	[GW_{H2}]	0	0	9	99	561	1094	1234
CO_2 DAC	[$MtCO_{2/a}$]	0	0	16	38	55	55	55
Methanation	[GW_{CH4}]	0	0	31	99	339	481	492
Fischer–Tropsch	[GW_{liq}]	0	0	0	0	0	12	34
Water electrolysis	[GW_{el}]	0	0	12	40	136	189	191
Battery prosumer	[GWh]	0	3	32	100	307	574	876
Battery	[GWh]	0	1	23	529	1368	2233	2856
PHES	[GWh]	3	3	3	5	5	5	5
A-CAES	[GWh]	0	0	0	0	0	0	1
TES HT	[GWh]	0	70	182	360	457	536	645
TES DH	[GWh]	0	16	35	83	282	391	396
Gas (H_2) storage	[GWh]	0	0	500	3300	16600	48600	49200
Gas (CH_4) storage	[GWh]	0	2	748	3341	10263	38950	40840
Biogas storage	[GWh]	0	0	0	100	200	400	500

Table S9: Key power plant capacities required for the BPSnoCC, related to Star Methods.

Technology	unit	2020	2025	2030	2035	2040	2045	2050
PV prosumers	[GW_{el}]	0	6	27	80	203	344	536
PV utility–scale	[GW_{el}]	5	67	132	314	698	1059	1370
Wind onshore	[GW_{el}]	6	7	27	61	99	98	97
Hydro run–of–river	[GW_{el}]	7	7	7	7	7	7	7
Hydro reservoir (dam)	[GW_{el}]	25	28	34	37	37	37	37
Biomass solid	[GW_{el}]	2	1	1	1	0	0	0
Geothermal electricity	[GW_{el}]	0	0	0	1	1	1	1
Biomass CHP	[GW_{el}]	0	0	0	0	0	0	0
Waste–to–energy CHP	[GW_{el}]	0	1	2	3	3	3	3
Biogas CHP	[GW_{el}]	0	0	1	2	6	11	15
CSP ST	[GW_{el}]	0	0	0	0	0	0	0
CCGT	[GW_{el}]	37	44	50	69	73	70	66
OCGT	[GW_{el}]	45	43	43	88	158	151	168
ICE	[GW_{el}]	22	17	12	11	5	3	0
Coal PP hard coal	[GW_{el}]	47	40	33	23	18	14	13
Nuclear PP	[GW_{el}]	2	2	2	2	2	2	0
Methane CHP	[GW_{el}]	1	23	40	42	41	41	41
Oil CHP	[GW_{el}]	0	0	0	0	0	0	0
Coal CHP	[GW_{el}]	3	3	2	1	1	0	0
Methane CHP	[GW_{th}]	1	33	45	45	44	44	44
Methane DH	[GW_{th}]	44	44	44	36	28	20	12
Methane IH	[GW_{th}]	21	13	9	6	0	0	0
Oil CHP	[GW_{th}]	0	0	0	0	0	0	0
Oil DH	[GW_{th}]	56	56	56	47	39	30	21
Oil IH	[GW_{th}]	38	29	25	15	1	0	1
Coal CHP	[GW_{th}]	3	2	2	1	1	0	0
Coal DH	[GW_{th}]	15	15	15	12	10	7	5
CSP SF	[GW_{th}]	1	1	1	1	1	0	0
Solar thermal heat	[GW_{th}]	0	0	0	0	0	0	0
Geothermal heat DH	[GW_{th}]	0	0	0	0	0	0	0
Biomass CHP	[GW_{th}]	0	0	0	0	0	0	0
Biomass DH	[GW_{th}]	42	47	47	42	35	28	20
Biomass IH	[GW_{th}]	53	53	54	61	70	83	95
Waste–to–energy CHP	[GW_{th}]	0	0	1	1	1	1	1
Biogas CHP	[GW_{th}]	0	0	1	3	7	10	12
Biogas IH	[GW_{th}]	0	3	3	3	3	3	3
Electric heating DH	[GW_{th}]	0	32	61	95	112	127	150
Electric heating IH	[GW_{th}]	27	29	24	23	27	24	23
Heat pump DH	[GW_{th}]	0	18	29	39	48	48	36
Heat pump IH	[GW_{th}]	0	7	9	12	18	19	20
Water electrolysis	[GW_{el}]	0	0	0	0	0	0	0
Water electrolysis	[GW_{H2}]	0	0	0	0	0	0	0
Steam methane reforming	[GW_{H2}]	0	0	1	4	16	48	108
CO_2 DAC	[$MtCO_{2/a}$]	0	0	0	0	0	0	0
Methanation	[GW_{CH4}]	0	0	0	0	0	0	0
Fischer–Tropsch	[GW_{liq}]	0	0	0	0	0	0	0
Water electrolysis	[GW_{el}]	0	0	0	0	0	0	0
Battery prosumer	[GWh]	0	3	32	100	307	574	876
Battery	[GWh]	0	6	15	241	1545	2868	3915
PHES	[GWh]	3	3	3	3	5	5	5
A-CAES	[GWh]	0	0	0	0	0	0	0
TES HT	[GWh]	0	69	144	294	425	565	779

TES DH	[GWh]	0	29	69	82	157	131	94
Gas (H_2) storage	[GWh]	0	0	0	0	0	0	0
Gas (CH_4) storage	[GWh]	0	1	1	903	2527	4026	4118
Biogas storage	[GWh]	0	0	0	100	300	600	800

Table S10: Key power plant capacities required for the CPS, related to Star Methods.

Technology	unit	2020	2025	2030	2035	2040	2045	2050
PV prosumers	[GW_{el}]	0	6	27	80	203	344	536
PV utility–scale	[GW_{el}]	5	27	63	106	152	187	242
Wind onshore	[GW_{el}]	6	10	23	40	67	92	128
Hydro run–of–river	[GW_{el}]	7	7	7	10	18	18	18
Hydro reservoir (dam)	[GW_{el}]	25	41	52	60	65	74	83
Biomass solid	[GW_{el}]	2	1	1	1	0	0	0
Geothermal electricity	[GW_{el}]	0	0	1	1	1	0	0
Biomass CHP	[GW_{el}]	0	0	0	0	0	0	0
Waste–to–energy CHP	[GW_{el}]	0	1	2	3	3	3	3
Biogas CHP	[GW_{el}]	0	0	0	1	2	4	10
CSP ST	[GW_{el}]	0	0	0	0	0	0	0
CCGT	[GW_{el}]	37	35	36	37	37	35	51
OCGT	[GW_{el}]	45	38	34	29	25	14	6
ICE	[GW_{el}]	22	19	22	24	27	31	34
Coal PP hard coal	[GW_{el}]	47	42	35	35	36	36	39
Nuclear PP	[GW_{el}]	2	2	4	4	6	6	8
Methane CHP	[GW_{el}]	1	19	40	43	46	50	50
Oil CHP	[GW_{el}]	0	0	0	0	0	0	0
Coal CHP	[GW_{el}]	3	3	2	1	1	0	0
Methane CHP	[GW_{th}]	1	26	56	60	64	70	70
Methane DH	[GW_{th}]	44	44	44	36	28	20	12
Methane IH	[GW_{th}]	21	13	9	6	0	0	0
Oil CHP	[GW_{th}]	0	0	0	0	0	0	0
Oil DH	[GW_{th}]	56	56	56	47	39	30	21
Oil IH	[GW_{th}]	38	29	25	15	1	0	1
Coal CHP	[GW_{th}]	3	2	2	1	1	0	0
Coal DH	[GW_{th}]	15	15	15	12	10	7	5
CSP SF	[GW_{th}]	1	1	1	1	1	0	0
Solar thermal heat	[GW_{th}]	0	0	0	0	0	0	0
Geothermal heat DH	[GW_{th}]	0	0	0	0	0	0	0
Biomass CHP	[GW_{th}]	0	0	0	0	0	0	0
Biomass DH	[GW_{th}]	42	58	58	50	49	42	37
Biomass IH	[GW_{th}]	53	53	54	61	70	83	95
Waste–to–energy CHP	[GW_{th}]	0	0	1	1	1	1	1
Biogas CHP	[GW_{th}]	0	0	0	0	2	3	8
Biogas IH	[GW_{th}]	0	3	3	3	3	3	3
Electric heating DH	[GW_{th}]	0	22	33	47	79	104	129
Electric heating IH	[GW_{th}]	27	29	24	23	27	24	23
Heat pump DH	[GW_{th}]	0	10	18	25	28	37	46
Heat pump IH	[GW_{th}]	0	7	9	12	18	19	20
Water electrolysis	[GW_{el}]	0	0	0	0	0	0	0
Water electrolysis	[GW_{H2}]	0	0	0	0	0	0	0
Steam reforming	[GW_{H2}]	0	1	1	1	1	1	1
CO_2 DAC	[$MtCO_{2/a}$]	0	0	0	0	0	0	0
Methanation	[GW_{CH4}]	0	0	0	0	0	0	0
Fischer–Tropsch	[GW_{liq}]	0	0	0	0	0	0	0
Water electrolysis	[GW_{el}]	0	0	0	0	0	0	0
Battery prosumer	[GWh]	0	3	32	100	307	574	876
Battery	[GWh]	0	5	5	17	85	243	461

PHES	[GWh]	3	3	3	3	5	5	5
A-CAES	[GWh]	0	0	0	0	0	0	0
TES HT	[GWh]	0	18	36	101	238	376	485
TES DH	[GWh]	0	14	25	59	101	125	132
Gas (H_2) storage	[GWh]	0	0	0	0	0	0	0
Gas (CH_4) storage	[GWh]	0	3	10	11	57	128	222
Biogas storage	[GWh]	0	0	0	0	100	200	300

Table S11: Key power plant capacities required for the CPSnoCC, related to Star Methods.

Technology	unit	2020	2025	2030	2035	2040	2045	2050
PV prosumers	[GW_{el}]	0	6	27	80	203	344	536
PV utility–scale	[GW_{el}]	5	26	56	92	116	167	244
Wind onshore	[GW_{el}]	6	10	22	38	58	88	126
Hydro run–of–river	[GW_{el}]	7	7	7	7	7	7	7
Hydro reservoir (dam)	[GW_{el}]	25	37	46	53	61	74	85
Biomass solid	[GW_{el}]	2	1	1	1	0	0	0
Geothermal electricity	[GW_{el}]	0	0	1	1	1	0	0
Biomass CHP	[GW_{el}]	0	0	0	0	0	0	0
Waste–to–energy CHP	[GW_{el}]	0	1	2	3	3	3	3
Biogas CHP	[GW_{el}]	0	0	0	0	3	5	5
CSP ST	[GW_{el}]	0	0	0	0	0	0	0
CCGT	[GW_{el}]	37	36	35	34	35	39	51
OCGT	[GW_{el}]	45	38	34	29	25	17	9
ICE	[GW_{el}]	22	18	21	22	20	23	26
Coal PP hard coal	[GW_{el}]	47	43	36	36	34	34	37
Nuclear PP	[GW_{el}]	2	2	4	4	6	6	8
Methane CHP	[GW_{el}]	1	19	34	37	39	40	40
Oil CHP	[GW_{el}]	0	0	0	0	0	0	0
Coal CHP	[GW_{el}]	3	3	2	1	1	0	0
Methane CHP	[GW_{th}]	1	26	56	60	64	70	70
Methane DH	[GW_{th}]	44	44	44	36	28	20	12
Methane IH	[GW_{th}]	21	13	9	6	0	0	0
Oil CHP	[GW_{th}]	0	0	0	0	0	0	0
Oil DH	[GW_{th}]	56	56	56	47	39	30	21
Oil IH	[GW_{th}]	38	29	25	15	1	0	1
Coal CHP	[GW_{th}]	3	2	2	1	1	0	0
Coal DH	[GW_{th}]	15	15	15	12	10	7	5
CSP SF	[GW_{th}]	1	1	1	1	1	0	0
Solar thermal heat	[GW_{th}]	0	0	0	0	0	0	0
Geothermal heat DH	[GW_{th}]	0	0	0	0	0	0	0
Biomass CHP	[GW_{th}]	0	0	0	0	0	0	0
Biomass DH	[GW_{th}]	42	58	58	50	49	42	37
Biomass IH	[GW_{th}]	53	53	54	61	70	83	95
Waste–to–energy CHP	[GW_{th}]	0	0	1	1	1	1	1
Biogas CHP	[GW_{th}]	0	0	0	0	2	3	8
Biogas IH	[GW_{th}]	0	3	3	3	3	3	3
Electric heating DH	[GW_{th}]	0	22	33	47	79	104	129
Electric heating IH	[GW_{th}]	27	29	24	23	27	24	23
Heat pump DH	[GW_{th}]	0	10	18	25	28	37	46
Heat pump IH	[GW_{th}]	0	7	9	12	18	19	20
Water electrolysis	[GW_{el}]	0	0	0	0	0	0	0
Water electrolysis	[GW_{H2}]	0	0	0	0	0	0	0
Steam reforming	[GW_{H2}]	0	1	1	1	1	1	1
CO_2 DAC	[$MtCO_{2/a}$]	0	0	0	0	0	0	0
Methanation	[GW_{CH4}]	0	0	0	0	0	0	0
Fischer–Tropsch	[GW_{liq}]	0	0	0	0	0	0	0

Water electrolysis	[GW_{el}]	0	0	0	0	0	0	0
Battery prosumer	[GWh]	0	3	32	100	307	574	876
Battery	[GWh]	0	0	2	15	41	182	432
PHES	[GWh]	3	3	3	3	5	5	5
A-CAES	[GWh]	0	0	0	0	0	0	0
TES HT	[GWh]	0	19	36	76	164	363	488
TES DH	[GWh]	0	23	66	81	103	133	128
Gas (H_2) storage	[GWh]	0	0	0	0	0	0	0
Gas (CH_4) storage	[GWh]	0	2	5	7	168	284	292
Biogas storage	[GWh]	0	0	0	0	100	200	200

Table S12: Power, heat, desalination, and transport demands for all regions from 2020 to 2050 for the BPS, related to Star Methods.

**Electricity demand**	**Unit**	**2020**	**2025**	**2030**	**2035**	**2040**	**2045**	**2050**
Power Sector	[TWh_{el}]	797	948	1,132	1,364	1,661	2,066	2,621
Other sectors	[TWh_{el}]	39	286	504	992	3,098	4,935	5,409
**Heat demand**								
Industrial process heat demand	[TWh_{th}]	723	816	1,068	1,131	1,216	1,369	1,529
Space heating heat demand	[TWh_{th}]	67	73	68	74	77	74	70
Domestic water heating heat demand	[TWh_{th}]	204	249	302	362	430	504	587
Biomass cooking heat demand	[TWh_{th}]	3,039	2,209	1,339	661	271	71	0
**Desalination demand**	mil m^3/day	14	30	48	60	72	97	115
**Transportation demand**								
Road passenger	[mil p-km]	490,425	575,454	697,506	861,019	1,066,605	1,321,310	1,617,220
Road freight	[mil t-km]	346,537	398,294	467,226	555,142	660,679	781,889	919,100
Rail passenger	[mil p-km]	30,451	34,082	38,871	44,833	51,731	59,261	67,241
Rail freight	[mil t-km]	173,962	190,840	210,994	234,826	262,345	293,614	329,377
Marine passenger	[mil p-km]	9,532	11,424	14,059	17,652	22,385	28,343	35,549
Marine freight	[mil t-km]	5,433,718	7,558,846	10,868,579	15,855,919	23,000,875	32,759,985	45,689,633
Aviation passenger	[mil p-km]	215,468	284,780	397,239	531,893	739,539	977,487	1,226,628
Aviation freight	[mil t-km]	16,647	26,427	43,431	72,410	121,761	196,620	301,078

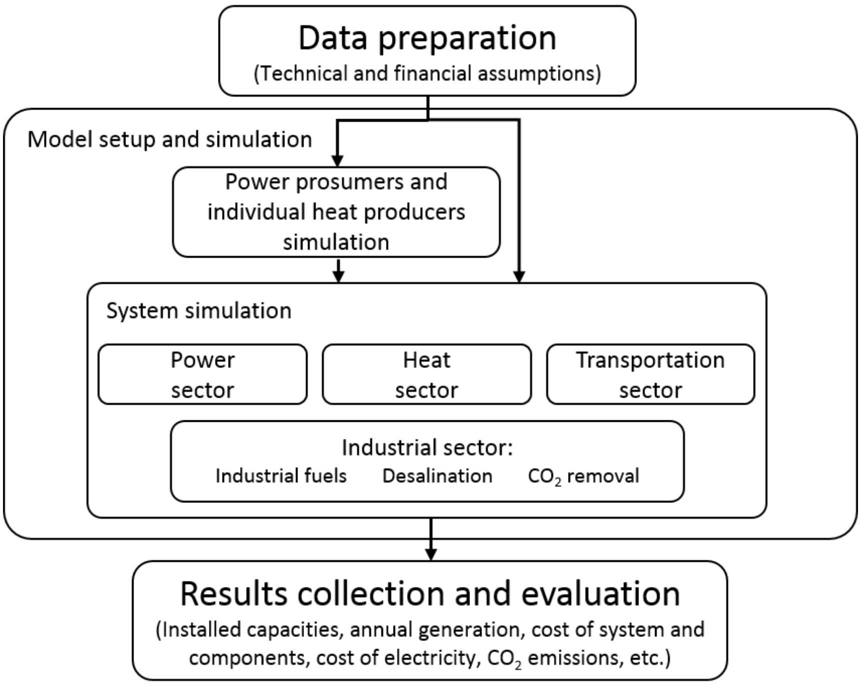

Figure S1: Fundamental structure of the LUT Energy System Transition Model, related to Star Methods.

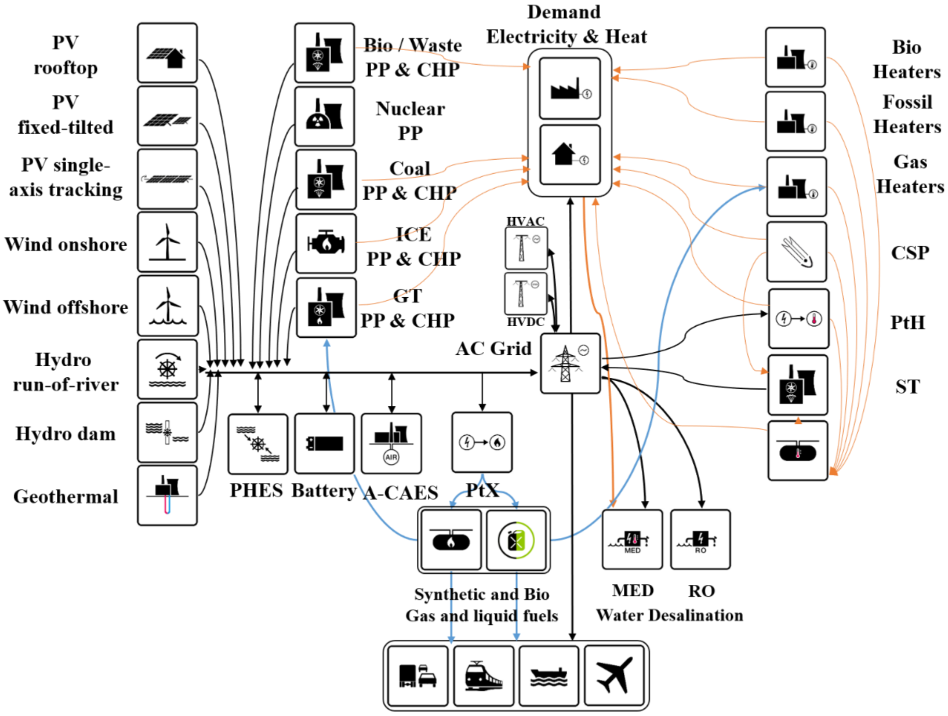

Figure S2: Schematic of the LUT Energy System Transition Model comprised of energy converters for power and heat, storage technologies, transmission options, and demand sectors, related to Star Methods.

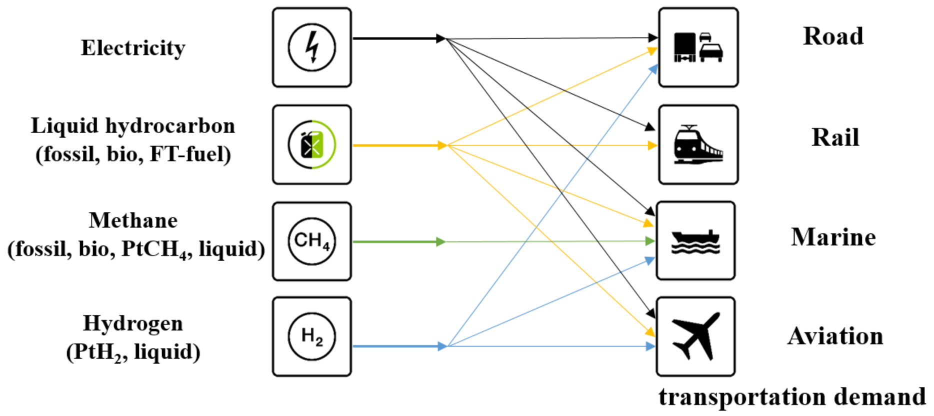

Figure S3: Schematic of the transport modes and corresponding fuels utilized during the energy transition period, related to Star Methods.

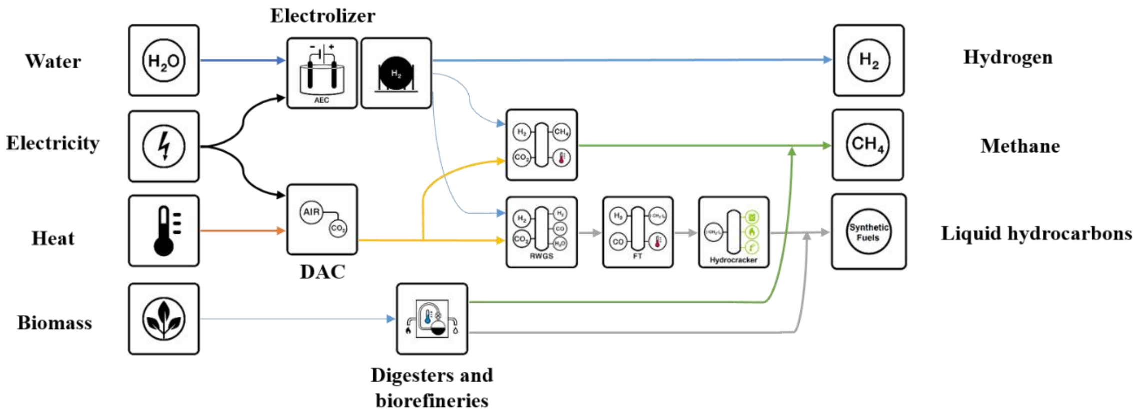

Figure S4: Schematic of the value chain elements in the production of sustainable fuels, related to Star Methods.

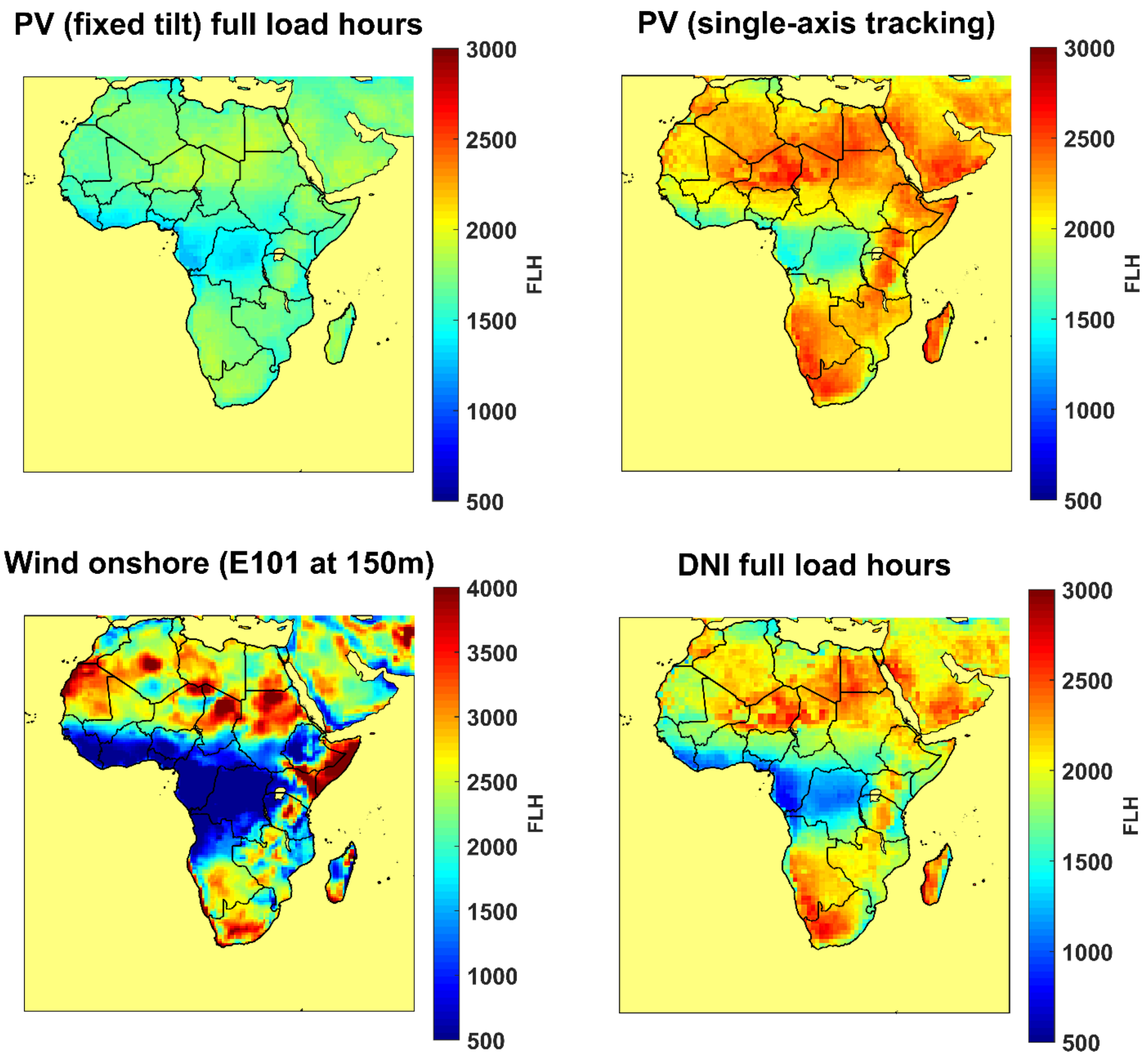

Figure S5: Resource maps for optimally tilted (top left) and single-axis tracking PV (top right), CSP solar field (bottom left), and wind power plants (bottom right) in Africa, related to Star Methods. Generation profiles are calculated based on the method described in Oyewo et al. (2021).

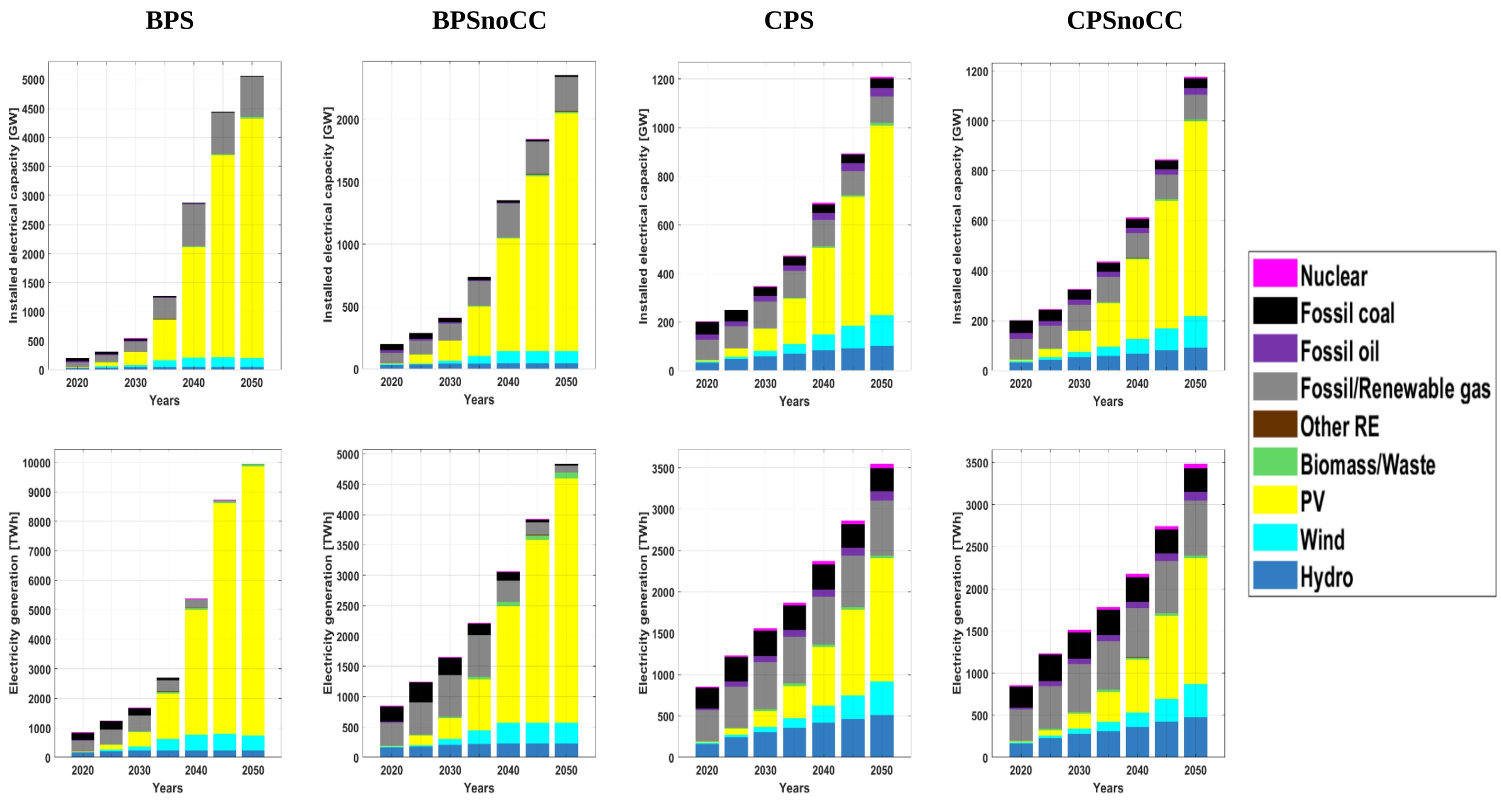

Figure S6: Installed electricity capacity (top row) and electricity generation (bottom row) from 2020 to 2050, related to Figure 2A.

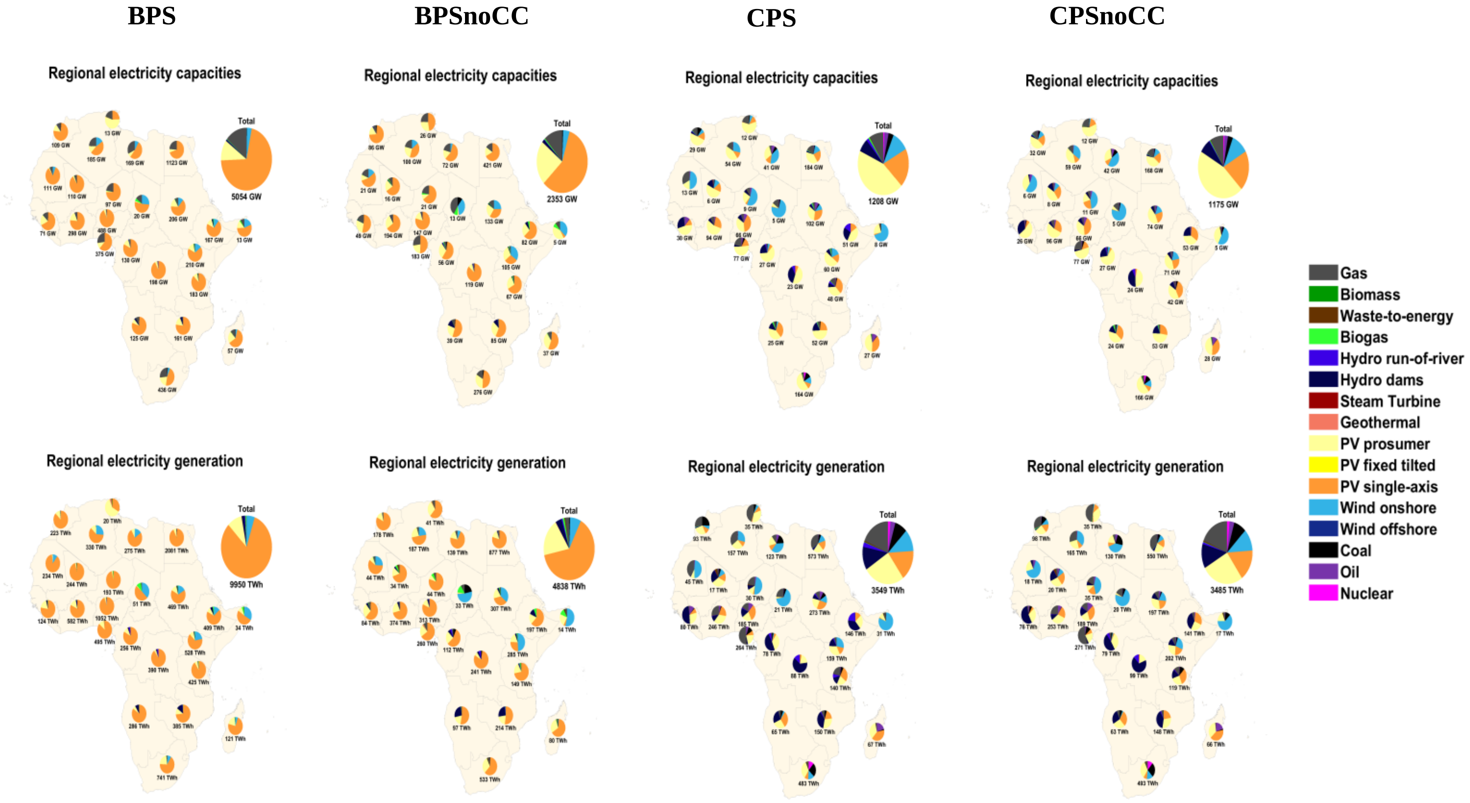

Figure S7: Regional installed electricity capacity (top row) and regional electricity generation (bottom row) for 2050, related to Figure 2 (B and C).

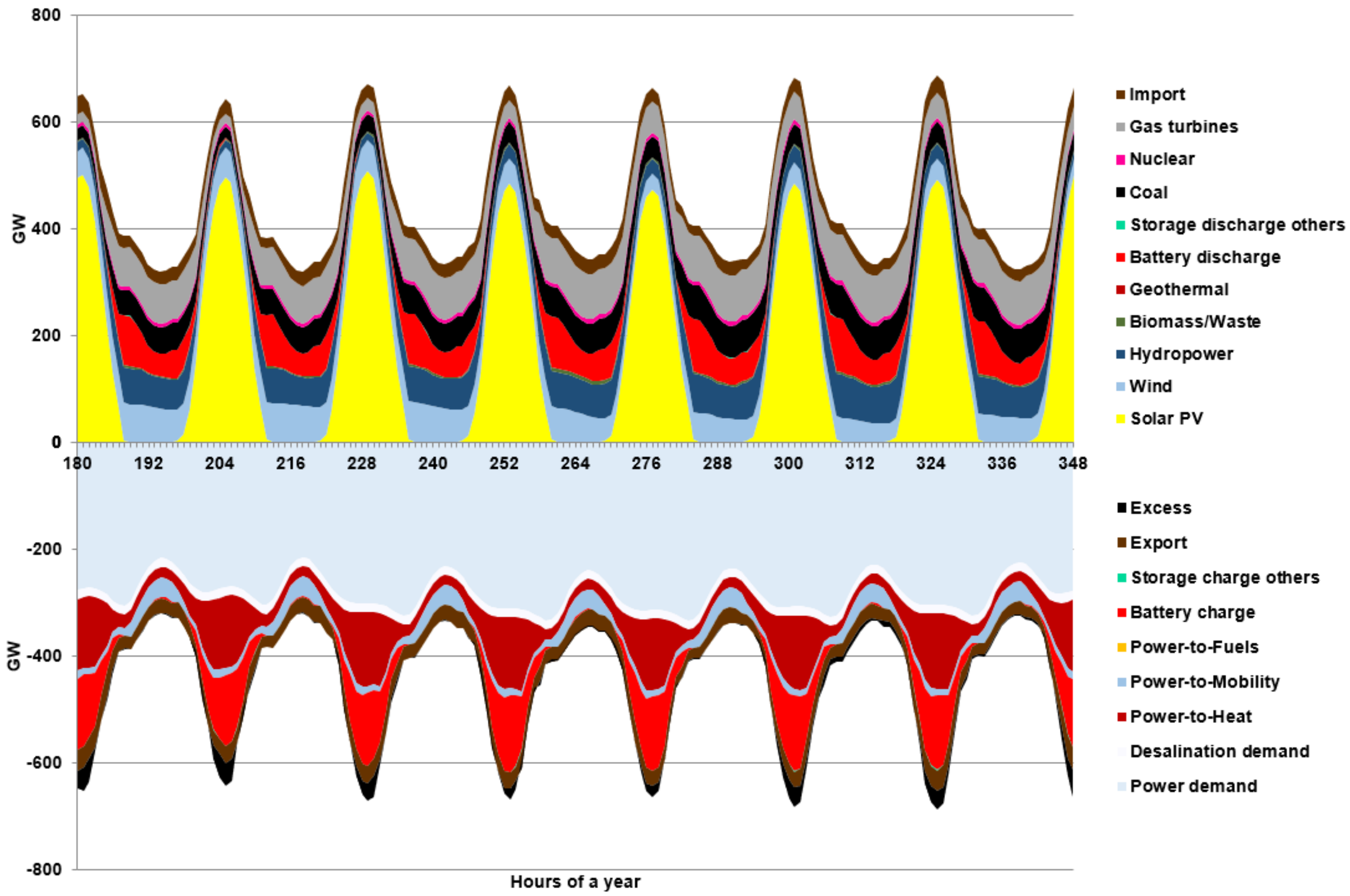

Figure S8: Hourly operation of the energy system during the worst week regarding total renewable electricity availability for the CPS, related to Figure 2A.

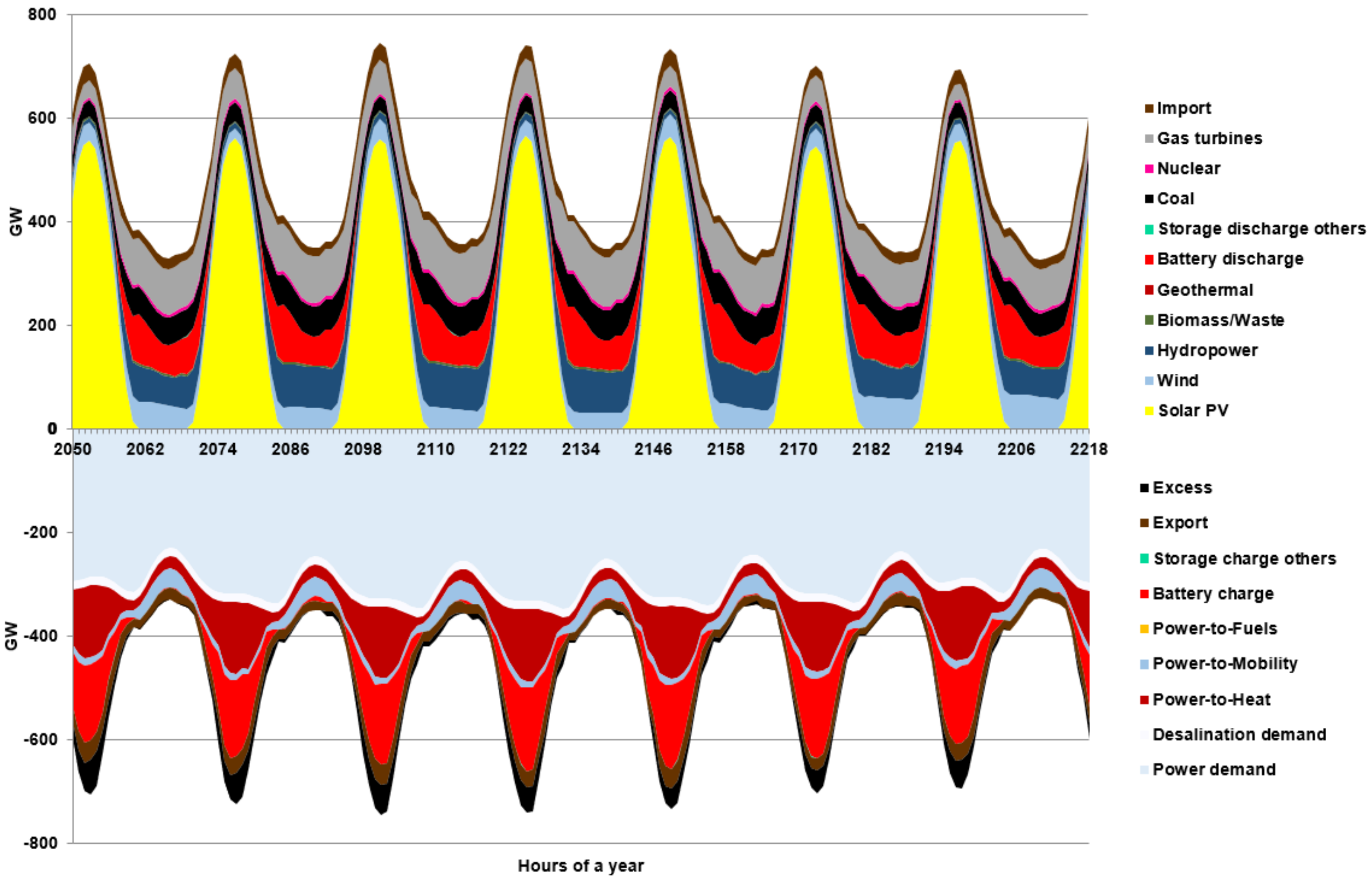

Figure S9: Hourly operation of the energy system during the best week regarding total renewable electricity availability for the CPS, related to Figure 2A.

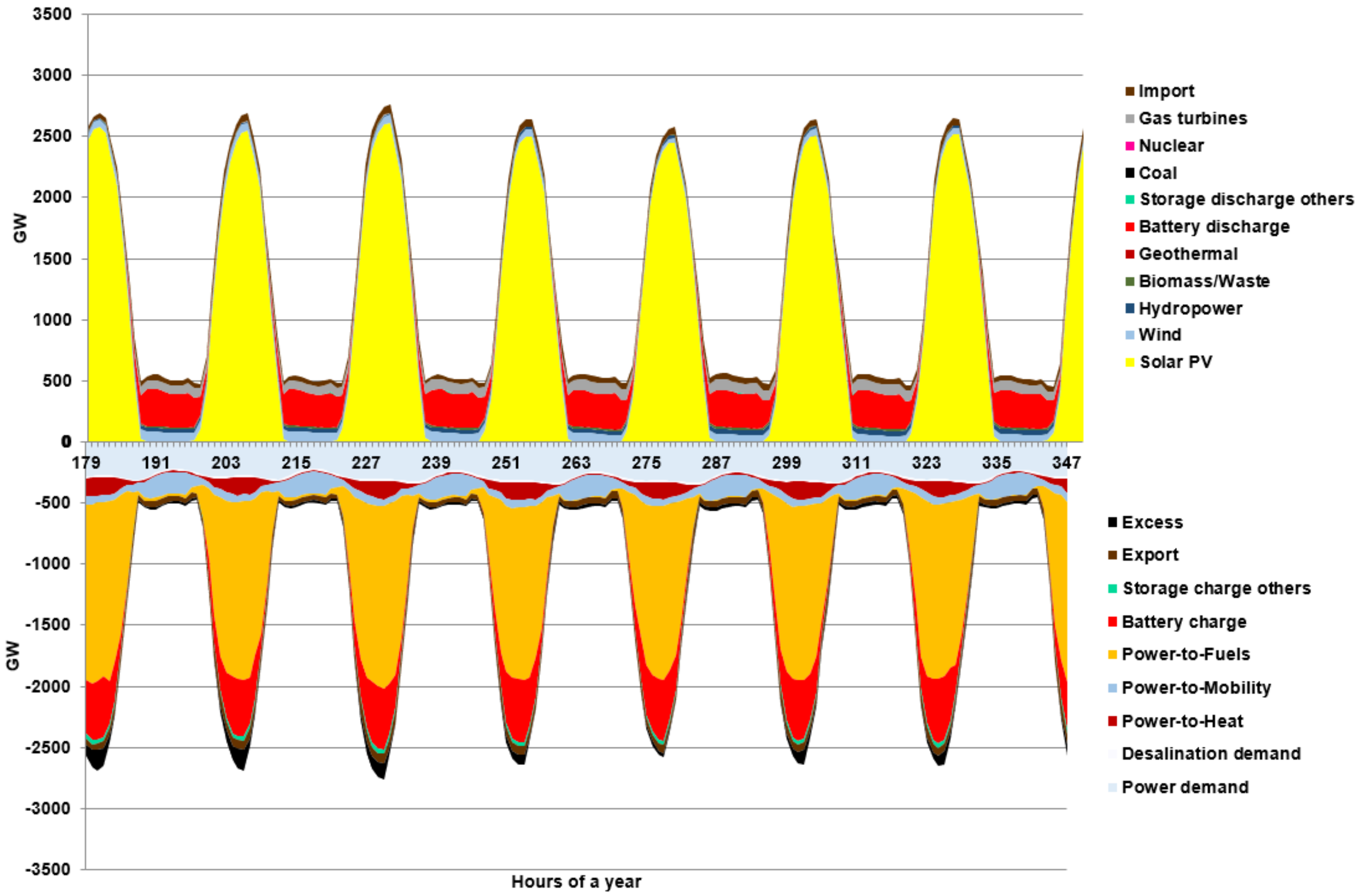

Figure S10: Hourly operation of the energy system during the worst week regarding total renewable electricity availability for the BPS, related to Figure 2A.

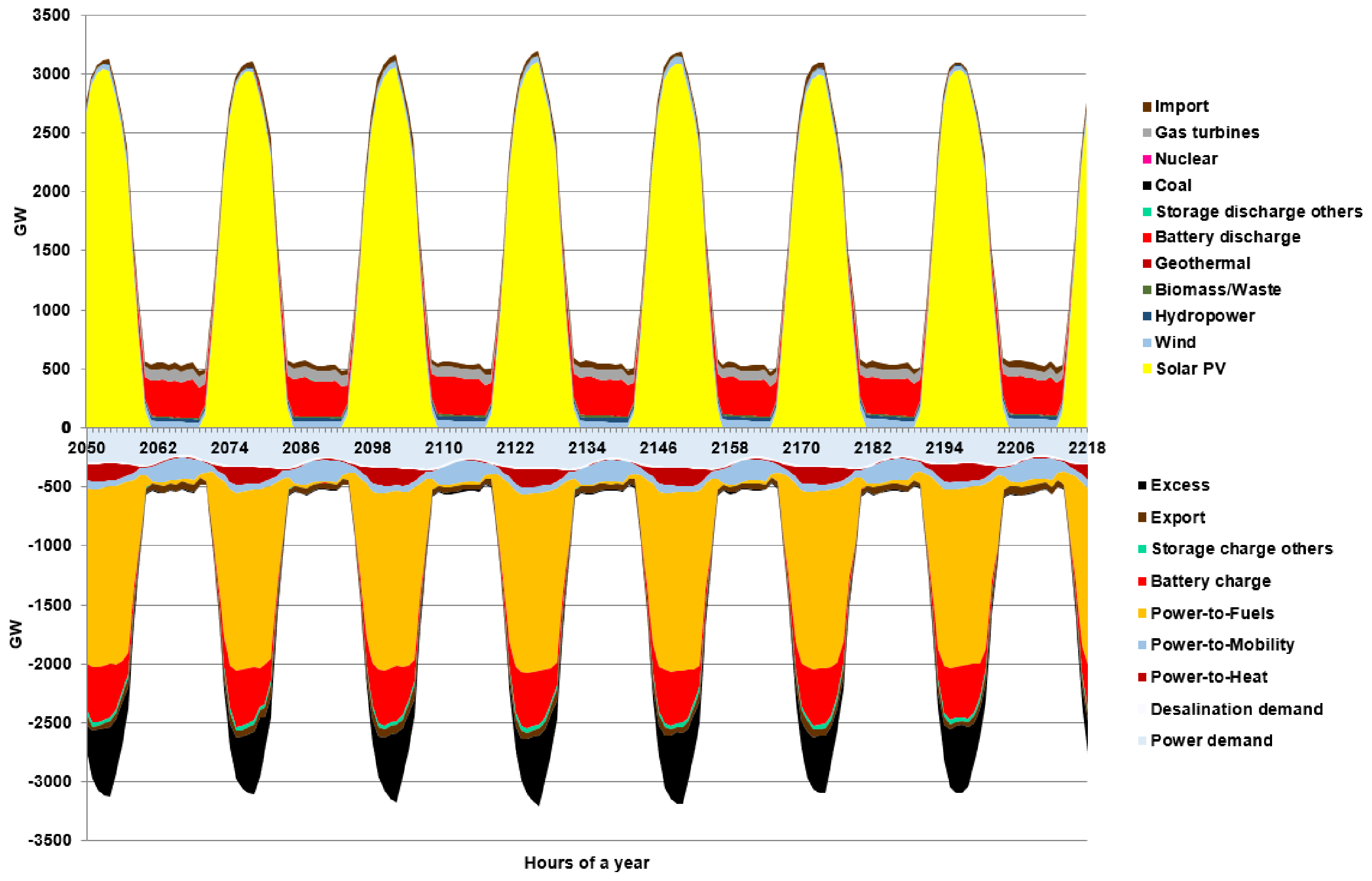

Figure S11: Hourly operation of the energy system during the best week regarding total renewable electricity availability for the BPS, related to Figure 2A.

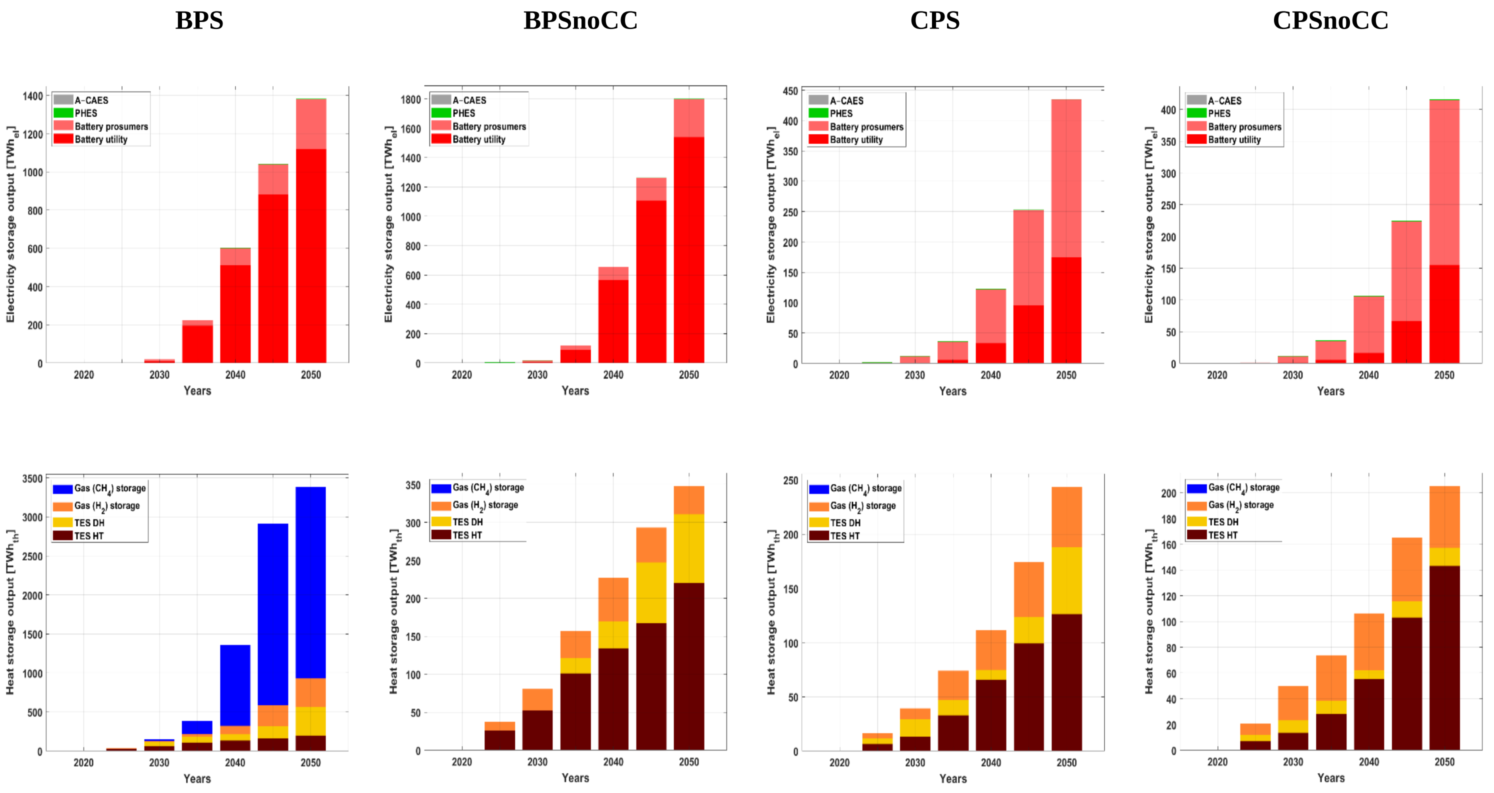

Figure S12: Electricity storage output (top row) and heat storage output (bottom row) from 2020 to 2050, related to Figure 3A.

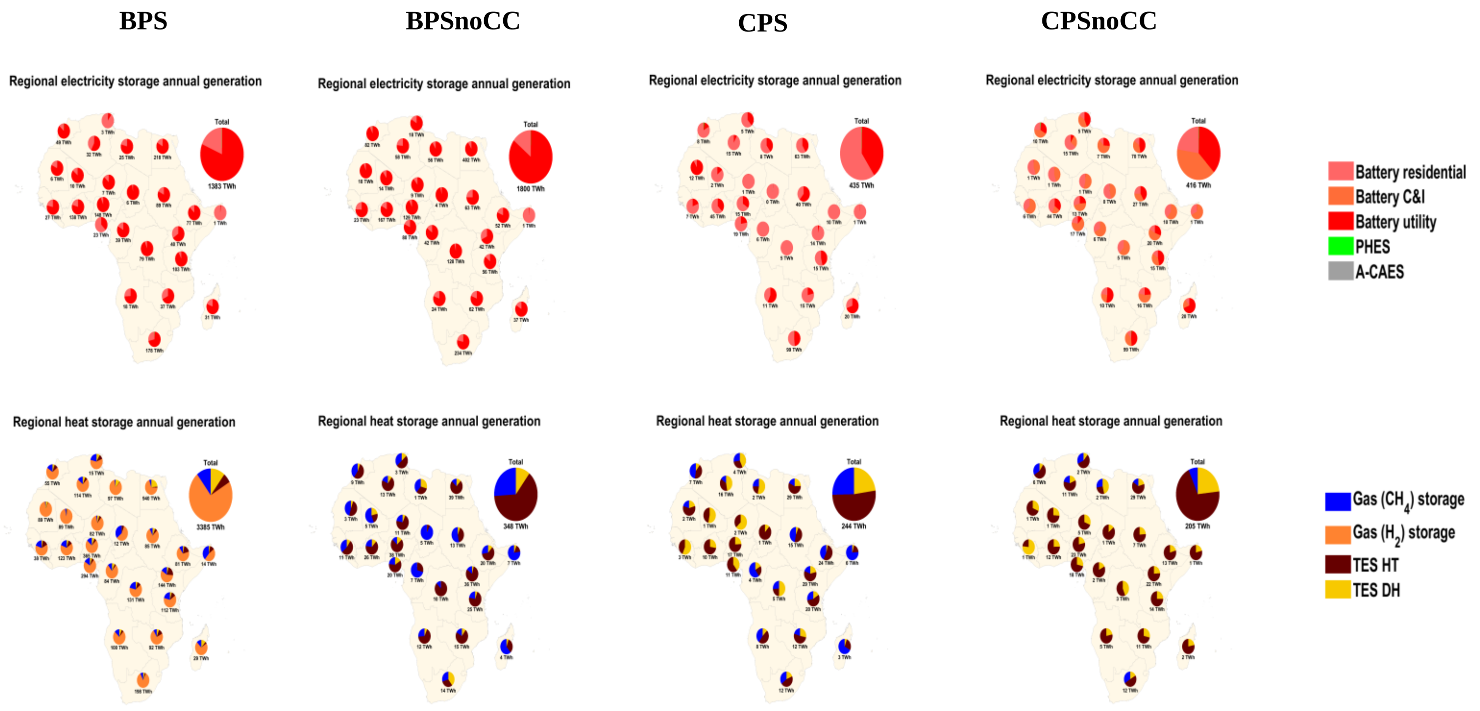

Figure S13: Regional electricity output (top row) and regional heat storage output (bottom row) for 2050, related to Figure 3A.

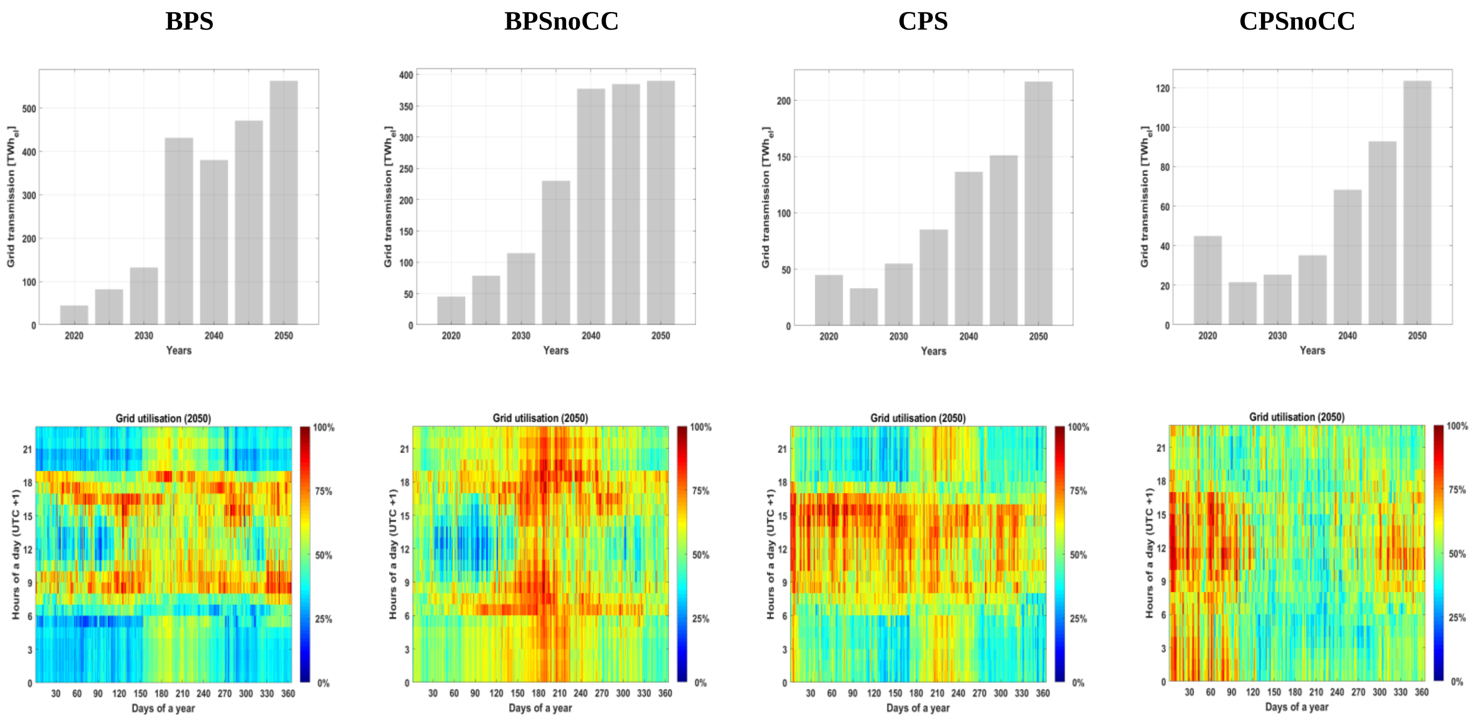

Figure S14: Grid transmission in TWh (top row) from 2020 to 2050 and grid utilisation (bottom row) for 2050, related Figure 3 (B and C).

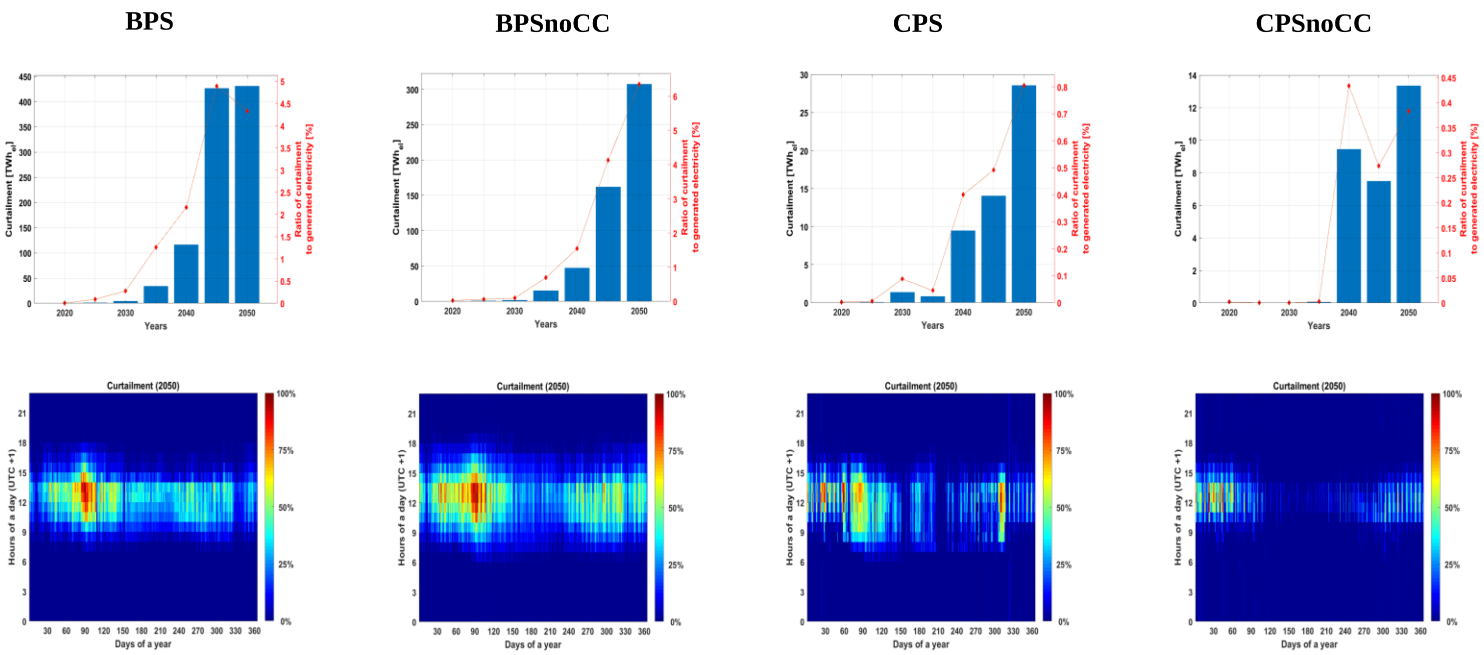

Figure S15: Curtailed generation in TWh (top row) from 2020 to 2050 and curtailment (bottom row) for 2050, related to Figure 3 (D and E)

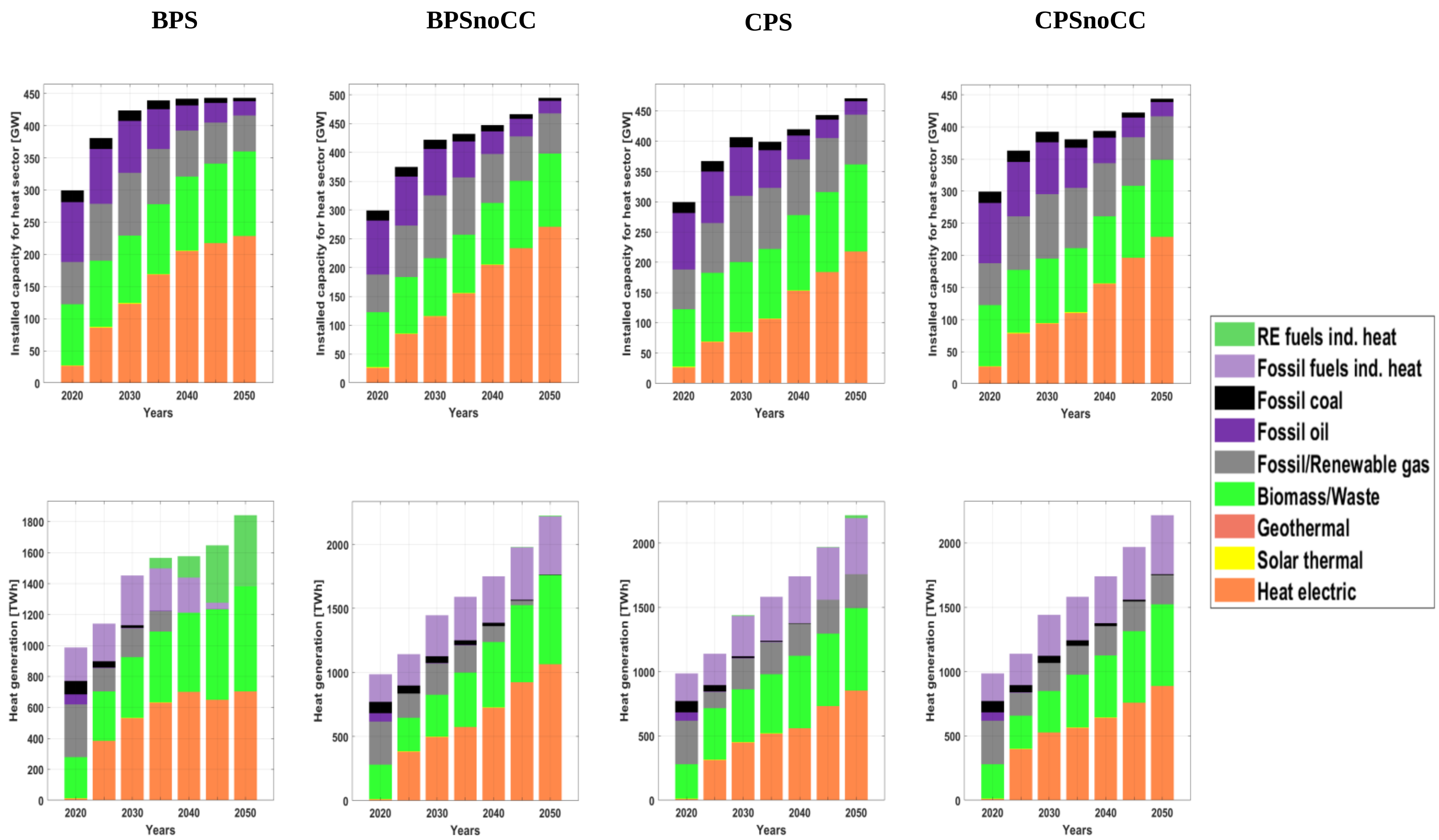

Figure S16: Installed heat capacity (top row) and heat generation (bottom row) from 2020 to 2050, related to Figure 4A.

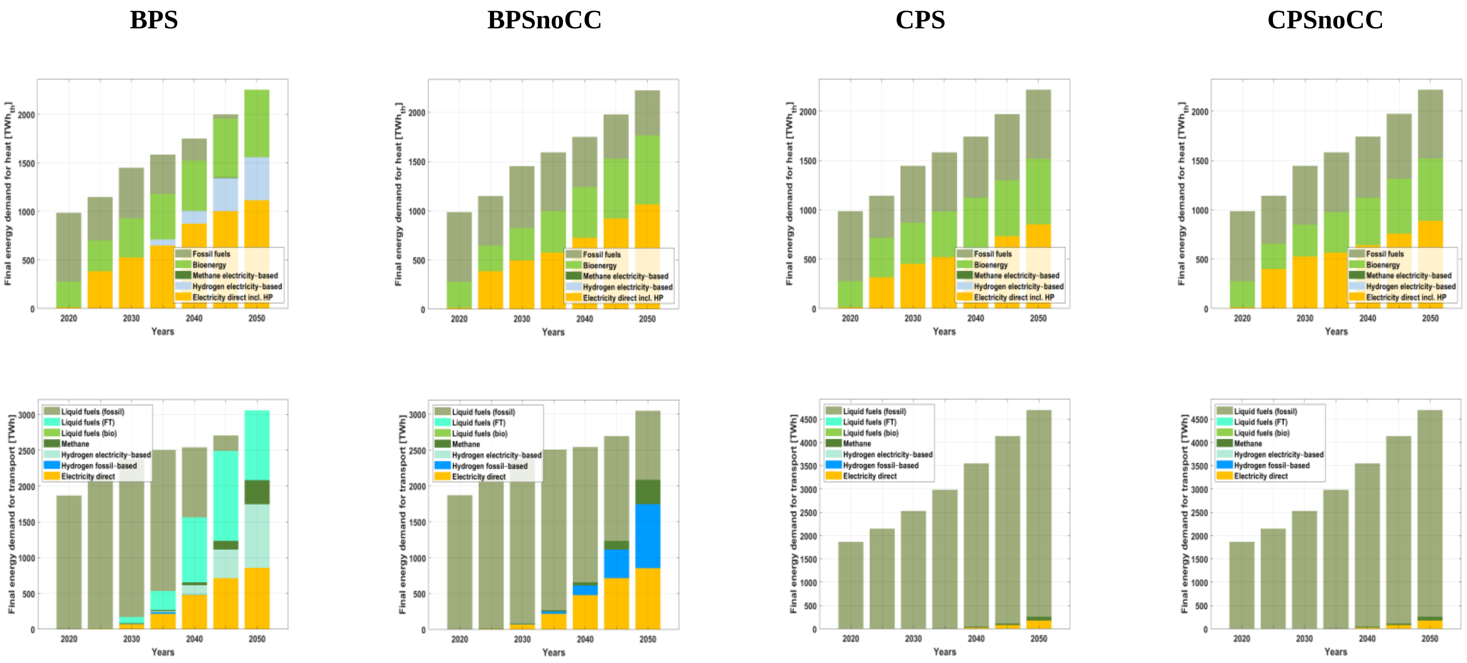

Figure S17: Final energy demand for heat (top row) and final energy demand transport (bottom row) from 2020 to 2050, related to Figure 4B.

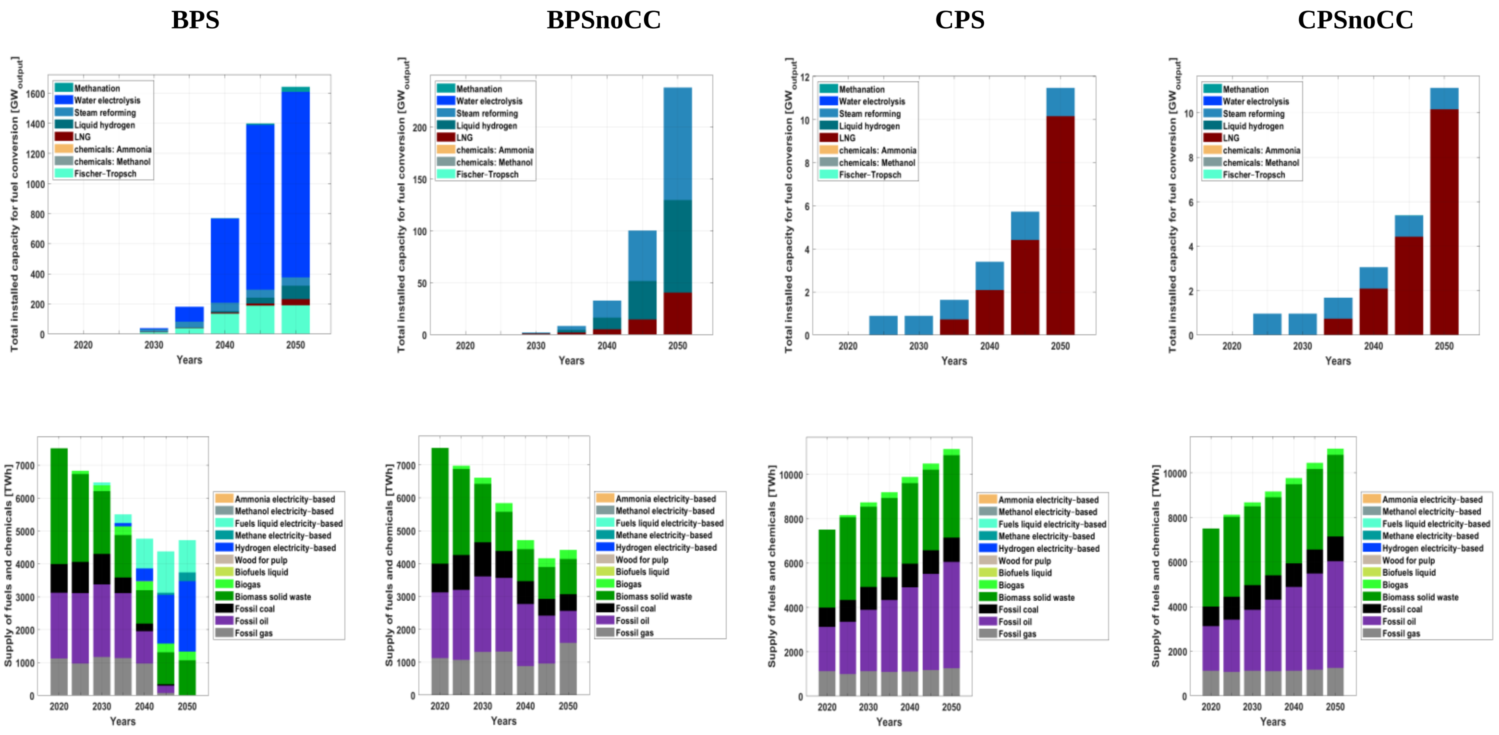

Figure S18: Installed capacity for fuel conversion (top row) and supply of fuels and chemicals (bottom row) from 2020 to 2050, related to Figure 4D.

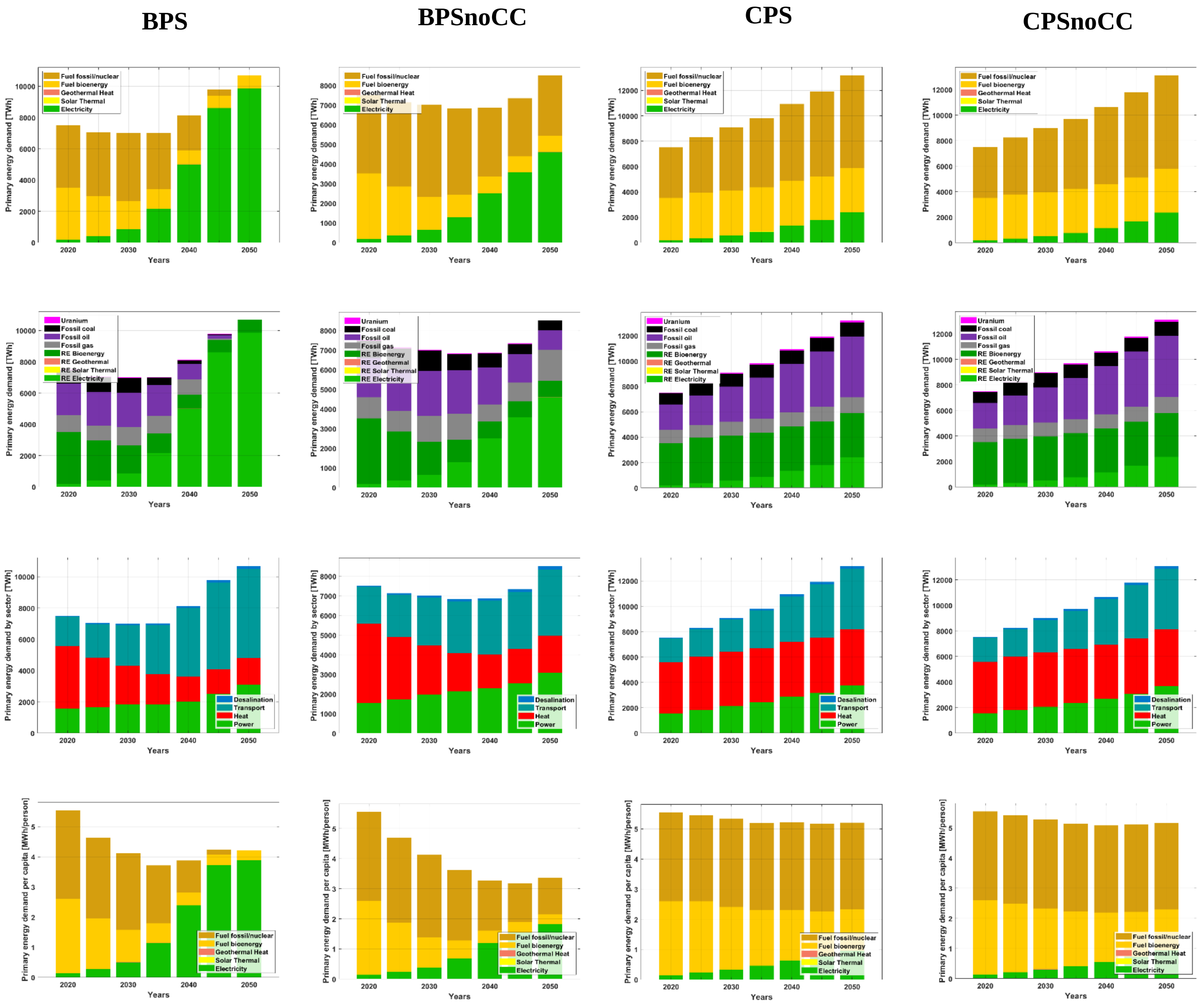

Figure S19: Primary energy demand by fuel (first row), primary energy demand by fuel (second row), primary energy demand by sector (third row) and primary energy demand per capita (fourth row) from 2020 to 2050, related to Figure 5.

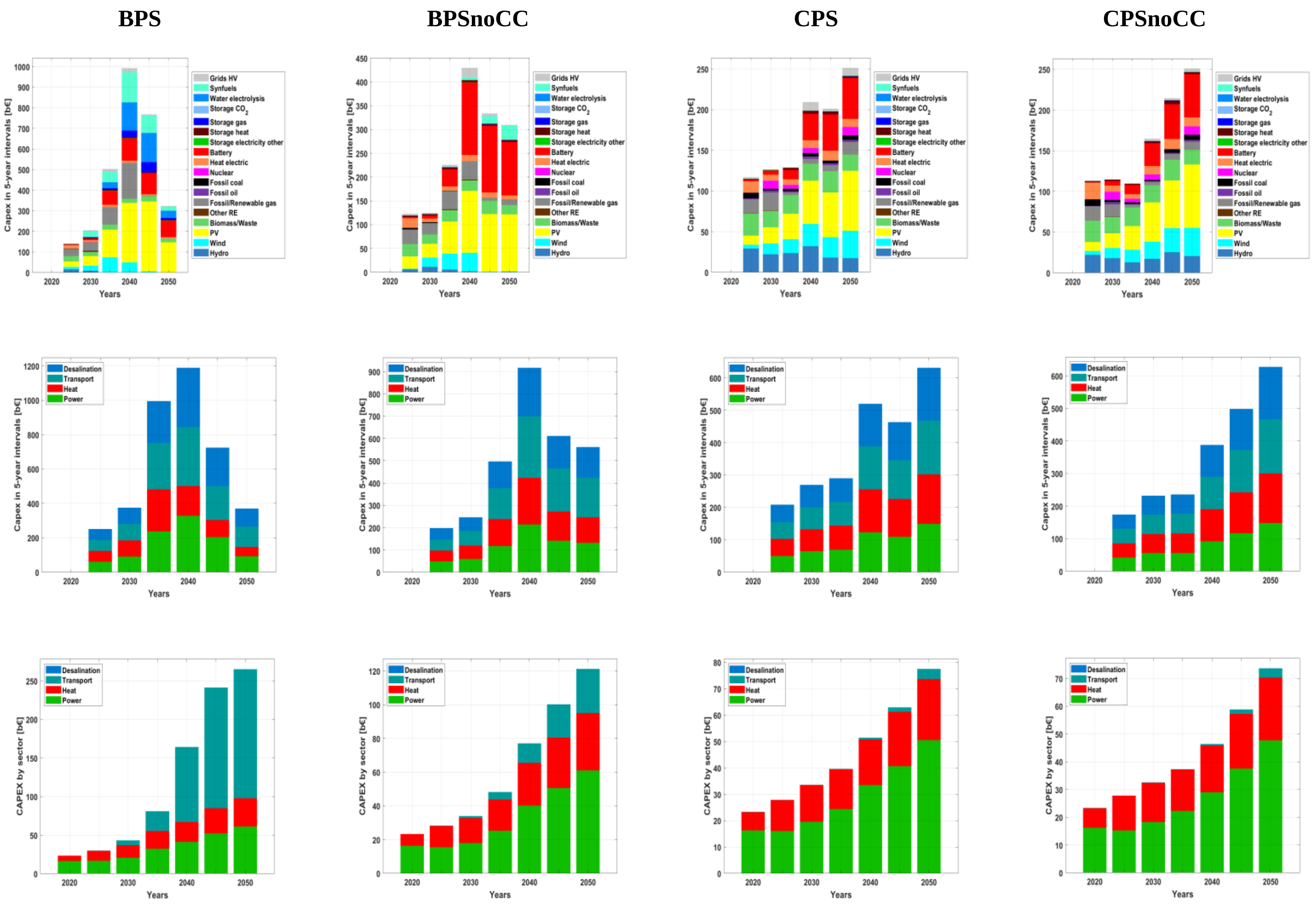

Figure S20: Capex in 5-year interval by technology (top row), Capex in 5-year interval by sector (middle row), and capex by sector (bottom row) from 2020 to 2050, related to Figure 5A.

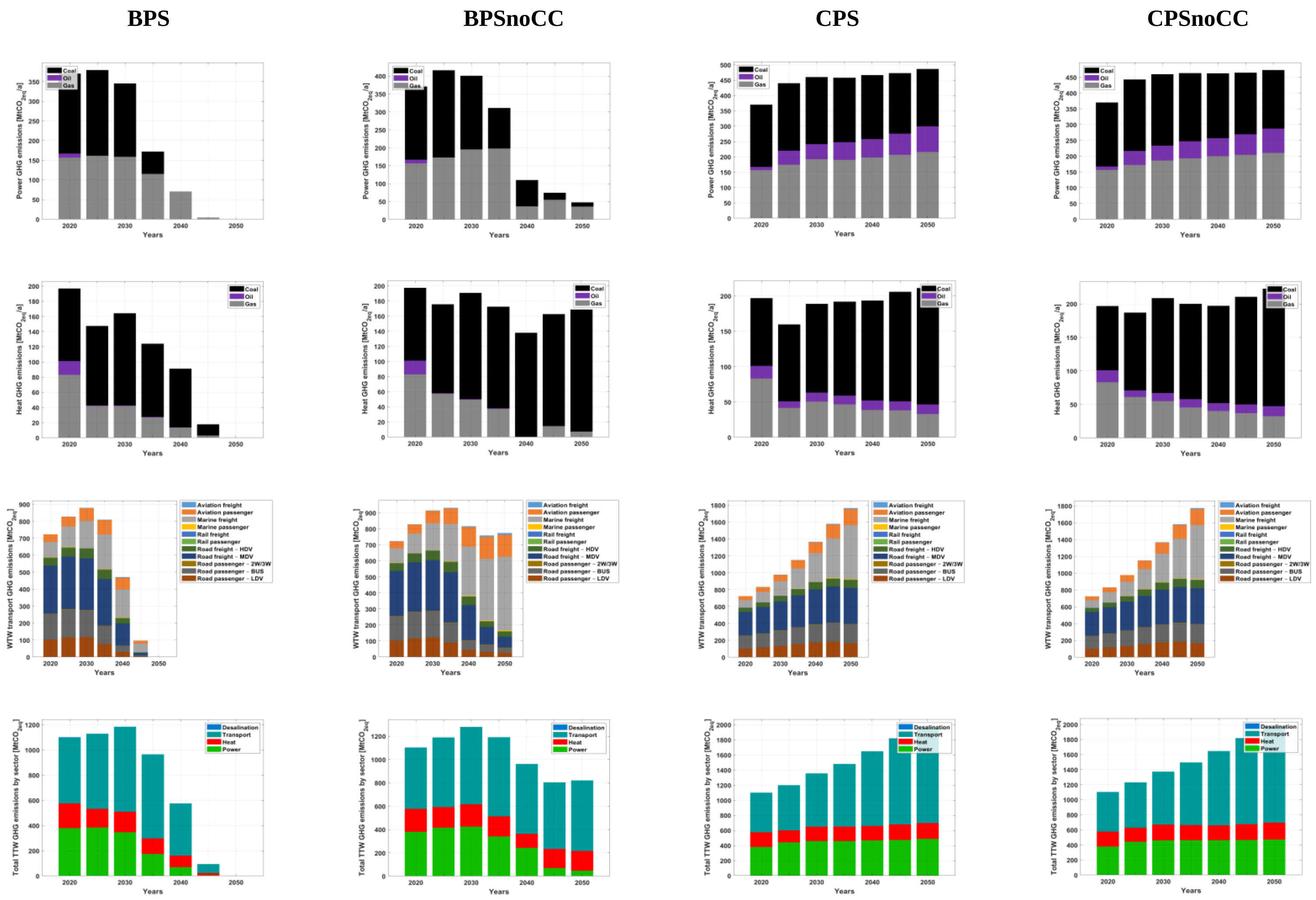

Figure S21: CO_2 emissions: power sector (first row), heat sector (second row), transport sector (third row) and sector-wise (fourth row) from 2020 to 2050, related to Figure 5C.

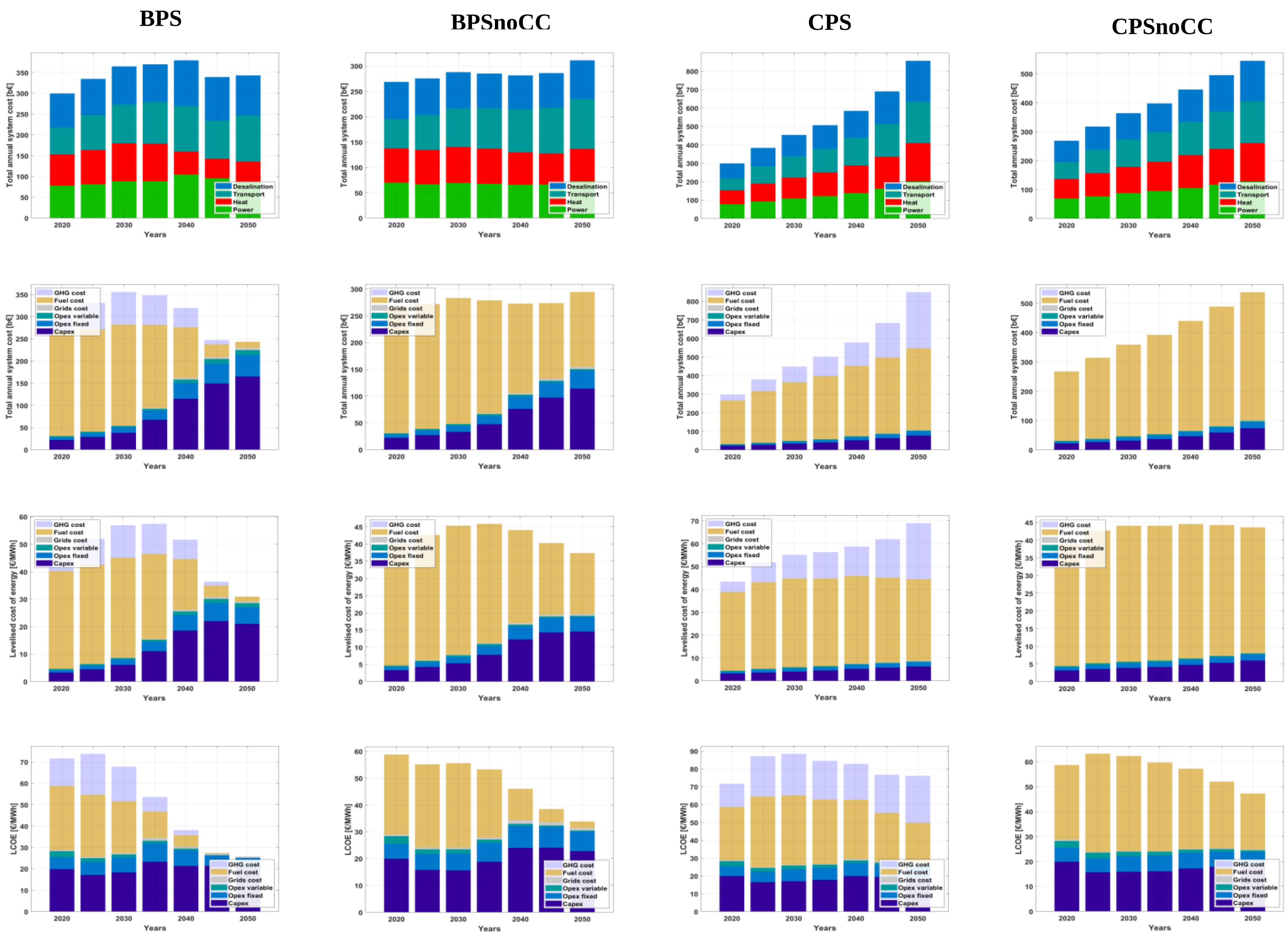

Figure S22: Total annual system cost by sector (first row), total annual system cost (second row), levelised cost of energy (third row) and levelised cost of electricity (fourth row) from 2020 to 2050, related to Figure 5 (A and B).

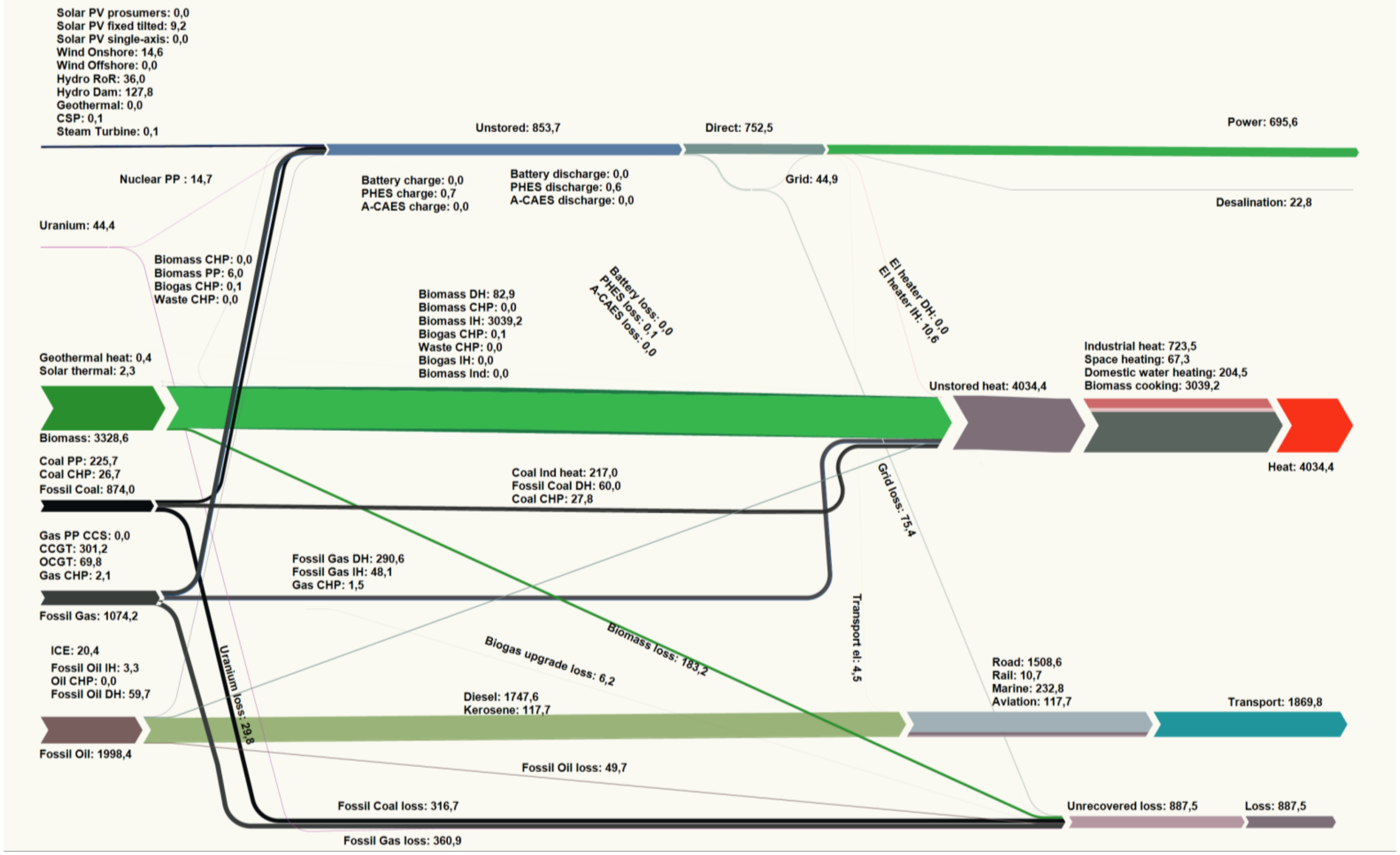

Figure S23: Energy flow of the system in 2020, all numbers displayed are in TWh, related to Table 2.

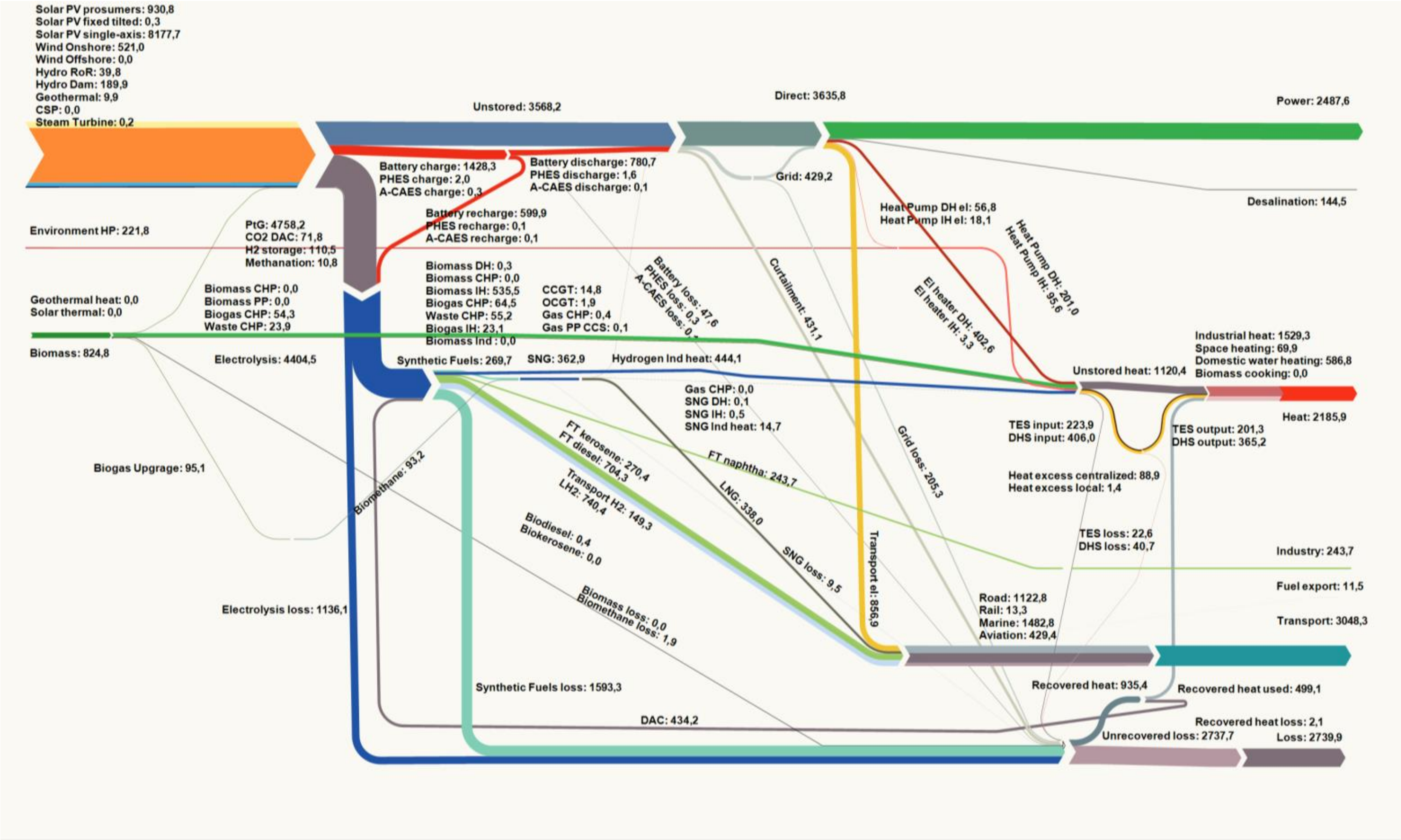

Figure S24: Energy flow of the system in 2050 for the BPS, all numbers displayed are in TWh, related to Table 2.

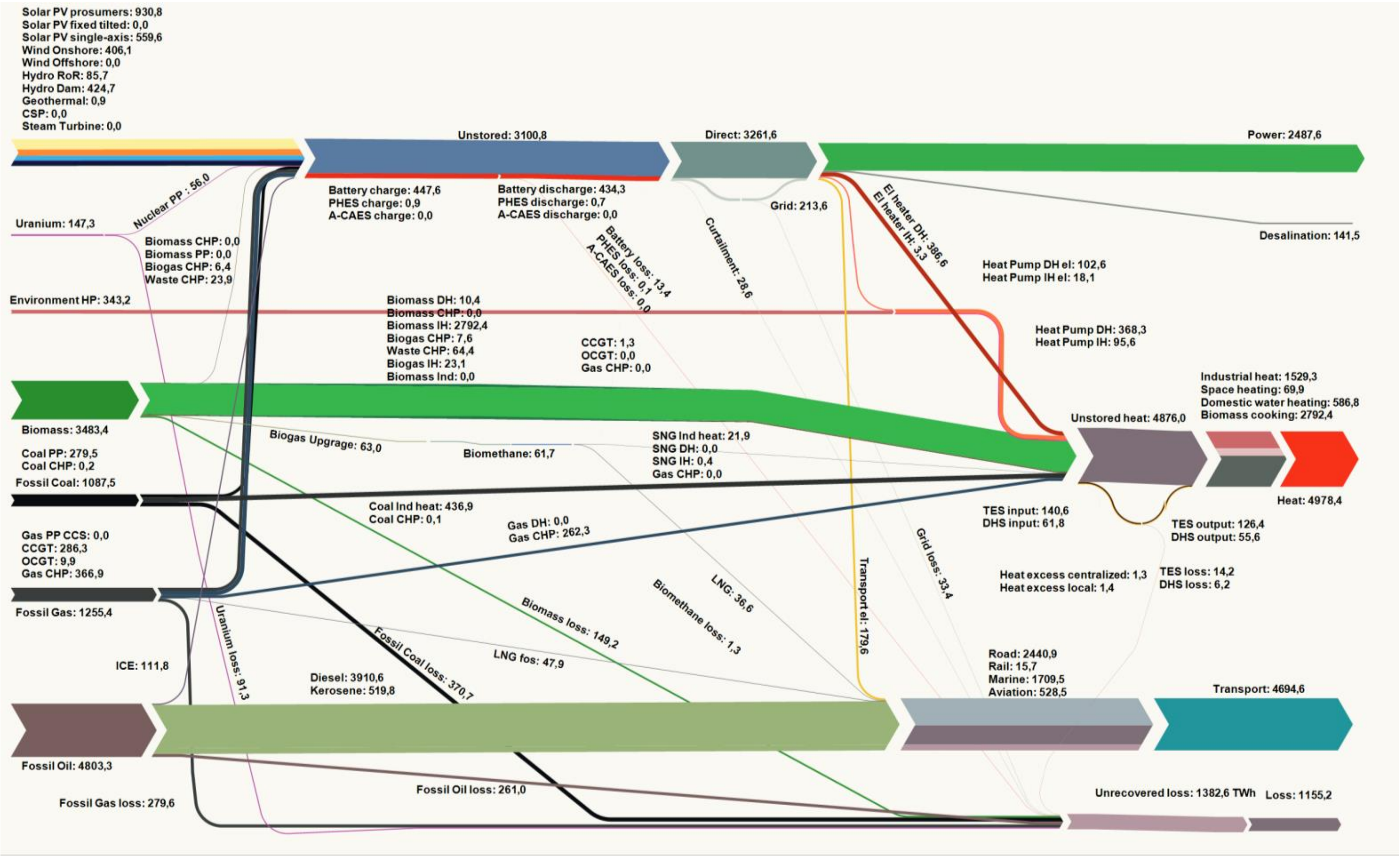

Figure S25: Energy flow of the system in 2050 for the CPS, all numbers displayed are in TWh, related to Table 2
